# Endothelial extracellular vesicle miR-423-5p regulates microvascular homeostasis and renal function after ischemia-reperfusion injury

**DOI:** 10.1172/jci.insight.181937

**Published:** 2025-05-22

**Authors:** Francis Migneault, Hyunyun Kim, Alice Doreille, Shanshan Lan, Alexis Gendron, Marie-Hélène Normand, Annie Karakeussian Rimbaud, Martin Dupont, Isabelle Bourdeau, Éric Bonneil, Julie Turgeon, Sylvie Dussault, Pierre Thibault, Mélanie Dieudé, Éric Boilard, Alain Rivard, Héloïse Cardinal, Marie-Josée Hébert

**Affiliations:** 1 Centre de Recherche, Centre Hospitalier de l’Université de Montréal (CRCHUM), Montréal, Québec, Canada.; 2 Canadian Donation and Transplantation Research Program (CDTRP), Edmonton, Alberta, Canada.; 3 Département de Microbiologie, Infectiologie et Immunologie, Faculté de Médecine, and; 4 Département de Médecine, Université de Montréal, Montréal, Québec, Canada.; 5 Institute for Research in Immunology and Cancer, Montréal, Québec, Canada.; 6 Department of Chemistry, Université de Montréal, Montréal, Québec, Canada.; 7 Héma-Québec, Québec, Québec, Canada.; 8 Centre de Recherche du, Centre Hospitalier Universitaire (CHU) de Québec-Université Laval, Département de Microbiologie et Immunologie Québec, Québec, Canada.

**Keywords:** Cell biology, Nephrology, Vascular biology, Chronic kidney disease, Endothelial cells, Noncoding RNAs

## Abstract

Microvascular rarefaction substantially contributes to renal dysfunction following ischemia-reperfusion injury (IRI). We characterized the microRNA signature of extracellular vesicles (EVs) released during endothelial apoptosis to identify biomarkers and regulators of microvascular rarefaction and renal dysfunction. Using in vitro models and RNA-Seq, we found miR-423-5p, let-7b-5p, and let-7c-5p enriched in small EVs from apoptotic endothelial cells. In mouse models of renal IRI and a cohort of 51 patients who have undergone renal transplant with delayed graft function, serum miR-423-5p correlated with circulating EVs, while let-7b-5p and let-7c-5p were also present in free form. Early acute kidney injury saw increased serum miR-423-5p levels linked to small EVs with endothelial markers. Over time, higher serum miR-423-5p levels were associated with large EVs and correlated with greater renal microvascular density and reduced fibrosis. Microvascular density and fibrosis predicted renal function 3 years after transplantation. We explored miR-423-5p’s role in renal homeostasis, finding that its injection during renal IRI preserved microvascular density and inhibited fibrosis. Endothelial cells transfected with miR-423-5p showed enhanced resistance to apoptosis, increased migration, and angiogenesis. Localized miR-423-5p injection in hindlimb ischemia model accelerated revascularization. These findings position miR-423-5p as a predictor of renal microvascular rarefaction and fibrosis, highlighting potential strategies for preserving renal function.

## Abstract 2

MicroRNA miR-423-5p is a key regulator of endothelial homeostasis and microvascular repair and a new potential target for better predicting and preventing progressive renal dysfunction.

## Graphical abstract

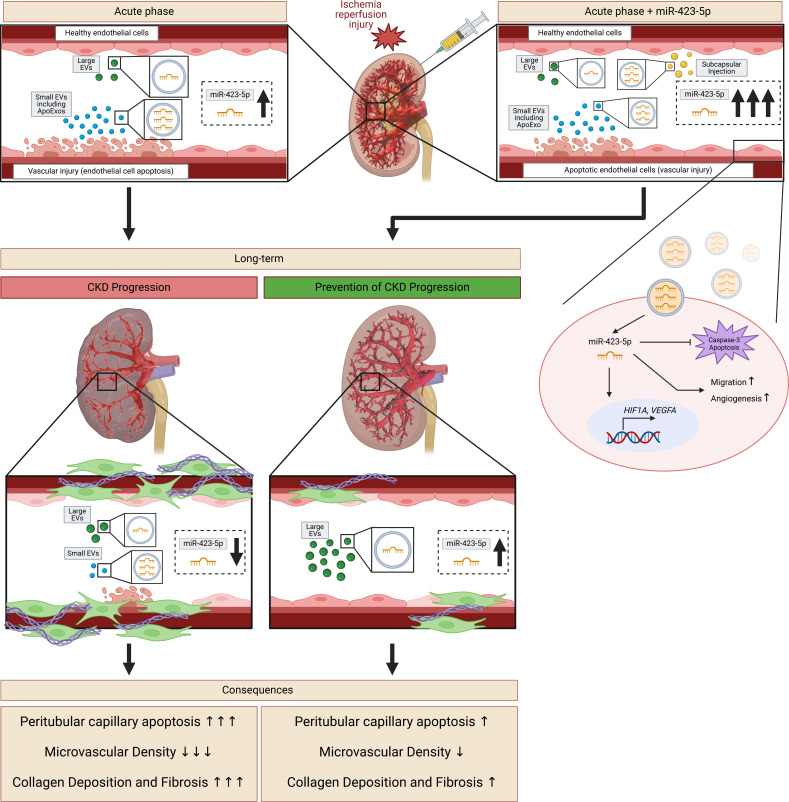

## Introduction

Renal ischemia-reperfusion injury (IRI) is an important cause of both acute kidney injury (AKI) and progressive chronic kidney disease (CKD) (1–7). IRI is an integral component of organ transplantation, and severe IRI in the perioperative transplantation setting can result in posttransplantation AKI, which manifests as delayed graft function — i.e., partial or complete incapacity of the kidney allograft to resume function after transplantation (8). The long-term effect of IRI on kidney function has been a matter of debate in the field of transplantation, as some, but not all, episodes of delayed graft function are associated with permanent reduction in graft function. Mounting evidence from different laboratories, including ours, demonstrates that the degree of microvascular injury associated with AKI is a central prognostic factor for development of long-term kidney dysfunction (9–11).

In kidney transplant recipients, loss of peritubular capillaries (PTCs) in the first months after transplantation is closely associated with fibrosis, progressive loss of renal function, and reduced allograft survival (9, 10). Animal models of renal IRI have demonstrated an association between the severity of microvascular rarefaction and long-term loss of renal function (6, 11–14). Activation of caspase-3, the major effector of apoptosis, in PTCs after AKI has been implicated in microvascular rarefaction and progressive loss of renal function (13, 15). Our group showed that caspase-3–deficient mice exposed to renal IRI show increased tubular epithelial injury in the acute phase of AKI but preservation of microvascular integrity at all stages. In the long term, this translates into reduced renal fibrosis and prevention of long-term kidney dysfunction (16, 17). Collectively, these results in animal models and in cohorts of patients who have undergone kidney transplant highlight the central importance of caspase-3–mediated microvascular injury in the pathophysiology of progressive kidney dysfunction after IRI. Unfortunately, the lack of clinically reliable biomarkers of microvascular injury is a major roadblock for assessing and preventing microvascular rarefaction in patients.

Our group has shown that caspase-3 activation in endothelial cells (ECs) controls the release of small extracellular vesicles (EVs) that differ from classical apoptotic bodies and normal exosomes by their size, markers, ultrastructure, and function and that we have coined apoptotic exosome-like vesicles (ApoExos) (18–21). Contrary to apoptotic bodies, large EVs (> 200 nm) that are produced by apoptotic cells through membrane blebbing, ApoExos originate from stress-induced autolysosomes and belong to the category of small EVs (<100 nm) (20, 22–24). In contrast to the tolerogenic function of apoptotic bodies, ApoExos display strong immunogenic activity dependent, at least in part on the presence of active 20S proteasome. They also cargo perlecan/LG3 and lysosomal LAMP2 (18–22, 25). ApoExos carry nucleic acids largely in the form of noncoding RNAs (21, 25), but their microRNA (miRNA) signature, if they have one, has yet to be characterized. EV-cargoed miRNAs are increasingly studied as potential biomarkers of disease because of their stability while in circulation and because of the relative ease of monitoring their levels in biological fluids with technology already in use in the clinic, such as PCR (26).

The goal of the present study was to define the miRNA signature of endothelial ApoExos in vitro and in vivo, aiming to identify biomarkers and regulators of microvascular injury and to assess their functional importance in regulating microvascular rarefaction and fibrosis after renal IRI.

## Results

### Small EVs produced by apoptotic ECs are enriched in small RNAs.

**Figure 1:**
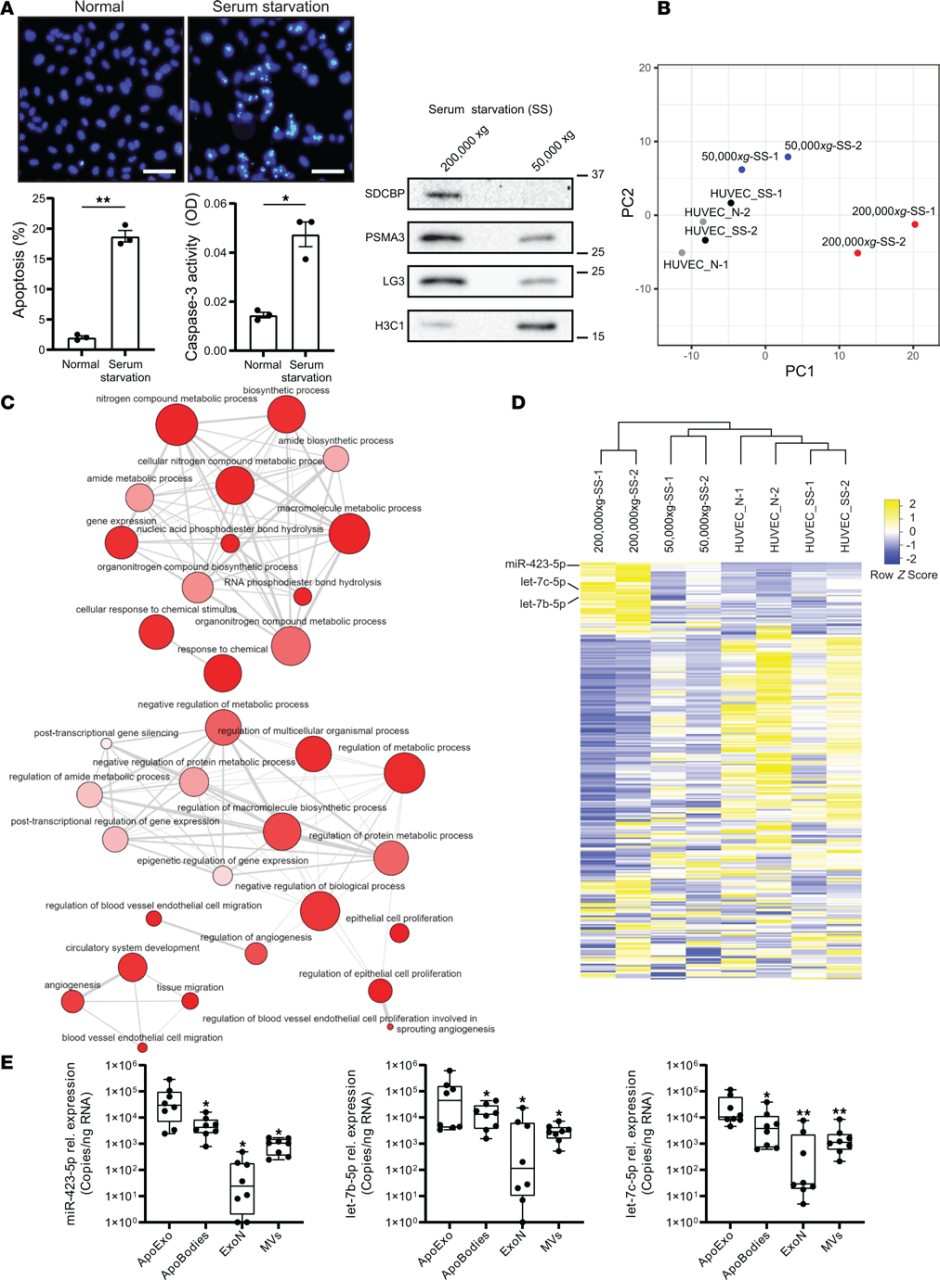
Specific microRNA signature of apoptotic exosome-like vesicles (ApoExos). (**A**) Apoptosis in HUVECs exposed to normal medium (N) or serum-starved (SS) for 4 hours is expressed as the percentage of apoptotic cells ± SEM, along with caspase-3 activity. Scale bar: 200 μm. *P* values obtained by unpaired *t* test (*P < 0.05, **P < 0.01); *n* = 3 for each condition. Western blots show protein markers in large (50,000*g*) and small (200,000*g*) extracellular vesicles (EVs) purified from media conditioned by serum-starved HUVECs. Apoptotic bodies (ApoBodies) and ApoExos are recovered from large and small vesicle fractions, respectively (*n* = 8). MW expressed in kDa. (**B**) Principal component analysis (PCA) using small RNAs in ApoBodies (50,000*g*-SS) and ApoExos (200,000*g*-SS) from SS HUVEC media and small RNAs from cells under normal (HUVEC_N) or serum-starved (HUVEC_SS) conditions; *n* = 2. (**C**) Enrichment network of biological process GO terms. Visualization of GO terms with a *P* < 0.05 using Reduced + Visualized Gene Ontology (Revigo) software. *P* value and observed frequency of the GO term are represented by the color and size of the circles, respectively. The strongest GO term pairwise similarities are designated as edges in the network. (**D**) Heatmap of small RNA in ApoExos, ApoBodies, and HUVEC_N or HUVEC_SS; *n* = 2. (**E**) miRNA in small and large EV fractions derived from apoptotic HUVECs (small EVs: ApoExos; large EVs: ApoBodies) or healthy HUVECs (small EVs: normal exosomes [ExoN]; large EVs: normal microvesicles [MVs]). miRNA expression was measured by qPCR presented as relative copy expression per ng of RNA ± SEM after normalization to cel-miR-39; *n* = 8 biological replicates from separate EV preparations. *P* values obtained by 1-way ANOVA and the Bonferroni post hoc test (*P < 0.05, **P < 0.01).

We first exposed HUVECs to serum starvation for 4 hours to induce an apoptotic response, as assessed by evidence of activation of caspase-3 and fluorescence microscopy findings showing chromatin condensation in the absence of necrotic features (Figure 1A). Then, we set out to characterize the miRNA profile of small EVs released by apoptotic ECs. To this end, we purified small and large EVs released by serum-starved apoptotic ECs in vitro using sequential centrifugation. We confirmed that markers of ApoExos, such as proteasome and LG3, are enriched in fractions containing small EVs whereas histone, a marker of apoptotic bodies is expressed in fractions containing large endothelial vesicles (Figure 1A and Supplemental Figure 1A; supple mental material available online with this article; https://doi.org/10.1172/jci.insight.181937DS1). We then used RNA-Seq to characterize the miRNA profile of small and large EV fractions as well as the miRNA profile of ECs either serum starved or maintained in normal conditions in vitro. Principal component analysis (PCA) of miRNA expression showed that miRNA profiles recovered in ApoExos were strikingly different from those found in apoptotic bodies. They were also distinct from those found in ECs (either serum starved or normal) (Figure 1B). These results confirm that ApoExos represent a category of EVs distinct from classic apoptotic bodies. We then performed a Gene Ontology (GO) enrichment analysis coupled to Reduced + Visualized GO (Revigo) software to gain further insights into the biological processes potentially modulated by miRNAs enriched in ApoExos. We found strong signals for pathways regulating circulatory system development, endothelial proliferation, migration, and angiogenesis as well as regulation of biosynthetic and metabolic processes (Figure 1C).

We then focused on miRNAs overexpressed in ApoExos (with FPKM > 6,000 and the expression fold change > 2 for both biological replicates, when compared with apoptotic bodies as well as with serum-starved and normal ECs). Three miRNAs (miR-423-5p, let-7b-5p, and let-7c-5p) met these selection criteria, and among them, miR-423-5p was the most highly enriched as represented in the hierarchical clustering (Figure 1D and Supplemental Table 1). We then used sequential centrifugation (50,000*g* for 15 minutes followed by 200,000*g* for 18 hours) and quantitative PCR (qPCR) to compare miR-423-5p, let-7b-5p, and let-7c-5p levels in small and large EVs produced by apoptotic ECs as well as levels in small (exosome) and large (microvesicles) EVs produced by healthy ECs. qPCR confirmed that the expression levels of all 3 miRNAs were highest in fractions containing ApoExo, followed by fractions containing large EVs produced by either ECs maintained in normal culture conditions or those serum starved (Figure 1, D and E). We also assessed miR-423-5p levels within purified ApoExos originating from renal tubular epithelial cells under serum starvation. Although renal tubular epithelial cells undergoing apoptosis can release small EVs bearing ApoExo markers, such as the 20S proteasome (18), we found that ApoExos released by renal epithelial cells did not overexpress miR-423-5p, let-7b-5p, or let-7c-5p (Supplemental Figure 1, B and C). Collectively, these results identify miR-423-5p, let-7b-5p, and let-7-c-5p as markers of endothelial EVs.

### miR-423-5p serum levels predict microvascular rarefaction after AKI.

**Figure 2:**
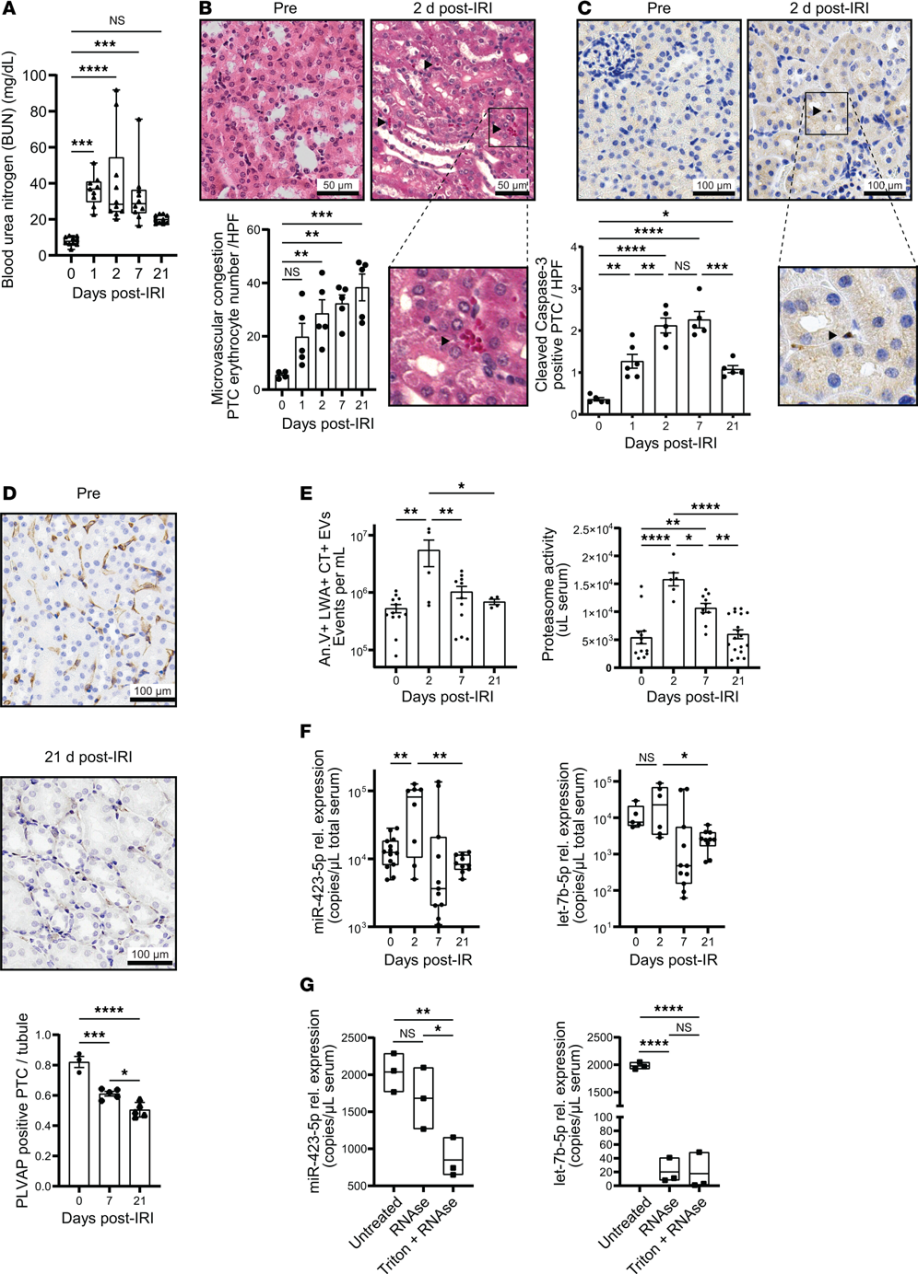
Assessment of renal injury, circulating extracellular vesicles, and circulating microRNA levels after renal IRI in mice. (**A**) Blood urea nitrogen (BUN) concentration in mice at baseline, 1, 2, 7, or 21 days after renal ischemia-reperfusion injury (IRI); *n* = 9–10 (**B**) Top: H&E-stained corticomedullary junction at 2 days after IRI. Arrowhead: Rouleaux formation. Bottom: Rouleaux formation expressed as the number of erythrocytes within peritubular capillaries (PTC) per high-power field (HPF) in corticomedullary junction; *n* = 4–5. (**C**) Top: Cleaved caspase-3 IHC in corticomedullary junction presurgery and at 2 days after IRI. Bottom: Quantification of cleaved caspase-3^+^ PTC in renal sections; *n* = 5–6. Arrowhead: cleaved caspase-3^+^ PTC. (**D**) Top: PLVAP IHC in corticomedullary junction before surgery and at 21 days after IRI. Bottom: Quantification of PLVAP^+^ PTC per tubule in corticomedullary junction; *n* = 3–5. (**E**) Quantification of CellTrace (CT) + annexin V (AnV) + Proteasome (LWA) + extracellular vesicles (100–1000 nm) by small particle flow cytometry (*n* = 4–12) and proteasome caspase-like activity in exosome-like vesicles (*n* = 6–18) from serum. (**F**) Quantification of miR-423-5p and let-7b-5p serum levels; *n* = 5–14. Data are shown as mean ± SEM. (**G**) Quantification of miR-423-5p and let-7b-5p serum levels in WT mice at baseline. Serum was treated or not with RNase A (0.025 mg/mL) with or without Triton X-100 (0.1 %) for 20 minutes at 37°C; *n* = 3. Expression of miRNAs is presented as relative copies of miRNAs per μL of total serum ± SEM. *P* values obtained by 1-way ANOVA and the Bonferroni (**A**–**F**) or Tukey (**G**) post hoc test (*P < 0.05, **P < 0.01, ***P < 0.001, ****P < 0.0001). Scale bars: 50 μm (**B**), 100 μm (**C** and **D**).

We previously showed that vascular injury in mice prompts the release of ApoExos into circulation (18). Here, we evaluated whether circulating miR-423-5p, let-7b-5p, and let-7c-5p levels in vivo after renal IRI behave as other ApoExo markers, such as LG3 and 20S proteasome. Renal artery clamping for 30 minutes followed by reperfusion induced significant renal dysfunction with an early increase in blood urea nitrogen (BUN) levels returning to baseline after 21 days (Figure 2A). Rouleaux formation, a marker of microvascular congestion, increased progressively after renal IRI for up to 21 days (Figure 2B). Caspase-3 activation in PTCs increased on the first day after IRI, plateaued on days 2 and 7, and decreased but remained elevated on day 21 after IRI when compared with baseline (Figure 2C). This was associated, at 7 and 21 days after IRI, by a decline in plasmalemma vesicle-associated protein (PLVAP) staining, a marker of microvascular ECs (Figure 2D), indicating progressive microvascular rarefaction.

We monitored circulating ApoExo levels after renal IRI with 2 different methods assessing 20S proteasome activity: measurement of caspase-like proteasome activity in serum fractions of small EVs purified by sequential centrifugation and small particle flow cytometry using a fluorescent probe for active 20S proteasome. Both methods showed increased levels of proteasome activity in the first 2 days after renal IRI followed by a progressive decline at 7 and 21 days (Figure 2E). Small particle flow cytometry confirmed that increased proteasome activity was recovered within annexin V^+^ and proteasome^+^ small EVs (Figure 2E). miR-423-5p, let-7b-5p, and let-7c-5p assessed in total nonfractionated serum followed a similar pattern (Figure 2F and Supplemental Figure 2A), and their levels were highly correlated with one another (Supplemental Figure 2B). miR-361-5p, a negative control that was not identified within ApoExo by RNA-Seq, was not modulated in the early phase of AKI (Supplemental Figure 2C).

We then evaluated the proportion of total serum miR-423-5p, let-7b-5p, and let-7c-5p that are accounted for by EVs. To this end, serum was treated with RNase with or without Triton X-100 and miR-423-5p, let-7b-5p, and let-7c-5p levels were assessed by qPCR. MiRNAs cargoed by EVs were not degraded by RNase treatment in the absence of Triton X-100 whereas free RNA was degraded. RNase treatment without Triton X-100 did not significantly reduce miR-423-5p, but reduced significantly let-7b-5p and let-7c-5p levels. This suggests that the majority of circulating miR-423-5p is cargoed by EVs while let-7b-5p and let-7c-5p are found both in EVs and in vesicle-free form (Figure 2G and Supplemental Figure 2D). Since miR-423-5p was the most enriched in endothelial ApoExo in vitro and showed circulating levels in vivo that closely parallel those of circulating small EVs, we chose to focus on miR-423-5p for subsequent studies.

**Figure 3:**
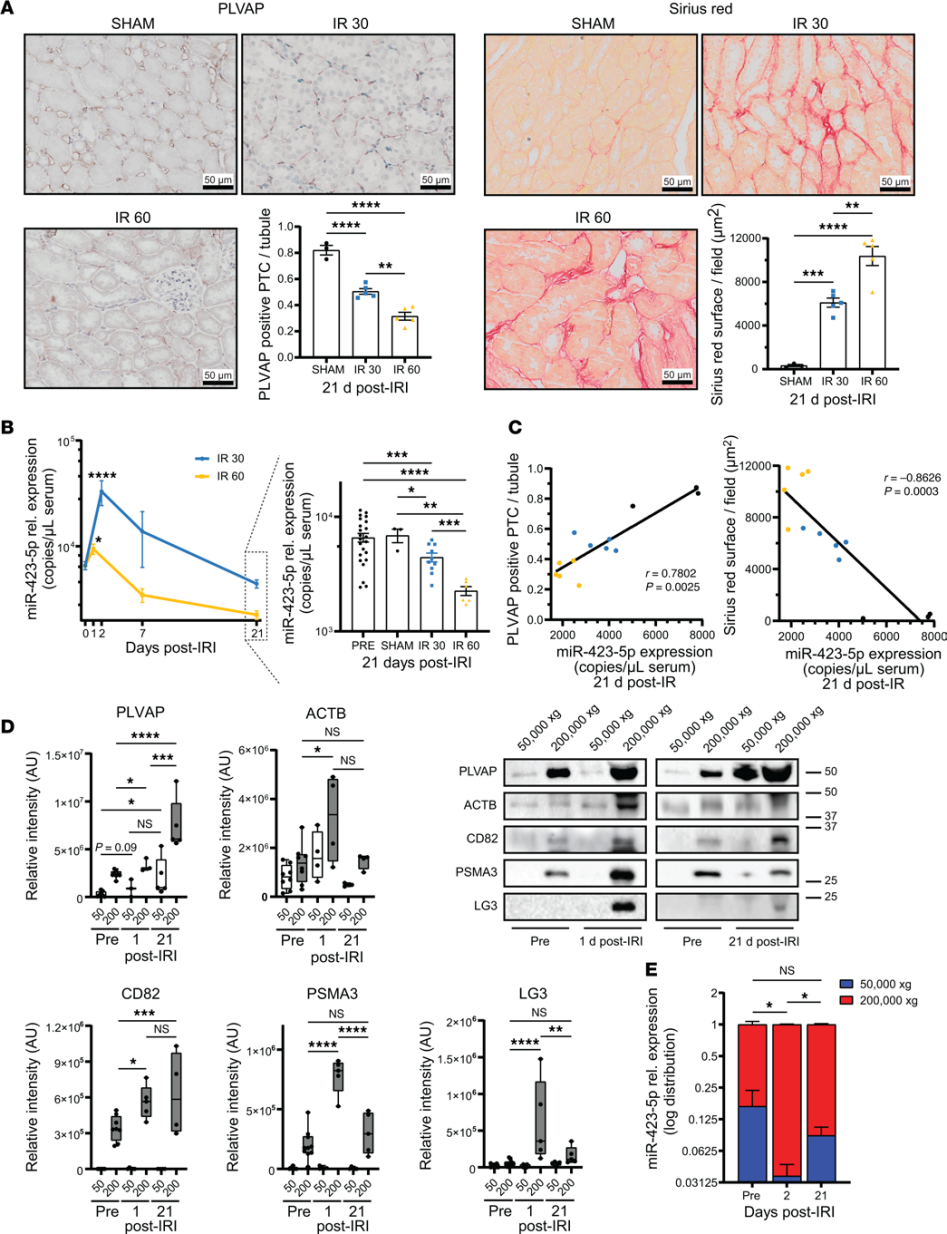
At a distance from IRI, lower miR-423-5p serum levels correlate with more severe microvascular rarefaction and renal fibrosis. (**A**) PLVAP IHC (left) and Sirius red staining (right) in corticomedullary junction in 21 days sham mice or 21 days after 30-minute and 60-minute IRI. Quantification of PLVAP^+^ PTC (left) and Sirius red^+^ area within PTC (right) in corticomedullary junction in 21 days sham mice or 21 days after 30-minute and 60-minute IRI; *n* = 3–5. (**B**) miR-423-5p serum levels at baseline or 1, 2, 7, and 21 days after 30-minute and 60-minute IRI or in 21-day sham mice. Expression of miR-423-5p measured by qPCR and presented as relative copies of miR-423-5p per μL of total serum ± SEM after normalization with cel-miR-39; *n* = 3–23 (Supplemental Figure 3E). (**C**) miR-423-5p serum levels at 21 days after sham surgery (black) or 30-minute (blue) and 60-minute (yellow) IRI correlated with microvascular density (***ρ*** = 0.7802; *P* = 0.0025) and inversely correlated with collagen deposition (***ρ*** = –0.8626; *P* = 0.0003). (**D**) Immunoblots and quantification of different protein markers in large (50,000*g*) and small (200,000*g*) extracellular vesicles (EVs) from mouse serum at baseline and 1 and 21 days after IRI. PLVAP for endothelial-derived EVs, PSMA3, and LG3 for apoptotic exosome-like vesicles, ACTB for EV marker, and CD82 for exosome marker; *n* = 3–10. (**E**) Distribution of miR-423-5p expression in large (50,000*g*) and small EVs (200,000*g*) from the serum of mice at baseline and 2 and 21 days after 30 minutes of IRI; *n* = 5 for each fraction. Data are shown as mean ± SEM. *P* values obtained by 1-way ANOVA and the Bonferroni post hoc test (*P < 0.05, **P < 0.01, ***P < 0.001, ****P < 0.0001). Scale bars: 50 µm.

We next used both mild and severe forms of renal IRI with 30 and 60 minutes of renal artery clamping, respectively, to evaluate the levels of miR-423-5p at 21 days after renal IRI, as compared with those of sham-treated mice. Mice exposed to severe ischemia showed higher BUN levels than mice exposed to mild ischemia at every time point up to 21 days after IRI (Supplemental Figure 3A). Microvascular rarefaction and fibrosis, as evaluated by immunostaining for PLVAP and Sirius red staining, respectively, were increased in mice with IRI compared with sham-treated mice and were higher in mice with 60 minutes of renal artery clamping (Figure 3A). We then assessed whole-serum levels of miR-423-5p in mice exposed to mild or severe IRI. In both groups, increased levels of miR-423-5p were observed in the first 2 days after renal IRI, followed by a progressive decline until 21 days. While the peak level of miR-423-5p occurred earlier in the severe form of IRI, it was lower compared with the mild group (Figure 3B and Supplemental Figure 3B). This suggested the possibility that nonapoptotic forms of cell death could be activated in the severe group. Analysis of apoptosis and necroptosis levels in PTCs on days 1 and 2 after IRI revealed stronger activation of caspase-3 in the severe group on day 1 and increased activation of phosphorylated receptor interacting serine/threonine kinase 3 (RIPK3), a marker of necroptosis, at both time points (Supplemental Figure 3, C and D). This reorientation of PTC cell death toward necroptosis, a form of cell death not associated with ApoExo release (20), could explain the earlier but lower peak of miR-423-5p expression in mice with severe IRI. In both IRI groups, miR-423-5p levels at 21 days were significantly lower than sham-treated mice at 21 days and mice at baseline (Figure 3B). They were also significantly lower in mice with severe IRI than in mice with mild IRI. miR-423-5p serum levels at 21 days after IRI strongly correlated with renal microvascular density and inversely correlated with collagen deposition (Figure 3C). We also evaluated whether contralateral nephrectomy was contributing to miR-423-5p levels in our model. To this end, contralateral nephrectomy was performed in absence of renal IRI. Nephrectomy alone did not induce tubular damage at 2 days and did not increase miR-423-5p levels, nor did it increase levels of ApoExos markers LG3 or 20S proteasome, suggesting that the peak in circulating miR-423-5p is a consequence of ischemia-reperfusion (Supplemental Figure 3, E–G). Collectively, and as opposed to findings in the early phase of AKI, these results show that, at a distance from IRI, higher miR-423-5p levels are associated with better microvascular density, reduced fibrosis, and better-preserved renal function.

These results prompted us to test the hypothesis that, at later time points after renal IRI, miR-423-5p serum levels are accounted for by caspase-3–independent EVs, such as microvesicles. To evaluate this possibility, we isolated small EVs (containing ApoExos and exosomes) and large EVs (containing apoptotic bodies and microvesicles) from the serum of mice exposed to IRI for 30 minutes, and we measured the expression of endothelial (PLVAP), ApoExo (LG3 and PSMA3), and general markers of EVs (CD82 and ACTB) as well as miR-423-5p levels. In the early phase of AKI, 20S proteasome, and LG3 levels significantly increased in fractions containing small EVs. The endothelial marker PLVAP was also significantly increased in the same fractions. In contrast, at 21 days after IRI, PLVAP expression was present both in small and large EVs fractions (Figure 3D). The distribution of miR-423-5p expression in the various EV fractions over time paralleled that of PLVAP (Figure 3E). To further confirm that serum levels of miR-423-5p at later time points are independent of caspase-3 activation, we assessed miR-423-5p serum levels in caspase-3^–/–^ mice exposed to IRI. We previously showed that caspase-3^–/–^ mice exposed to renal IRI show better preservation of microvascular integrity and better long-term kidney function (17, 27), as well as a higher survival rate following severe IRI (Supplemental Figure 4A). At 21 days after IRI, caspase-3^–/–^ mice exposed to renal IRI showed significantly higher miR-423-5p serum levels than WT controls as well as better preservation of PTC integrity, as evaluated with PLVAP staining, and reduced fibrosis (Supplemental Figure 4, B–D). To further test the possibility that miR-423-5p is released within small EVs through caspase-dependent pathways and in large EVs through caspase-independent pathways, we exposed HUVECs, either in normal culture conditions or serum-starved, to the pancaspase inhibitor zVAD-Fmk. miR-423-5p levels were significantly lower in small-EV fractions originating from zVAD-Fmk–treated serum-starved HUVECs (SS-HUVECs), whereas miR-423-5p levels were unaltered by zVAD-FMK in large EV fractions (Supplemental Figure 4E). Collectively, these results confirm that, in the early phase of AKI, miR-423-5p is cargoed in circulation by small EVs bearing endothelial markers, while in the long term after renal IRI, circulating miR-423-5p is cargoed by non-caspase-3–dependent EVs, with an increased contribution of large EVs.

### Lower miR-423-5p serum levels are associated with microvascular rarefaction in kidney transplant recipients with delayed graft function.

**Figure 4:**
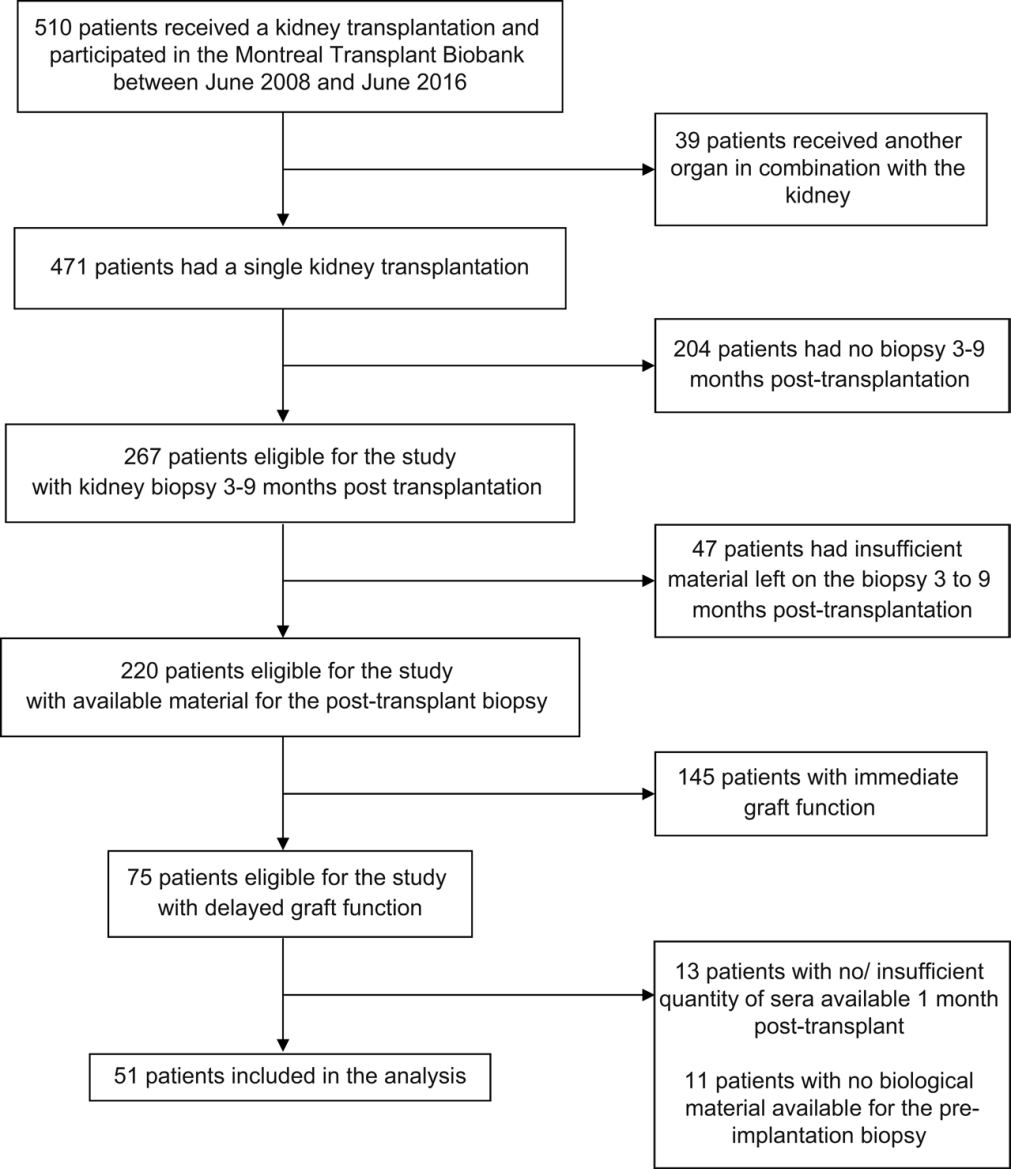
Patient flow chart.

**Figure 5:**
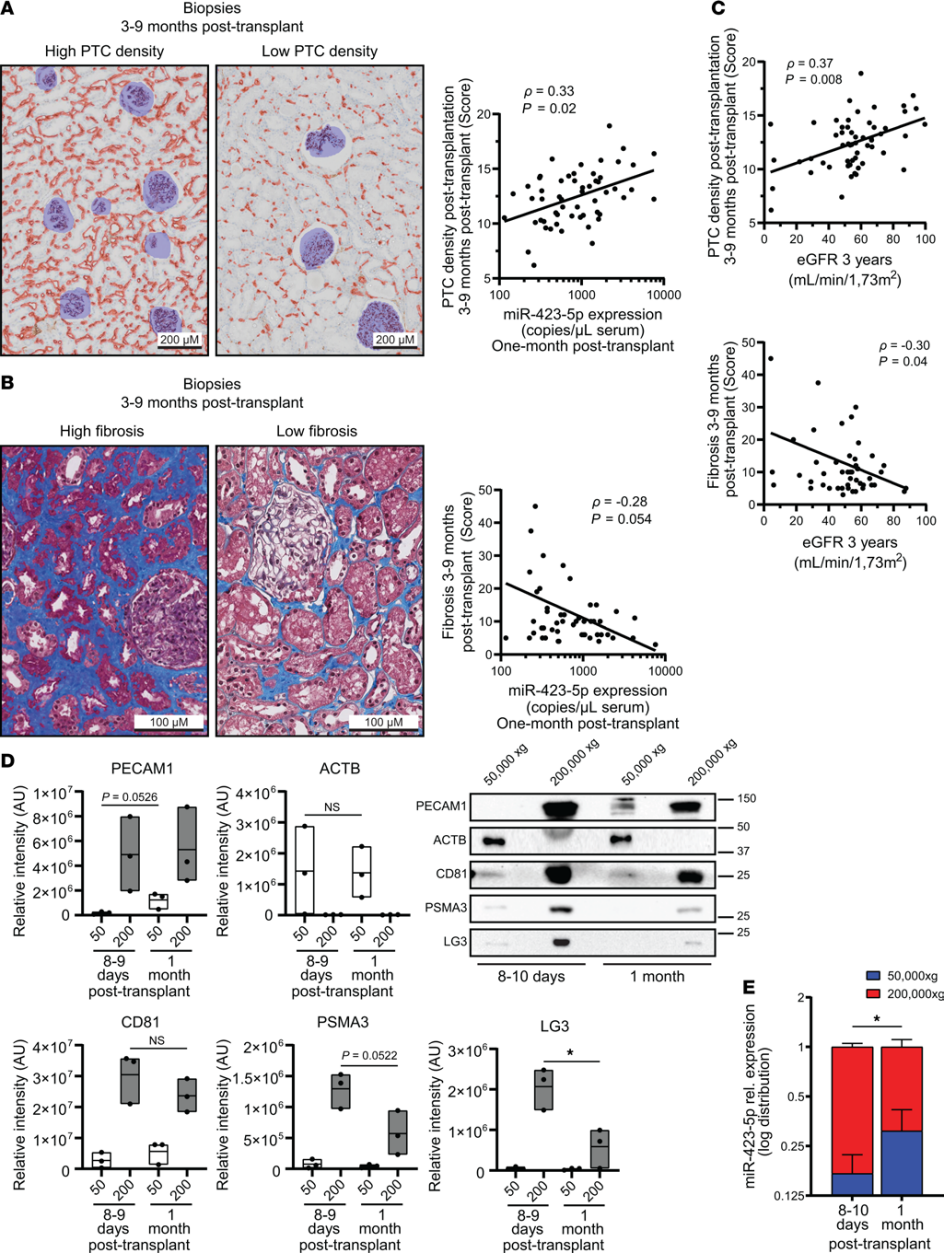
miR-423-5p serum levels predict microvascular rarefaction and fibrosis in human renal transplant patients. (**A**) Left: CD34 IHC in renal allograft biopsies performed 3–9 months after transplantation from patients with high or low peritubular capillary (PTC) densities. Middle: miR-423-5p serum levels in renal transplant patients with delayed graft function (DGF) at 1 month after transplantation correlate with the PTC density on the posttransplantation biopsy (***ρ*** = 0.33; *P* = 0.02); *n* = 51 (**B**) Left: Masson’s trichrome staining of renal allograft biopsy performed 3–9 months after transplantation from 2 different patients with high and low interstitial fibrosis. Right: miR-423-5p serum levels in patients with DGF at 1 month after transplantation inversely correlate with fibrosis based on the posttransplantation biopsy (***ρ*** = –0.28; *P* = 0.054); *n* = 51 (**C**) PTC density (***ρ*** = 0.37, *P* = 0.008) and fibrosis (*ρ* = –0.30, *P* = 0.04) on the 3- to 9-month posttransplantation biopsy are both associated with the estimated glomerular filtration rate (eGFR) at 3 years after transplantation in patients with DGF. (**A**–**C**) *P* and ***ρ*** values obtained by Pearson correlation coefficient; *n* = 51. (**D**) Immunoblots and quantification of different protein markers in large (50,000*g*) and small (200,000*g*) extracellular vesicles (EVs) from the serum of 3 randomly chosen patients with DGF at 8–9 days and 1 month after transplantation. PECAM1, endothelial marker; PSMA3 and LG3, apoptotic exosome-like vesicle markers; ACTB, general EV marker; CD81, exosome marker; *n* = 3. *P* values obtained by unpaired *t* tests (*P < 0.05). (**E**) Distribution of miR-423-5p expression in the fractions of large (50,000*g*) and small (200,000*g*) EVs from the serum of 6 patients with delayed DGF at 8–10 days and 1 month after transplantation; *n* = 6. *P* value obtained by paired *t* test (*P < 0.05). Scale bars: 200 µm.

To assess whether these observations hold true in humans, we first confirmed that miR-423-5p, let-7b-5p, and let-7c-5p were all detectable in sera from renal transplant recipients 1 month after transplantation. In humans, as in mice, total serum levels of miR-423-5p, let-7b-5p, and let-7c-5p were highly correlated with one another (Supplemental Figure 5A). We then compared the 3 miRNAs levels in total serum and pooled fractions of EVs purified from human serum. Similar levels of miR-423-5p were found in whole serum and pooled EV fractions, whereas levels of let-7b-5p and let-7c-5p in pooled vesicle fractions were significantly lower than those measured in total serum (Supplemental Figure 5B). These results suggest that the vast majority miR-423-5p found in circulation stems from circulating EVs, whereas circulating let-7b-5p and let-7c-5p levels are found both in EVs and in vesicle-free form. Based on these results, we chose to focus on miR-423-5p for subsequent studies, as its association with circulating vesicles would increase its stability and its potential use as a biomarker of microvascular injury. We then evaluated in patients with delayed graft function whether miR-423-5p serum levels measured 1 month after transplantation were predictive of microvascular rarefaction on the protocol biopsy performed between 3 and 9 months after transplantation. These time points were chosen based on availability of serum samples and to parallel those measured at 21 days after IRI in mice, reflecting a transition toward the chronic phase of AKI. After applying the exclusion criteria, 51 patients with delayed graft function were included in the analytical cohort (Figure 4). The main patient characteristics are presented in Table 1. The distribution of serum miR-423-5p was skewed to the right with a median of 590 copies/μL (interquartile range [IQR] 328–1,321) (Supplemental Figure 5C), and miR-423-5p levels were lower when thymoglobulin was used as induction immunosuppression (*P* = 0.03), and there was a trend for lower levels in female recipients (*P* = 0.07). We observed a correlation between miR-423-5p serum levels measured 1 month after transplantation and PTC density on the posttransplantation biopsy (*ρ* = 0.33, *P* = 0.02) (Figure 5A). We also observed a trend for a negative correlation between miR-423-5p one-month serum levels and fibrosis on the posttransplantation biopsy (*ρ* = –0.28, *P* = 0.054) (Figure 5B). As expected, both the PTC density (*ρ* = 0.37, *P* = 0.008) and fibrosis (*ρ* = –0.30, *P* = 0.04) on the 3- to 9-month posttransplantation biopsy were associated with the estimated glomerular filtration rate (eGFR) at 3 years after transplantation (Figure 5C).

In multivariable analyses (Table 2), we found that higher miR-423-5p 1 month after transplantation were associated with a higher PTC density on the posttransplantation biopsy (adjusted difference [AD]: +0.9% per 1 natural log higher in miR-423-5p levels [95% CI, 0.4, 1.4], *P* = 0.002) in a model that was adjusted for recipient sex, use of hypothermic pump during organ transportation, use of statins at transplant and PTC density on the pretransplantation biopsy. The univariable analysis results are presented in Supplemental Table 2A, while the initial multivariable model is presented in Supplemental Table 2B. We also found that higher miR-423-5p levels 1 month after transplantation were associated with less fibrosis on the posttransplantation biopsy (AD: –4.6% for a 1 natural log higher in miR-423-5p levels [95% CI, –8.2, –0.9], *P* = 0.02) (Table 3). Fibrosis on the posttransplantation biopsy was also associated with donor age (AD: +3.0% per 10 years higher [95% CI, 0.9, 5.0]) and with the occurrence of rejection before or on the 3–9 posttransplant biopsy (AD: +7.3 [95% CI, 0.5, 14.1]). The univariable analysis results are presented in Supplemental Table 3A, while the initial multivariable model is presented in Supplemental Table 3B. Altogether, these results suggest that, in humans as in mice, lower circulating miR-423-5p levels at a distance from IRI-induced AKI are associated with low PTC density and high renal fibrosis, both of which are associated with lower renal function in the long term.

We then evaluated, in a subset of patients for whom serial serum samples were available after transplantation, whether different types of EVs contribute to miR-423-5p serum levels over time after transplantation. In the early postoperative period (8–10 days after transplantation), the majority of the miR-423-5p signal was recovered in fractions of small EVs positive for the endothelial marker PECAM1 and for ApoExo markers (20S proteasome α3 and LG3). At 1 month after transplantation, there was an increasing contribution from fractions containing large EVs and showing increased positivity for PECAM1, in the absence of 20S proteasome and LG3 reactivity (Figure 5, D and E, and Supplemental Figure 5D). Collectively, these findings demonstrate that, in humans as in mice, the early increase in miR-423-5p levels after renal IRI is associated with the release of ApoExos bearing endothelial markers, while in the long term, circulating miR-423-5p levels are increasingly accounted for by endothelial-derived large EVs and reflect PTC density.

### miR-423-5p attenuates microvascular injury and prevents renal fibrogenesis after AKI.

**Figure 6:**
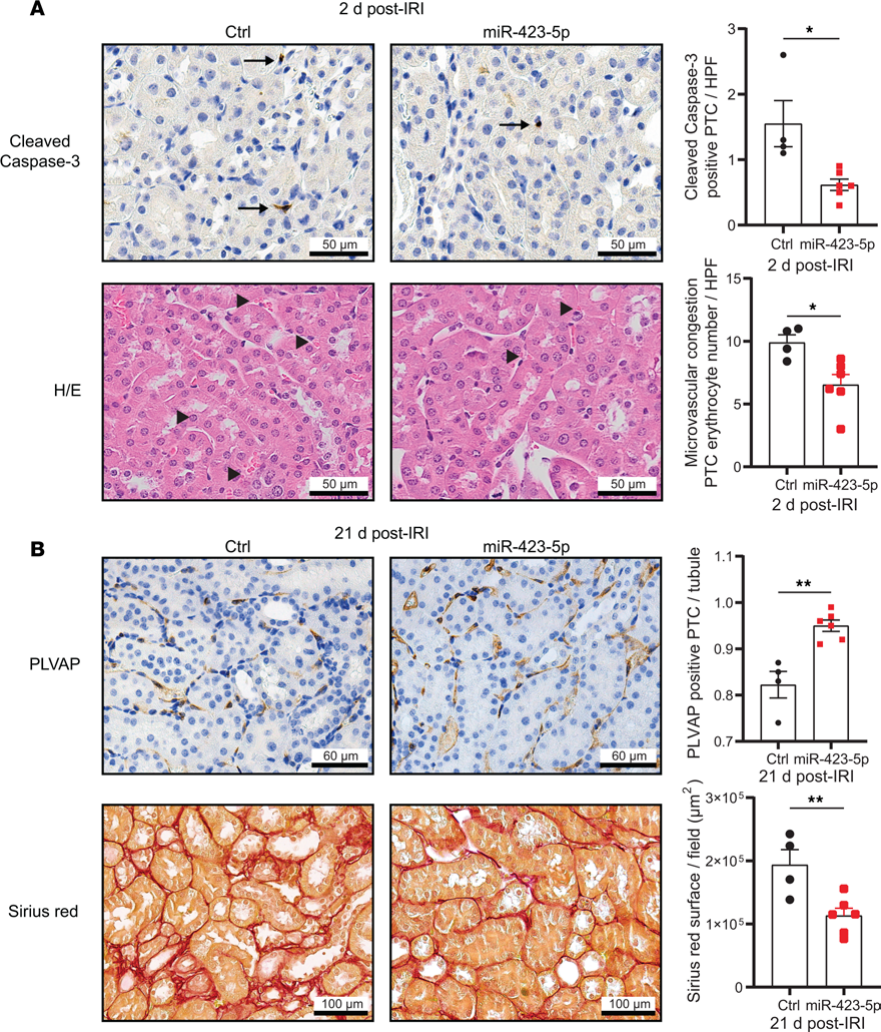
miR-423-5p attenuates microvascular injury and fibrogenesis after acute kidney injury. Mice were treated by renal subcapsular injection with miR-423-5p or with a scrambled miR mimic control (Ctrl) following 30 minutes of renal artery IRI. (**A**) Left: Representative images of cleaved caspase-3 IHC and H&E-stained renal sections 2 days after IRI. Right: Quantification of cleaved caspase-3^+^ peritubular capillaries (PTC) and erythrocytes in PTCs in murine renal sections (corticomedullary junction) 2 days after IRI per high-power field (HPF); *n* = 4–6. Arrow, cleaved caspase-3^+^ PTC; arrowhead, erythrocytes in PTC. (**B**) Left: Representative images of PLVAP immunohistochemistry and Sirius red staining within peritubular capillaries (PTCs) in renal sections (corticomedullary junction) 21 days after IRI. Right: Quantification of PLVAP^+^ PTCs per tubule and Sirius red^+^ area (μm^2^) within PTCs in murine renal sections (corticomedullary junction) at 21 days after IRI; *n* = 4–6. *P* values were obtained by unpaired *t* test (*P < 0.05, **P < 0.01) between the miR-423-5p and Ctrl groups. Scale bars:50 μm (**A**), PLVAP – 60 μm and Sirirus red – 100 μm (**B**).

The positive correlation we observed between miR-423-5p serum levels assessed at a distance from renal IRI and the preservation of microvascular integrity led us to explore the hypothesis that miR-423-5p actively contributes to microvascular homeostasis and repair, as suggested by the GO term enrichment analysis (Figure 1C). Using renal subcapsular injections of miR-423-5p or control mimic miRNA at the time of renal IRI, we investigated whether enhancing miR-423-5p expression prevents long-term microvascular rarefaction and renal fibrosis. We confirmed that miR-423-5p injection leads to significant renal overexpression throughout the entire kidney 2 days after IRI. A significant increase in miR-423-5p levels was also observed in 3 kidney segments (Q2–Q4), with an upward trend noted in one of the quarters farthest from the injection site (Supplemental Figure 6A). Thus, subcapsular injection facilitates the delivery of miR-423-5p beneath the renal capsule, promoting the diffusion of the miRNA throughout the kidney, in contrast to a localized injection. While BUN levels or IRI-induced tubular damage in the short term were not modulated (Supplemental Figure 6, B and C), caspase-3 activation and rouleaux formation in PTC were significantly attenuated by subcapsular injection of miR-423-5p (Figure 6A). miR-423-5p–injected mice showed reduced PTC rarefaction and decreased fibrosis 21 days after IRI compared with mimic miRNA-injected controls (Figure 6B).

### miR-423-5p increases VEGFA levels and promotes endothelial migration and angiogenesis.

**Figure 7:**
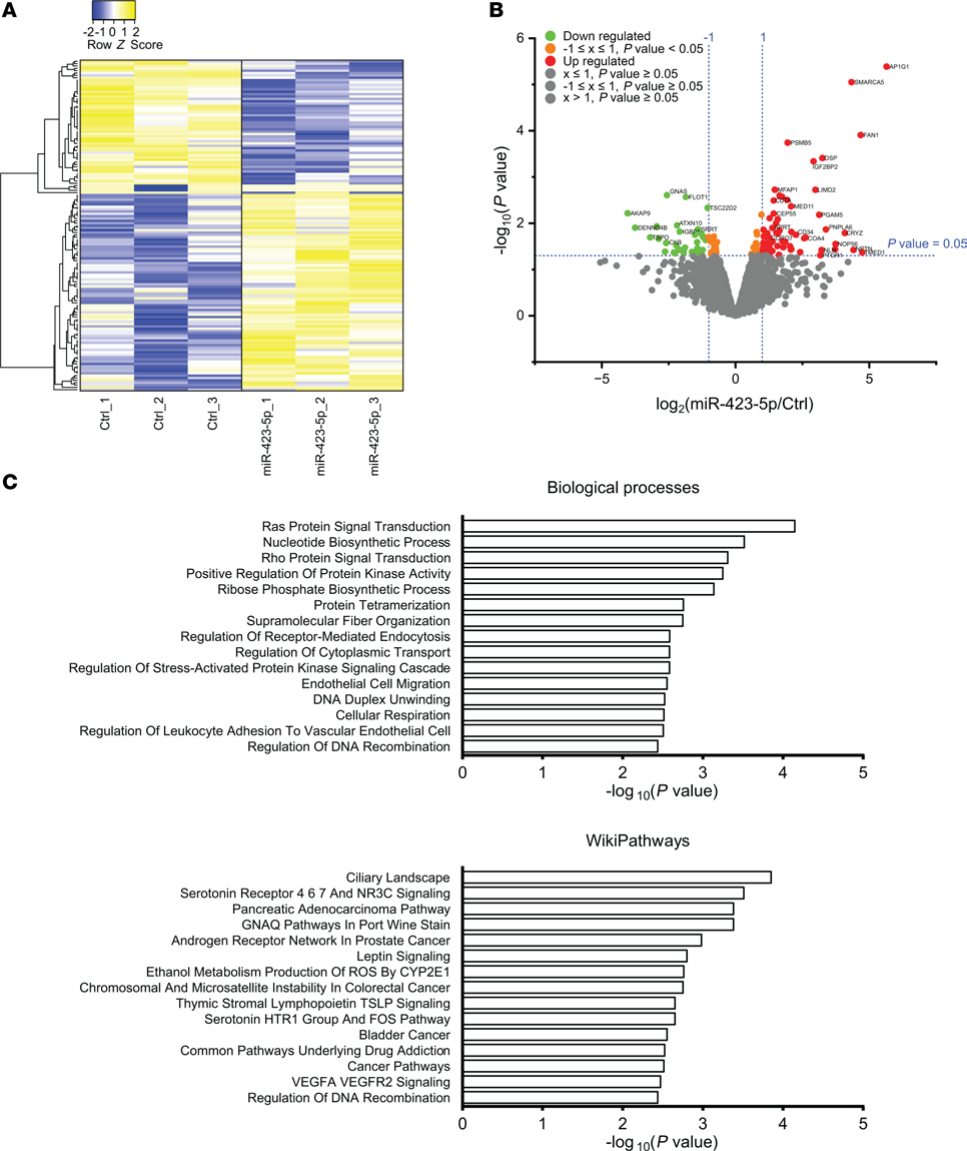
Expression of miR-423-5p drives a distinct protein signature in endothelial cells. (**A**) Heatmap representation of the differentially expressed proteins in endothelial cells transfected with scrambled miR mimic control (Ctrl) or miR-423-5p (10 nM). *n* = 3. (**B**) Volcano plot of quantitative proteomics data from endothelial cells transfected with either Ctrl or miR-423-5p mimics. The plot displays the significantly differentially expressed proteins identified through proteomics analysis. Proteins are ranked in the volcano plot according to their statistical *P* value (*y* axis) as –log_10_ and their relative abundance ratio (log_2_) between miR-423-5p and control samples (*x* axis). The cutoffs for significant changes are a fold change (FC) of ± 1 and *P* < 0.05. Red spots represent the upregulated proteins in miR-423-5p–transfected cells; green spots show the downregulated proteins in miR-423-5p–transfected cells; orange spots indicate the up- or downregulated proteins with –1 ≤ FC ≤ 1, *P* < 0.05; and gray spots show the unregulated proteins between both groups. (**C**) Enrichment analyses for biological processes and WikiPathways were conducted for proteins that were upregulated and downregulated in endothelial cells transfected with the miR-423-5p mimic compared with the control.

**Figure 8:**
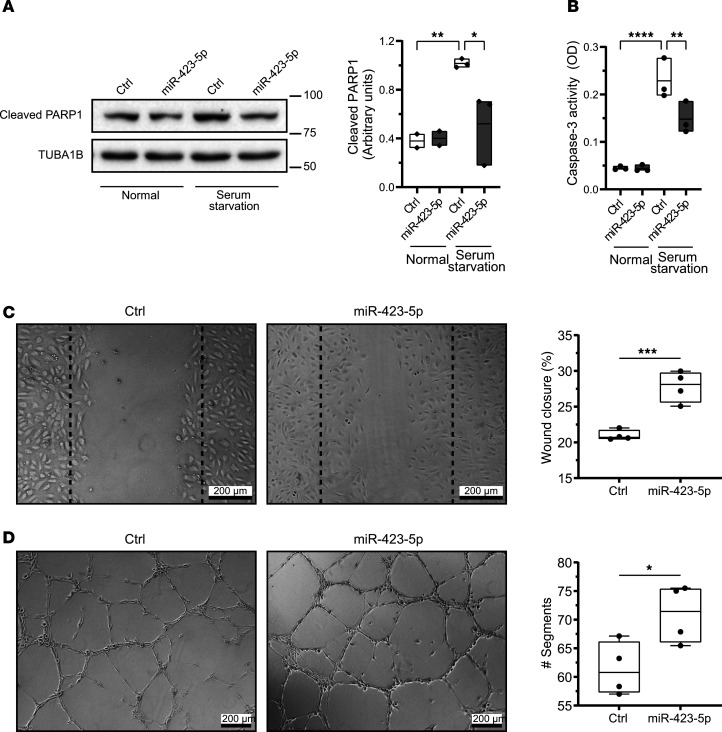
miR-423-5p protects endothelial cells from apoptosis and promotes endothelial migration and angiogenesis. (**A**) Immunoblot and densitometric analysis of cleaved PARP1 in endothelial cells transfected with scrambled miR mimic control (Ctrl) or miR-423-5p mimics (10 nM) and exposed to normal medium or serum starvation for 4 hours. TUBA1B was used as a loading control; *n* = 2–3. Scale bar: 200 µm (**B**) Quantification of caspase-3 activity in endothelial cells transfected with Ctrl or miR-423-5p mimics (10 nM) exposed to N or SS for 4 hours; *n* = 3. Scale bar: 100 µm (**C**) Endothelial cells transfected with scrambled miR mimic control (Ctrl) or miR-423-5p mimics (10 nM) were mechanically injured, and wound closure was followed over a 6-hour period. The wound healing assay results are expressed as the percentage of wound closure ± SEM; *n* = 4 for each condition. Representative pictures at 6 hours after injury are presented. (**D**) Endothelial cells were transfected with scrambled miR mimic control (Ctrl) or miR-423-5p mimics (10 nM), and capillary-like structures were quantified after 6 hours on Matrigel. Angiogenic activity was assessed by quantifying the number of segments per field ± SEM; *n* = 4 for each condition. Representative images of tubule formation are presented in the right panel. *P* values were obtained by 1-way ANOVA and the Bonferroni post hoc test (*P < 0.05, **P < 0.01, ***P < 0.001, ****P < 0.0001).

**Figure 9:**
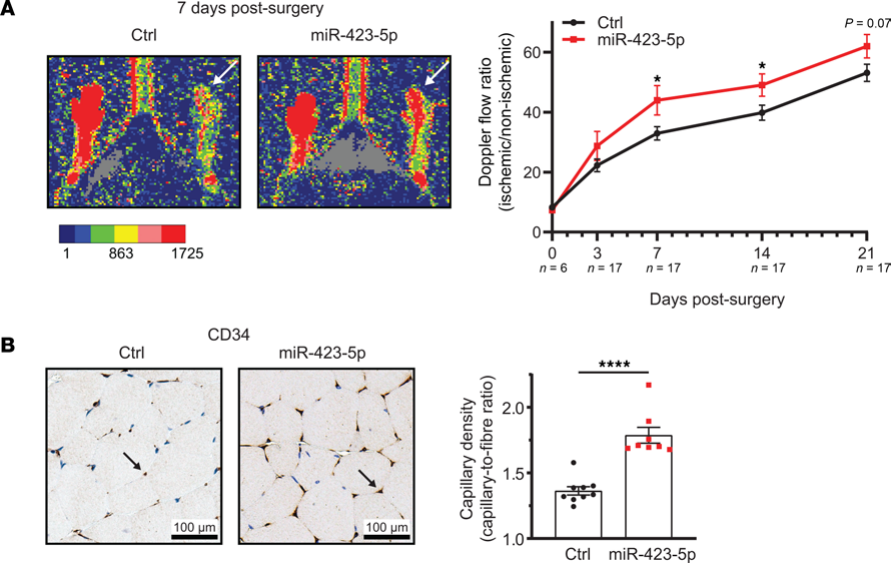
miR-423-5p increases neovascularization after hindlimb ischemia. (**A**) Representative results at 7 days after surgery and quantification by laser Doppler imaging of blood flow in the hindlimb of mice after femoral arteriectomy in mice treated with miR-423-5p or with a scrambled miR mimic control; *n* = 6–17 per time point. Arrow: ischemic hindlimbs. (**B**) Representative images of CD34 IHC and quantification of the capillary/fiber ratio in ischemic muscles of different groups at 21 days after surgery; *n* = 8–9 for each group. Arrow: positive CD34 staining in capillaries. *P* values were obtained by unpaired *t* test (*P < 0.05, ****P < 0.0001) between the miR-423-5p and Ctrl groups. Scale bars: 100 µm.

We then explored the potential molecular mechanisms supporting the renoprotective effect of miR-423-5p. To this end, we overexpressed miR-423-5p in ECs in vitro (Supplemental Figure 7A) and conducted a proteomic analysis. Cells transfected with miR-423-5p exhibited a distinct protein signature compared with those transfected with control miRNA. Proteomic analysis revealed that the overexpression of miR-423-5p led to an increase in the expression of 84 proteins and a decrease in 56 proteins (Figure 7, A and B). Enrichment analyses for biological processes and WikiPathways indicated several pathways associated with cell survival and proangiogenic mechanisms contributing to vascular integrity (Figure 7C). Overexpression of miR-423-5p in ECs in vitro also led to significant increases in *HIF1A* and *VEGFA* mRNA expression levels (Supplemental Figure 7B). Consequently, we exposed transfected ECs to serum-free medium, a well-known proapoptotic stimulus. We found reduced apoptosis levels, as evaluated by the cleavage of poly(ADP-ribose) polymerase 1 (PARP1) and caspase-3/7 activity, in ECs overexpressing miR-423-5p compared with cells overexpressing scrambled miRNA (Figure 8, A and B). Given that endothelial migration and angiogenesis are key to microvascular repair after IRI, we also investigated whether inducing transient overexpression of miR-423-5p in ECs modulates migration and angiogenesis. ECs overexpressing miR-423-5p exhibited significantly increased wound closure compared with control cells, along with enhanced angiogenesis (Figure 8, C and D, and Supplemental Figure 7C). We then tested the effect of miR-423-5p injections on angiogenesis in vivo in a model of femoral arteriectomy in mice where hindlimb reperfusion depends entirely on the development of new blood vessels (28). We validated that intramuscular injection of miR-423-5p induced significant overexpression of the miRNA throughout the entire muscle 3 days after surgery (Supplemental Figure 8). Mice receiving intramuscular miR-423-5p injections at the time of femoral arteriectomy showed increased Doppler flow rate recovery at days 7 and 14 as compared with controls injected with miRNA mimic negative control. The number of CD34^+^ capillaries was also significantly increased 3 weeks post-femoral artery ligation in mice injected with miR-423-5p when compared with control miRNAs (Figure 9).

**Figure 10:**
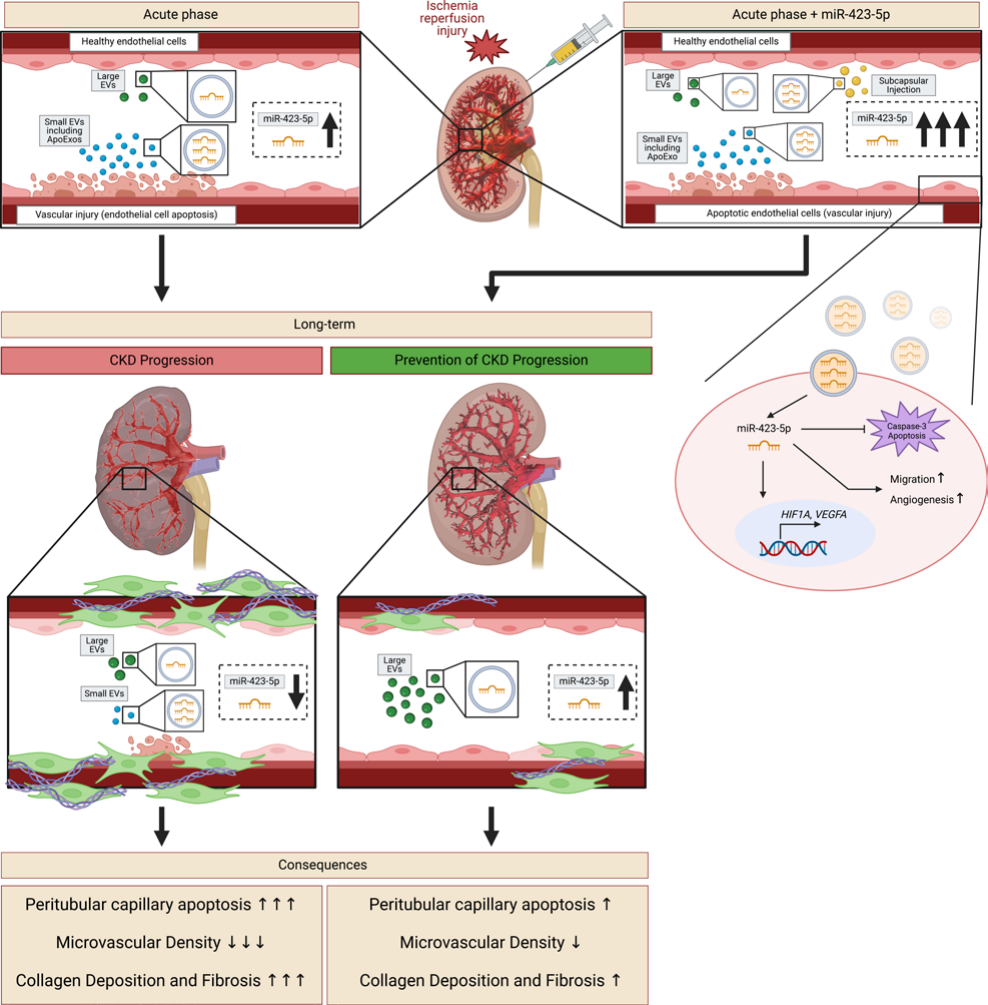
miR-423-5p controls microvascular homeostasis after renal IRI. Schematic diagram depicting the role of miR-423-5p in fostering microvascular repair and homeostasis. ApoExos, apoptotic exosome-like vesicles; EVs, extracellular vesicles; CKD, chronic kidney disease. The schematic was created with BioRender.com.

Altogether, these findings identify a key role for miR-423-5p in controlling renal microvascular homeostasis and repair. The enhanced release of miR-423-5p cargoed by endothelial ApoExos early after renal IRI is likely aimed at protecting the microvasculature during periods of acute stress. In the long term, however, major loss of PTC, such as the one observed after severe renal IRI, would limit the capacity to release large endothelial EVs expressing miR-423-5p, therefore hampering completion of microvascular repair. Increasing miR-423-5p levels through exogenous injections highlights potentially new avenues for preventing renal microvascular loss as well as fibrosis and progressive renal dysfunction (Figure 10).

## Discussion

The classification of the growing types of EVs is evolving rapidly, and mounting data suggest that environmental stressors influence the biogenesis of EVs and their effect on the local microenvironment. The present work adds further support to that notion. Using in vitro, in vivo, and within-human experimental strategies, we identified a pattern of miRNAs, specific to small EVs released by ECs under proapoptotic conditions. This miRNA pattern differs from those recovered from parental cells either in normal or proapoptotic conditions and from classical apoptotic bodies. We could track this pattern both in animal models of renal IRI and in human renal transplant patients. Serum levels of these miRNAs, but not control miR, increased in the early days after renal IRI and decreased progressively on the long term. Collectively, these results and past work from our group demonstrate that stressed and dying ECs regulate biogenesis of different types of EVs bearing specific protein and nucleic acid markers, including let-7b-5p, let-7c-5p, and most importantly, miR-423-5p.

One surprising finding was the inverse association between miR-423-5p circulating levels and microvascular rarefaction at a distance from renal IRI, both in murine models and in human renal transplant patients. At late time points, circulating miR-423-5p was still bound to EVs but a greater proportion was recovered in large rather than small vesicle fractions. Endothelial markers showed a similar shift: in the early stage after renal IRI, circulating PLVAP in mice and PECAM1 in humans were recovered exclusively in the fractions containing small vesicles. On the long term, both small and large circulating EV fractions expressed endothelial markers and miR-423-5p. The specific nature of these large EVs and their cellular origin, either within the kidney or from potential progenitor cells bearing endothelial markers, will be the scope of future studies. However, these findings prompted us to consider the possibility that miRNAs released by ApoExo in the early phase of AKI and by large EVs in late stages could play a role in maintaining microvascular homeostasis. Our findings with miR-423-5p largely support this hypothesis. ECs transfected with miR-423-5p showed increased resistance to apoptosis, increased migratory capacity, increased *VEGFA* mRNA levels, and enhanced angiogenesis. Similarly, mice that received subcapsular miR-423-5p injection at the time of renal IRI showed reduced caspase-3 activation within PTCs, reduced microvascular congestion, decreased microvascular rarefaction, and lower levels of renal fibrosis. Fibrosis and lower microvascular density are associated with reduced long-term renal function in renal transplant patients (9, 10). Our findings suggest that healthy microvascular beds contribute to maintaining the circulating levels of miR-423-5p, which in turn preserves renal microvascular homeostasis. However, when severe injury induces major microvascular loss, such as that occurring after severe ischemia in mice or after delayed graft function in renal transplants, microvascular rarefaction impairs the production and release of miR-423-5p. In turn, lower miR-423-5p serum levels translate into greater microvascular fostering a vicious cycle that accelerates microvascular loss, therefore fueling the development of fibrosis and deterioration of renal function (Figure 10).

Although the presence of miR-423-5p in EVs of endothelial origin is demonstrated, miR-423-5p levels have been studied in a number of kidney diseases, including diabetic nephropathy (29), membranous glomerulopathy (30), AKI (31), and CKD (32) with seemingly contradictory findings. Some studies indicate that lower circulating miR-423-5p levels are associated with renal damage and diminished renal function (29, 32, 33), while others show an association between circulating miR-423-5p levels and the severity of renal injury (30, 34). The transient acute increase in miR-423-5p circulating levels we observed after renal IRI, and the inverse correlation between miR-423-5p serum levels and microvascular rarefaction and fibrosis in the chronic phase of AKI, likely explain these opposing results. In these various studies, high circulating miR-423-5p levels were predominantly measured during the acute clinical phase or intensive care unit (ICU) admission. Conversely, low levels of miR-423-5p expression were observed in patients with CKD and those with poor clinical prognosis.

We also identified let-7b-5p and let-7c-5p within small EVs released by apoptotic ECs in vitro. However, we found that, in mice and in humans, a large proportion of serum let-7b-5p and let-7c-5p are not associated with EVs, as both showed major degradation when sera were treated with RNase in the absence of Triton-X. This finding suggest that levels of both miRNAs in total unfractionated serum would not behave as ideal biomarkers of microvascular injury and that purification of serum EVs would be required. Nonetheless, these miRNAs could play functional roles, in association with miR-423-5p, for regulating microvascular repair after IRI. The let-7 miRNA family has been implicated in the control of pluripotency (35) and angiogenesis (36–39). Let-7 miRNAs are hypoxia responsive and play important roles in derepressing *VEGFA* transcription (40, 41). Let-7b-5p and let-7c-5p have also been associated with inhibition of endothelial-mesenchymal transition and reduced renal fibrosis (42). Future studies will evaluate mechanisms of potential cross-talk between miR-423-5p, let-7b-5p and let-7b-5c.

Our study has a number of limitations. While the upregulated miRNAs in ApoExos distinctly differed from all other groups, the RNA-Seq results were based on only 2 replicates. Nonetheless, qPCR validation studies completed on 8 distinct samples supported these findings. Although we have demonstrated that levels of miR-423-5p, let-7b-5p, and let-7c-5p increase after renal IRI in association with increased levels of endothelial markers such as PLVAP and PECAM1 and that these miRNAs are present in EVs derived from ECs, we cannot exclude the potential contribution of other circulating cells, such as platelets and leukocytes, to the production of EVs during and after IRI. Future studies are necessary to characterize the full array of cell types and mechanisms regulating miR-423-5p, let-7b-5p, and let-7c-5p serum levels. Moreover, due to the relatively small sample size of our cohort study, we could not demonstrate an association between miR-423-5p serum levels assessed 1 month after transplantation and the eGFR 3 years later. However, we found a significant association between miR-423-5p and PTC density based on the 3- to 9-month posttransplantation biopsy, which in turn was associated with the eGFR at 3 years.

In summary, our study provides insights into mechanisms controlling microvascular homeostasis and repair following renal IRI. We found that a specific set of miRNAs including miR-423-5p, let-7b-5p, and let-7c-5p are highly expressed in endothelial ApoExos released in circulation during the early phase of AKI after renal IRI. In the long term, caspase-3–independent large EVs produced at least in part by ECs contribute to circulating miR-423-5p levels, which are directly correlated with renal microvascular density and indirectly correlated with renal fibrosis (Figure 10). Restoring miR-423-5p levels through exogenous injections or localized treatment represents a promising strategy for preventing long-term microvascular rarefaction and fibrosis after renal IRI and ultimately preserving long-term renal function.

## Methods

### Sex as a biological variable.

Our study exclusively examined female mice because male animals had a higher mortality rate. It is unknown whether the findings are relevant for male mice.

### Cell culture and reagents.

HUVECs were purchased from Cell Applications, cultured in Medium 200 + LSGS (Thermo Fisher Scientific) on gelatin-coated surfaces and used at passage 4. ApoExos were produced as described previously (18, 19).

### Vesicle isolation.

ApoExos were isolated from conditioned media by sequential centrifugation (50,000*g* for 15 minutes followed by 200,000*g* for 18 hours) as previously described (18, 19), and further details are provided in Supplemental Methods.

### Small transcriptome RNA-Seq.

Total RNA was extracted using TRIzol Reagent (Invitrogen) according to the manufacturer’s protocol. RNA was then purified using the miRNeasy micro kit (QIAGEN) and submitted to on-column DNase I digestion using the RNase-free DNase set (QIAGEN) as recommended. Sample quality and quantity were determined on an Agilent 2100 Bioanalyzer using an RNA 6000 Pico kit (Agilent Technologies). Eight RNA-Seq libraries (2 biological replicates of HUVECs under normal conditions [N-HUVECs], SS-HUVECs, apoptotic bodies and ApoExos) were generated from 20 ng RNA using the CleanTag kit for small RNAs (TriLink BioTechnologies). Single-read (1 × 75 base pairs) sequencing was performed on an Illumina NextSeq 550 (50M reads per sample). Sequences were trimmed for sequencing adapters and mapped to the reference genome GRCh38 using bowtie 1.2.1, part of the miRDeep2 package, version 0.0.8. Following mapping, reads were then postprocessed to quantify known miRNAs from the miRBase database (version 21) as well as to discover novel recurrent miRNAs in the sample data. We defined a miRNA as enriched in ApoExos when it exhibited a FPKM > 6,000 and an expression fold change > 2 for both biological replicates, when compared with apoptotic bodies as well as with serum-starved and normal ECs. An overrepresentation analysis of biological processes was performed using GO enrichment analysis coupled with Reduced + Visualized GO (Revigo) software.

### qPCR.

The expression levels of mRNA and miRNA in vesicles, serum, and cells were determined using qPCR. Total RNA was isolated from cells or EVs using the miRNeasy mini kit and 100 μL of patient serum or 20 μL of mouse serum using the miRNeasy serum/plasma kit according to the manufacturer’s protocol (QIAGEN). miRNAs were quantified using the Qubit miRNA Assay Kit (Invitrogen). Total RNA was quantified using a DS-11 Series Spectrophotometer/Fluorometer (DeNovix). Then, 0.5 ng of miRNA was reverse transcribed to cDNA and preamplified with a TaqMan Advanced miRNA cDNA Synthesis Kit (Applied Biosystems) according to the manufacturer’s protocols. One microgram of total RNA was treated with RNase free-DNase I (Invitrogen) and reverse transcribed to cDNA with iScript Reverse Transcription Supermix (Bio-Rad) according to the manufacturer’s instructions. qPCR amplification of miRNAs was performed using the TaqMan Advanced miRNA Assay (Applied Biosystems) on the QuantStudio 6 Real-Time PCR System (Thermo Fisher Scientific). TaqMan Advanced miRNA assays (miR-423-5p: 478090_mir, let-7b-5p: 478576_mir, let-7c-5p: 478577_mir, miR-361-5p: mmu481127_mir and cel-miR-39-3p: 478293_mir) were used to determine the expression of selected miRNAs following the manufacturer’s instructions. Each target was measured in triplicate and normalized to the exogenous level of cel-miR-39-5p. The TATAA Interplate Calibrator (TATAA Biocenter AA) was used to compensate for the variation between qPCR runs. For qPCR amplification of mRNA, 5 ng of cDNA was amplified with *HIF1A* (Hs00153153_m1), *VEGFA* (Hs00900055_m1), or *HPRT1* (Hs03929098_m1) TaqMan probes (Thermo Fisher) using TaqMan Fast Advanced Master Mix (Thermo Fisher Scientific). The reaction was performed in a total volume of 15 μL, and the reaction conditions were as follows: denaturation at 95°C for 20 seconds, followed by 40 cycles of denaturation at 95°C for 1 second and annealing and extension at 60°C for 20 seconds. The fold change in *HIF1A* and *VEGFA* mRNA levels was calculated with the comparative Ct method and normalized to *HPRT1*.

### Mimic miR-423 transfection.

Cells were plated onto 6-well plates at 2,500 cells per cm^2^. After 72 hours, cells were transfected with miRIDIAN miRNA Mimic Transfection Control or miRIDIAN miRNA Human hsa-miR-423-5p mimic (Dharmacon) using magnet-assisted transfection (MATra) (IBA Lifesciences) according to the manufacturer’s instructions. Further details are provided in Supplemental Methods.

### Proteomic analysis.

Samples were reconstituted in 50 mM Ammonium Bicarbonate Urea 8M, vortexed, and further diluted to 50 mM ammonium bicarbonate urea 1M with 50mM ammonium bicarbonate solution with 10 mM TCEP [Tris(2-carboxyethyl)phosphine hydrochloride; Thermo Fisher Scientific], and vortexed for 1 hour at 37°C. Chloroacetamide (Sigma-Aldrich) was added for alkylation to a final concentration of 55 mM. Samples were vortexed for 1 hour at 37°C. One microgram of trypsin was added, and digestion was performed for 8 hours at 37°C. Samples were dried down and solubilized in 5% ACN- and 4% formic acid (FA). The samples were loaded on a 1.5 μL precolumn (Optimize Technologies). Peptides were separated on a home-made reversed-phase column (150 μm i.d. by 200 mm) with a 56-minute gradient from 10% to 30% ACN-0.2% FA and a 600 nL/min flow rate on an Easy nLC-1200 connected to an Exploris 480 (Thermo Fisher Scientific). Each full MS spectrum acquired at a resolution of 120,000 was followed by tandem mass spectrometry (MS/MS) spectra acquisition on the most abundant multiply charged precursor ions for 3 seconds. MS/MS experiments were performed using higher energy collision dissociation (HCD) at a collision energy of 34%. The data were processed using PEAKS X Pro (Bioinformatics Solutions) and a human Uniprot database. Mass tolerances on precursor and fragment ions were 10 ppm and 0.01 Da, respectively. Fixed modification was carbamidomethyl. Variable selected posttranslational modifications were acetylation, oxidation, deamidation, phosphorylation. The data were visualized with Scaffold 5.0 (protein threshold, 99%, with at least 2 peptides identified and a FDR of 1% for peptides).

### Wound healing assay, EC tube formation assay, and apoptosis level assessment.

These assays were performed as described previously (19, 43, 44), and details are provided Supplemental Methods.

### Caspase-3 activity assay.

Caspase-3 activity was evaluated using a colorimetric assay kit (ab39401, Abcam) according to the manufacturer’s instructions. The samples were read on a microplate reader reading absorbance at 405 nm.

### Proteasome activity assay.

Proteasome activity was assessed as described before (18, 21), and further details are provided in Supplemental Methods.

### Flow cytometric analyses of EVs.

Analyses were performed on a BD Canto II Special Order Research Product (BD Biosciences) equipped with a small particle option, as described previously (45, 46). Samples were labeled in a total reaction volume of 100 μL at 37°C for 60 minutes with the LWA300 probe (125 nM) (Hermen Overkleeft, Department of Bioorganic Synthesis, Leiden University, Leiden, Netherlands). Three microliters of annexin V APC was added for 15 minutes at room temperature. Then, the sample was diluted by adding 100 μL of labeling buffer prior to analysis by high-sensitivity flow cytometry. All samples were processed and analyzed by an investigator blinded to experimental conditions. Further details are provided in Supplemental Methods.

### Cell lysis, protein isolation, and immunoblotting.

Cell lysis, protein isolation, and immunoblotting were performed as described previously (18–21), and additional details are provided in Supplemental Methods. Antibodies against: PARP1 (9542; Cell Signaling Technology), TUBA1B (11224-1-AP; Proteintech), PLVAP (NB100-77668; Novus Biological), ACTB (a5441; Sigma-Aldrich), PECAM1 (AF3628-SP; R&D Systems), CD82 (ab66400; Abcam), CD81 (66866-1-IG; Thermo Fisher Scientific), SDCBP (SC-515538; Santa Cruz Biotechnology), PSMA3 (SC-67340, Santa Cruz Biotechnology; 11887-1-AP, Proteintech), HSPG2/LG3 (AF2364; R&D Systems or polyclonal antibody against recombinant LG3; MediMabs), and histone H3C1 (9715S; Cell Signaling Technology).

### Animal studies.

Adult female C57BL/6N (20–22 g; Charles River Laboratories) mice were housed in sterilized, ventilated cages in a specific pathogen–free animal facility under a standard 12-hour light/12-hour dark cycle and fed a normal diet ad libitum.

### Murine hindlimb ischemia model.

Unilateral hindlimb ischemia, a model of persistent vascular injury (47), was surgically induced after anesthesia with 2% isoflurane by femoral arteriectomy as described previously (18). Mice were injected intramuscularly with 5 mg/kg of miRIDIAN miRNA mmu-miR-423-5p mimic or miRIDIAN miRNA Mimic negative control #1 (Dharmacon) (48). Resuspended miRNAs were administered in a solution of Max suppressor RNA-LANCEr II (Bioo Scientific) according to the manufacturer’s instructions. Resuspended miRNAs (25 μL) were injected at 5 points (5 μL each) using a 33 gauge, small hub RN 1 inch needle with a Hamilton syringe. Hindlimb blood flow recovery was monitored with a laser Doppler perfusion imager (LDPI) system (Moor Instrument Ltd.) after anesthesia with a ketamine-dexmedetomidine solution (50 mg/kg and 0.5 mg/kg, IP). LDPI measurements were performed on days 3, 7, 14, and 21 after surgery, and then dexmedetomidine was antagonized with a solution of atipamezole (1 mg/kg, s.c.). Blood flow is expressed as the ratio of perfusion in the ischemic versus nonischemic hindlimb to account for variables.

### Renal IRI model.

Renal IRI by unilateral renal artery clamping plus contralateral nephrectomy was performed as described previously (17, 27). Detailed surgical procedures can be found in Supplemental Methods. Subcapsular injection of miRIDIAN miRNA mmu-miR-423-5p mimic or miRIDIAN miRNA Mimic negative control #1 (Dharmacon) was performed directly after reperfusion. Resuspended miRNAs (25 μg) were administered using in vivo-jetPEI reagent (N/P ratio: 8) according to the manufacturer’s instructions (Polyplus transfection). The right kidney was then exposed, and ligation of the ureter and renal blood vessels with a 4-0 suture was performed before right kidney nephrectomy. Mice that underwent a sham operation, defined as the same procedures described above but without performing a 30- or 60-minute IRI or contralateral nephrectomy, were included in the study; some mice underwent the same procedure as sham but with contralateral nephrectomy and are identified as sham + nephrectomy. Mice were euthanized at baseline or on days 1, 2, 7, or 21 after surgery, and the left kidney, serum, and urine were collected.

### Biochemical analysis of renal function.

BUN levels were measured using the Quantichrom urea assay kit (BioAssay Systems) according to the manufacturer’s instructions.

### Kidney processing, histological stains, and IHC.

Mice were sacrificed at different time points (baseline and days 1, 2, 7, and 21). Kidneys were collected and fixed in 10% formalin, embedded in paraffin, and subsequently cut into 4 μm slices. IHC staining was performed on paraffin-embedded tissue as described previously (17) using PLVAP (120501; BioLegend), cleaved caspase-3 (9661; Cell Signaling Technology) or phospho-RIPK3 (ab195117; Abcam) antibodies. Stained slides were scanned using an Olympus VS110 slide scanner, and randomly chosen fields were evaluated. Quantification of PLVAP and cleaved caspase-3 staining in PTCs was assessed by evaluating the ratio of positive PTCs/tubule in 5 high-power fields (200×) in the cortico-medullary junction. Caspase-3 activation in ECs of PTCs was evaluated using sections stained for cleaved caspase-3. To ensure accurate identification, we counted the total number of ECs displaying a strong cleaved caspase-3 signal based on specific localization criteria (present within a vascular structure lined by a cellular monolayer). This assessment was conducted on 2 consecutive sections to confirm that the observed cells were integral to the peritubular vessel wall rather than circulating cells. Rouleaux formation and Sirius red^+^ areas were assessed as described previously (17, 27), and additional details are provided in Supplemental Methods. Five randomly chosen high-power fields at the cortical-medullary junction (magnification, 200×) were assessed. All assessments were conducted by an independent investigator who was blinded to the experimental conditions, and the results were subsequently validated by a pathologist.

### Skeletal muscle capillary IHC.

Ischemic hindlimbs were harvested 21 days after surgery and fixed in 10% formalin. Transverse 3 mm–thick tissue sections of the hindlimbs were cut at the level of the gastrocnemius muscle and paraffin embedded. IHC staining of capillaries was performed using CD34 antibody (ab81289; Abcam). Slides were scanned using an Olympus VS110 slide scanner microscope. Nine randomly chosen high-power fields (magnification, 200×) were assessed. Capillaries and muscle fibers were counted at 200× magnification. The results are expressed as the ratio of capillaries to fibers per field. All assessments were done by 2 independent investigators blinded to experimental conditions.

### Participants, setting, and study design.

We performed a single-center, observational, retrospective cohort study of kidney transplant recipients who participated in the University of Montreal Kidney Transplant Biobank (Centre Hospitalier de l’Université de Montréal site). All patients who received a transplant between June 2008 and 2017 and experienced delayed graft function were eligible for inclusion if they had leftover material from a graft biopsy preimplantation and 3 to 9 months after transplantation as well as serum banked 1 month after transplantation. Delayed graft function was defined as the need for dialysis in the first week after transplant, failure of serum creatinine to decrease by more than 10% on 3 consecutive days after transplant, or serum creatinine > 250 μmol/L on day 5 after transplantation in the presence of scintigraphic evidence of acute tubular necrosis, a definition we have previously found to be associated with lower 1-year kidney graft function (16). Posttransplantation biopsy was performed for surveillance purposes or to investigate graft dysfunction. All preimplantation biopsies were wedge biopsies, and all posttransplantation samples originated from core biopsies. Recipients of nonkidney solid organ transplants were excluded. Patients were followed for 3 years after transplantation. We also used a subset of the cohort with early biological material available (*n* = 6) to compare the contribution of different EV fractions to miR-423-5p serum levels early (8–10 days) and late (1 month) after transplant.

### Measurements.

The PTC density on the posttransplantation biopsy was the primary outcome. PTC density was defined as the percentage of efficient cortical area occupied by PTC and was measured by quantifying CD34 mAb staining on IHC using VIS, an image analysis software, as described previously (10). The secondary outcomes were the presence of fibrosis on the posttransplantation biopsy and eGFR 1 and 3 years after transplantation. The percentage of fibrosis was assessed by 2 independent evaluators on Masson trichrome–stained slides. Any difference greater than 5% between the values reported by the 2 operators was resolved by consensus. All operators involved in scoring biopsies were blinded to miR-423-5p expression values. The operator measuring miR-423-5p expression was blinded to PTC density and fibrosis scores. We calculated the eGFR 1 and 3 years after transplantation with the 4-variable modification of diet in renal disease (MDRD) equation (49). Identification of other variables associated with PTC density and fibrosis is detailed in the Supplemental Methods.

### Statistics.

Continuous variables are reported as the mean ± SD when normally distributed or as the median ± interquartile range. Categorical variables are summarized as proportions. We used the χ^2^ test (or Fisher’s exact test when the expected number of events was less than 5 in a cell) to compare categorical variables and 2-tailed Student’s *t* test (or the Kruskal-Wallis test when not normally distributed) to compare continuous variables between various groups. We used the Pearson correlation coefficient (*ρ*) to assess correlations between continuous variables.

We fit multivariable linear regression models to determine whether miR-423-5p measured 1 month after transplantation was associated with (a) PTC density and (b) fibrosis on the 3- to 9-month posttransplantation biopsy. In the multivariable models, all variables associated with miR-423-5p or either (a) PTC density (Supplemental Table 2) or (b) fibrosis (Supplemental Table 3) on the 3- to 9-month biopsy with a *P* < 0.15 were included to control for potential confounders in the initial multivariable models. miR-423-5p levels were transformed in the natural log to improve the model fit given miR-423-5p’s skewed distribution. Given the limited sample size, we then simplified the initial multivariable models by removing independent variables that were not associated with the dependent ones (*P* ≥ 0.05), if their removal did not modify the multivariable β coefficient for miR-423-5p and PTC density or fibrosis by ≥ 10%. Biopsy size was adjusted for in the multivariable model for PTC density to account for technical measurement considerations. The normality assumption was verified by plotting the model residuals. All analyses were executed with SAS version 9.4.

All data are presented as the mean ± SEM of at least 3 independent experiments unless otherwise indicated. Data were compared using 2-tailed Student’s *t* test, 1-way ANOVA, or as stated otherwise in the legend with GraphPad Prism 5 software (GraphPad Software Inc.). A *P* value less than 0.05 was considered significant.

### Study approval.

All animal experiments and methods were performed following the relevant guidelines and regulations approved by the CRCHUM’s Comité Institutionnel de Protection des Animaux (CIPA), Montréal, Québec Canada. The cohort study was approved by the local ethics committee (Comité d’éthique de la recherche du CHUM, Montréal, Québec, Canada) (reference no. 16.204), and all patients provided informed written consent.

### Data availability.

Values for all data points in graphs are reported in the Supporting Data Values file. RNA-Seq data are available in the Gene Expression Omnibus archives under accession number GSE119108.

## Author contributions

FM, HC, and MJH conceived and designed the research program. FM, HK, AD, SL, AG, MHN, AKR, MD, IB, E Bonneil, and SD performed the experiments. FM, HK, AD, SL, AG, AKR, JT, E Bonneil, MD, and HC analyzed the data. FM, HK, AD, SL, JT, PT, MD, E Boilard, AR, HC, and MJH interpreted the results. FM prepared the figures. FM, HC, and MJH drafted the manuscript. FM, JT, PT, MD, E Boilard, AR, HC, and MJH edited and revised the manuscript. All the authors approved the final version of the manuscript.

## Supplementary Material

Supplemental data

Unedited blot and gel images

Supporting data values

## Figures and Tables

**Table 1:**
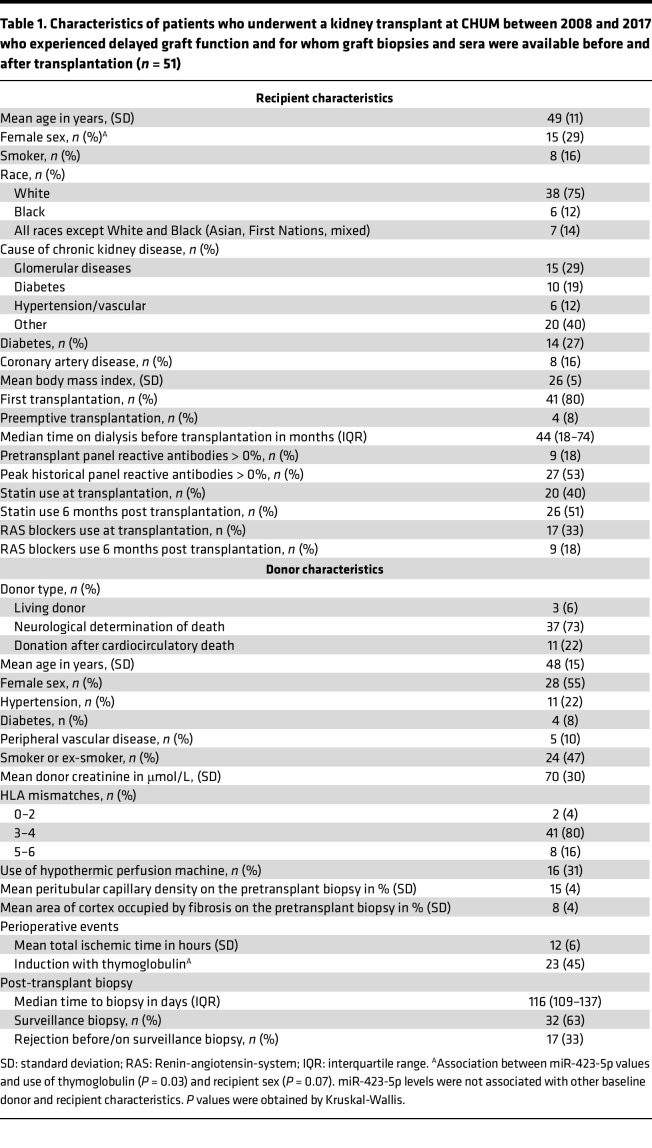
Characteristics of patients who underwent a kidney transplant at CHUM between 2008 and 2017 who experienced delayed graft function and for whom graft biopsies and sera were available before and after transplantation (*n* = 51)

**Table 2:**
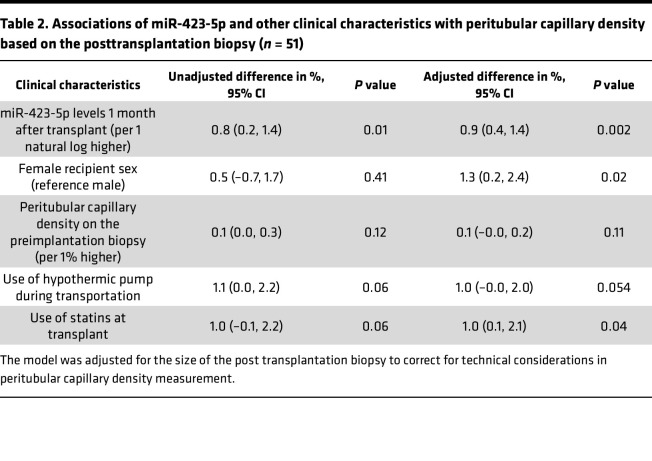
Associations of miR-423-5p and other clinical characteristics with peritubular capillary density based on the posttransplantation biopsy (*n* = 51)

**Table 3:**
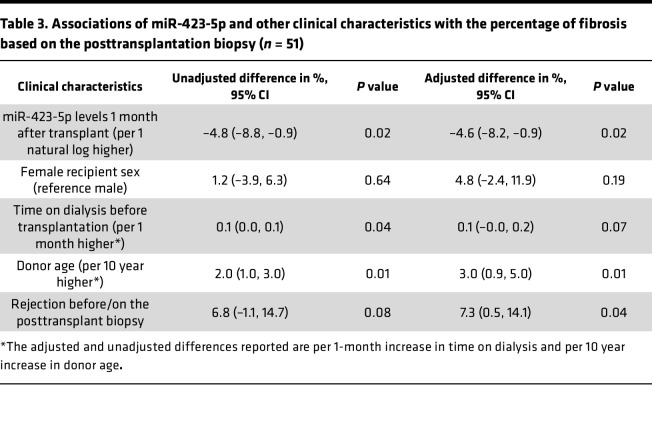
Associations of miR-423-5p and other clinical characteristics with the percentage of fibrosis based on the posttransplantation biopsy (*n* = 51)

## Version 1 05/22/2025

Electronic publication
