# Supplementary material for: Endothelial extracellular vesicle miR-423-5p regulates microvascular homeostasis and renal function after ischemia-reperfusion injury
Source: JCI Insight. 2025 May 22;10(10):e181937. doi: 10.1172/jci.insight.181937 (PMC12128966; doi:10.1172/jci.insight.181937)
Supplement: Supplemental data [file jciinsight-10-181937-s222.pdf]

Supplementary Materials for

Endothelial extracellular vesicle miR-423-5p regulates microvascular homeostasis and renal function after ischemia-reperfusion injury

Francis Migneault[1,2,3], Hyunyun Kim[1,2,4], Alice Doreille[1], Shanshan Lan[1,2,4], Alexis Gendron[1,4], Marie-Hélène Normand[1,2,3], Annie Karakeussian Rimbaud[1], Martin Dupont[1], Isabelle Bourdeau[1], Éric Bonneil[5], Julie Turgeon[1,2], Sylvie Dussault[1], Pierre Thibault[2,5,6], Mélanie Dieudé[1,2,3,7], Éric Boilard[2,8], Alain Rivard[1,4], Héloïse Cardinal[1,2,4], and Marie-Josée Hébert[1,2,4]

[1]Centre de Recherche, Centre Hospitalier de l'Université de Montréal (CRCHUM), Montréal, QC, Canada.

[2]Canadian Donation and Transplantation Research Program (CDTRP), AL, Canada.

[3]Département de Microbiologie, Infectiologie et Immunologie, Faculté de Médecine, Université de Montréal, Montréal, QC, Canada.

[4]Département de Médecine, Université de Montréal, Montréal, QC, Canada.

[5]Institute for Research in Immunology and Cancer, Montréal, QC, Canada.

[6]Department of Chemistry, Université de Montréal, Montréal, QC, Canada.

[7]Héma-Québec, Québec, QC, Canada.

[8]Centre de Recherche du, Centre Hospitalier Universitaire (CHU) de Québec-Université Laval, Département de Microbiologie et Immunologie Québec, QC, Canada.

Running title: **miR-423 -5p and renal ischemia reperfusion**

Corresponding author
Dr. Marie-Josée Hébert, marie-josee.hebert@umontreal.ca
CRCHUM, 900 St. Denis Street, Pavilion R, Room R12.412, Montreal, QC, Canada H2X 0A9. Telephone: (514) 890-8000 Extension 28479, Fax: (514) 412-7944.

DISCLOSURE

The authors of this manuscript have no conflicts of interest to disclose.

Supplemental Materials and Methods

Vesicle isolation. Briefly, conditioned serum-free media was centrifuged at 1200 x g for 15 min at 4 °C to remove dead cells and debris. The supernatant was exposed to centrifugation at 50,000 x g for 15 min at 4 °C to collect apoptotic bodies, followed by final centrifugation at 200,000 xg for 18 h at 4 °C to collect apoptotic exosome-like vesicles. Extracellular vesicles were resuspended in TRIzol Reagent (Life Technologies) to extract total RNA or D-PBS to assess proteasome activity.

Mimic miR-423 transfection. MATra-si Reagent was added to mimic miRNA to a concentration of 100 nM in Opti-MEM medium (Gibco) and incubated for 25 min at room temperature. The miRNA-bead mixture was added to the supernatant (Opti-MEM) of each well for a final concentration of 10 nM mimic miRNA. Then, the plate was placed on a magnet plate for 15 min and medium was changed after 30 min. After 48 h, the cells were processed according to the respective assays.

Wound healing assay. HUVECs were cultured on 12-well plates until confluence. The cells were mechanically injured using a P20/P200 pipette tip (three wounds per condition) as previously described (1). Wound closure was quantified with TScratch software (2) from the wound area measured after 6 h compared with the initial wound area for each wound.

Endothelial cell tube formation assay. Confluent HUVECs seeded onto 6-W plates were detached and reseeded at a cell density of 130 000 cells/well onto 24-W plates that had been precoated with growth factor-reduced Matrigel Matrix (Corning) and cultured at 37 °C for 6 h under their respective treatments. Each experiment was performed in duplicate for each condition. Capillary-like structures were captured (four fields per well) and quantified using the Angiogenesis analyzer for ImageJ (3).

Apoptosis level assessment. HUVECs were cultured in 6-well plates until they reached 90-95% confluence. Cells were rinsed twice with RPMI medium and then incubated for 4 h in either RPMI or complete M200 medium. After incubation, the cells were stained with Hoechst 33342 at a dilution of 1:10 000 (Molecular Probes) for 10 minutes. Just before analysis, propidium iodide was added to the culture medium to a final concentration of 5 µg/mL, with excitation set between λ = 360–425 nm. An investigator, who was blinded to the experimental conditions, assessed the proportions of normal, apoptotic, and necrotic adherent cells across eight random fields for each condition. Apoptotic cells were identified by their condensed nuclei (appearing bright blue) without any cell membrane permeability (indicated by red staining). The count of apoptotic cells was then divided by the total number of cells in each micrograph to calculate the percentage of apoptotic cells.

Proteasome activity assay. The proteasome activity assay was performed in white 96-well plates with clear bottoms using exosome-like vesicles purified from mouse serum (50 µg) and Proteasome-Glo Caspase-Like Cell-Based Assays (Promega) according to the manufacturer's instructions. The protein concentration was assessed using a BCA microdosage kit (Thermo Fisher). Briefly, exosome-like vesicles were isolated as described above and diluted in PBS to equal total protein concentration and volume and brought up to room temperature. Luminescent reagent was then added to the sample in equal volume, protected from light, and agitated at 450 rpm on an orbital shaker. Samples were incubated at room temperature for 10 min before reading on a Victor3 luminescence plate reader (PerkinElmer). Luminescence levels were normalized to protein levels and reported as activity per µL of serum.

Flow cytometric analyses of extracellular vesicles. Further details are provided in the Supplementary Methods section. Analyses were performed on a BD Canto II Special Order Research Product (BD Biosciences) equipped with a small particle option, as described previously (4, 5). The forward scatter (FSC) on this dedicated equipment is coupled to a

photomultiplier tube (PMT) with a 488 nm solid-state, 100 mW output blue laser and includes a 633 nm HeNe, 20 mW output red laser, a 405 nm solid-state diode, and a 50 mW output violet laser. The hs-FCM includes an FSC-PMT and a Fourier optical transformation unit, which reduces the background/noise and increases the angle of diffusion, thereby enhancing the detection of small-diameter particles. FCM performance tracking was performed daily before all analyses using the BD cytometer setup and tracking beads (BD Biosciences). Acquisition was performed at low speed (~10 μL/min), and to remain quantitative, a known quantity of (fluorescent) polystyrene microspheres (15 μm diameter: Polysciences) was added to each tube, and a constant number of beads detected on the basis of (auto)fluorescence was acquired for each sample throughout the study. Silica particles (Kisker Biotech GmbH & Co.) of known dimensions (100 nm, 500 nm and 1 μm in diameter) were used for instrument setup standardization. Samples were labeled in a total reaction volume of 100 μL at 37 °C for 60 min with the LWA 300 probe (125 nM) (Prof. Hermen Overkleeft, Department of Bioorganic Synthesis, Leiden University, Leiden, The Netherlands). Three microliters of Annexin V APC was added for 15 minutes at room temperature. Then, the sample was diluted by adding 100 μL of labeling buffer prior to analysis by hs-FCM. All samples were processed and analyzed by an investigator blinded to experimental conditions.

Cell lysis, protein isolation, and immunoblotting. To obtain the total extracted protein, cells were washed twice in ice-cold PBS before being lysed in lysis buffer at 4 °C for 15 minutes. The lysis buffer contained 1% Triton X-100, 150 mM NaCl, 5 mM EDTA, and 50 mM Tris (pH 7.5) and was supplemented with protease inhibitor (Calbiochem), phosphatase inhibitor cocktails and 1 mM phenylmethylsulfonyl fluoride (PMSF) (Sigma). The lysate was collected by scraping the cells with a rubber policeman and centrifuged at 12 000 x g for 10 min at 4 °C. The protein concentration was evaluated with a BCA protein assay kit (Thermo Fisher) according to the manufacturer’s instructions. The proteins were solubilized in sample buffer

(25 mM Tris·HCl, pH 6.8, 1% SDS, 0.1% bromophenol blue, 10% glycerol, and 2% β-mercaptoethanol) and incubated at 95 °C for 10 minutes before being subjected to SDS-polyacrylamide gel electrophoresis and transferred electrophoretically onto nitrocellulose membranes. The membranes were then blocked with 5% dried fat-free milk in Tris-buffered saline at pH 7.4 with 0.05% Tween 20 (TBST) for 1 h at room temperature and incubated overnight at 4 °C with specific primary antibodies in TBST plus 5% milk. The antibodies used for blotting were antibodies against PARP1 (9542; Cell Signaling Technology), TUBA1B (11224-1-AP; Proteintech), PLVAP (NB100-77668; Novus Biological), ACTB (a5441; Sigma–Aldrich), PECAM1 (AF3628-SP; R&D Systems), CD82 (ab66400; Abcam), CD81 (66866-1-IG; Thermo Fisher), SDCBP (SC-515538; Santa Cruz Biotechnology), PSMA3 (SC-67340, Santa Cruz Biotechnology and 11887-1-AP, Proteintech), HSPG2/LG3 (AF2364; R&D Systems or Polyclonal antibody against recombinant LG3; MediMabs), histone H3C1 (9715S; Cell Signaling). Following incubation, the membranes were washed with TBST and incubated with a goat anti-rabbit IgG linked to horseradish peroxidase (GE Healthcare) for 1 h, followed by Clarity Western ECL Substrate. The luminescence signal was recorded with a ChemiDoc XRS+ system (Bio-Rad Laboratories Inc.) and analyzed with Image Lab software (Bio-Rad Laboratories Inc.) to determine the intensity of each specific band.

Animal studies. Caspase-3-deficient mice (Casp-3 -/-) were bred from heterozygous Casp-3 -/- (B6.129S1-C3tm1Flv/J) mice obtained from Jackson Laboratory (stock #006233; Bar Harbor, ME) and were 6-8 weeks old. These mice were viable and reached adulthood, but exhibited various abnormal cell arrangements and hyperplasias in the brain. The mice were housed in a 12-hour light/dark cycle and had access to food ad libitum. Ischemia-reperfusion injury (IRI) by unilateral renal artery clamping plus contralateral nephrectomy was performed as described.

Renal ischemia-reperfusion surgery procedures. Preparation before surgery: Sterile surgical instruments are prepared before the surgery. The body weight of each mouse is taken and

recorded on the surgical sheet. Then, the mouse is anesthetized with 2%-3% isoflurane and 1 L/min oxygen. Ophthalmic lubricant is administered to protect both eyes. Carprofen at a dosage of 10 mg/kg is administered subcutaneously to control pain. The fur on the abdomen region is removed using an electric shaver and cleaned with moisturized gauze. Depilatory cream is applied to the same region to clean the abdominal skin, and the cream is removed with cotton swabs. Surgery: Immediately after skin preparation, the mouse is placed on a homeothermic blanket. Surgery will not begin until the mouse is in deep anesthesia and does not respond to pain induced by a toe pinch. The surgical area is disinfected, and sterile surgical gloves are used to perform the surgery. A sterile surgical rodent drape is placed over the surgical area. Bupivacaine 0.25% (Marcaine, 2 mg/kg) is administered subcutaneously in the surgical wound area. A mid-abdominal incision is made with surgical scissors, and the abdominal cavity is exposed. The intestine is gently moved aside to expose the left kidney. The left renal pedicle is exposed using sterile cotton swabs. Dissection of the pedicle tissue is performed with ultra-fine-point tweezers to remove the tissue around the renal pedicle, exposing the blood vessels for renal pedicle clamping. A micro-aneurysm clamp is used to block blood flow to the kidney, inducing renal ischemia. Complete ischemia is indicated by a color change of the kidney from red to dark purple. After the pedicle clamping, the intestine is returned to its original position in the abdominal cavity. The surgical wound is covered with moist sterile gauze during the 30 or 60 minutes of ischemia. The mouse remains under anesthesia throughout renal artery clamping and the color of the left kidney is monitored during this period. After 30 or 60 minutes of ischemia, the micro-aneurysm clip is released from the left kidney to initiate reperfusion, indicated by the change in kidney color back to red. The nephrectomy of the right kidney is then performed. Immediately after wound closure, 0.5 mL of sterile saline and 0.3 mL of 2.5% dextrose are administered subcutaneously to each mouse. The body weight of the mouse is recorded on the surgical sheet. The animal is then kept in an incubator for recovery. The mouse

is returned to its housing cage with an isopad at the bottom after it regains full consciousness. Chocolate Ensure® mixed soft food is provided. Monitoring the success of renal ischemia-reperfusion: After clamping, the kidney color should change from red to dark purple, indicative of successful renal ischemia. The immediate color change at the very beginning requires careful observation. However, the kidney will transition to a deep dark purple color several minutes later. After removing the clips, the kidney color should change back to red to indicate reperfusion. Post-op care: Each mouse is examined twice daily for the first two days after surgery to assess its general condition, body weight, surgical wound, and food intake. Carprofen at a dosage of 10 mg/kg is administered subcutaneously once daily. Additionally, 0.3 mL of 2.5% dextrose and 0.5 mL of saline are given subcutaneously twice daily, depending on the hydration status of the mouse. Chocolate Ensure® mixed soft food is provided for the mouse each day. All data are recorded on the surgical sheet. The endpoint is a loss of 20% body weight.

Subcapsular injection procedures. After 30 minutes of ischemia, 50 µL of miRIDIAN microRNA mmu-miR-423-5p mimic or miRIDIAN microRNA Mimic negative control #1, mixed with *in vivo*-jetPEI reagent, was injected into the renal capsule using a 33-gauge, small hub RN 2-inch needle with a Hamilton syringe. The kidney was kept moist with warm sterile saline prior to the delivery of the miRNA. The needle of the syringe was inserted into the kidney capsule at the upper middle portion. The needle was then slowly moved from the upper end to the lower end along the inner surface of the renal capsule. The needle was pressed to slowly inject the miRNA, after which the syringe was carefully withdrawn, and the injection site was pressed with a cotton swab for 1 minute. Two days after injection, the kidney was harvested and cross-sectioned into four segments. The expression levels of miR-423-5p were measured by quantitative PCR in each quadrant to validate the diffusion of the miRNA throughout the kidney.

Cohort study of renal transplant patients.

Measurements. To identify other variables associated with PTC density and fibrosis, we collected data on recipient age, sex, height, weight, renal disease, number of previous transplantations, pretransplantation panel reactive antibodies, medication before and after transplantation, comorbidities, smoking habits, total ischemic time, induction and maintenance immunosuppressive protocols, allograft acute rejection occurring before or based on the post-transplantation biopsy defined per the Banff 2019 classification (6), and donor type, sex, age, height, weight, hypertension, diabetes, and cardiovascular disease. Donor comorbidities such as hypertension and diabetes were defined as the clinical diagnoses provided by the organ donation organization transplant coordinators to the transplant team. After cardiocirculatory arrest (DCD), all donors were maintained under controlled conditions (Maastricht category 3), awaiting death in the operating room. We had no access to information on the length of the agonal phase.

Renal tubular damage. Mice were euthanized at baseline, and at 2 or 21 days following renal ischemia-reperfusion injury (IRI) via cardiac puncture. The kidneys were collected, fixed in neutral buffered formalin (ChapTec), and embedded in paraffin. Samples were sectioned into 4 µm slices and automatically stained with hematoxylin-eosin (H&E). Renal tubular damage was assessed on the H&E stained sections using the following criteria: tubular dilatation, loss of brush border, formation of luminal casts, tubular necrosis, and infiltration by polynuclear neutrophils. The extent of damaged tubules was quantified as a tubular injury score (TIS), with the following scale: 0 (no damaged tubules; normal kidney), 1 (< 10% damaged tubules; mild injury), 2 (11-25% damaged tubules; moderate injury), 3 (26-49% damaged tubules; severe injury), 4 (50-75% damaged tubules; high severe injury), and 5 (> 75% damaged tubules; extensive injury).

Renal tubular epithelial cell culture. PT-2 tubular epithelial cells (TECs) (kind gift of A. Jevnikar, Western University, London, Ontario, Canada) is a human renal tubular cell line isolated and cloned from centrifuged urine, which was obtained from a transplant recipient undergoing acute rejection (7). PT-2 cells were cultured in K1 medium constituted with following products: Dulbecco's modified Eagle's medium (DMEM): Hams F12 (50:50) (Invitrogen-Gibco), 5% fetal bovine serum (Invitrogen), hormone mix (5 µg/mL insulin, 1.25 ng/mL prostaglandin E1, 34 pg/mL triiodothyronine, 5 µg/mL transferrin, 1.73 ng/mL sodium selenite and 18 ng/mL of hydrocortisone) and 25 ng/mL epidermal growth factor. PT-2 cells were grown in normal medium until confluence, then exposed to serum-free medium for 24 hours to generate apoptotic exosome-like vesicles.

(1) Migneault F, et al. Apoptotic exosome-like vesicles regulate endothelial gene expression, inflammatory signaling, and function through the NF-κB signaling pathway. *Sci Rep.* 2020;10(1):12562.

(2) Geback T, et al. TScratch: a novel and simple software tool for automated analysis of monolayer wound healing assays. Biotechniques. 2009;46(4):265-274.

(3) Carpentier G. Angiogenesis Analyzer for ImageJ - Gilles Carpentier Research Web Site: Computer Image Analysis. Available at: http://image.bio.methods.free.fr/ImageJ/?Angiogenesis-Analyzerfor-ImageJ&artpage=2-6. Accessed November 02, 2016.

(4) Marcoux G, et al. Revealing the diversity of extracellular vesicles using high-dimensional flow cytometry analyses. Sci Rep. 2016;6:35928.

(5) Rousseau M, et al. Detection and quantification of microparticles from different cellular lineages using flow cytometry. Evaluation of the impact of secreted phospholipase A2 on microparticle assessment. PLoS One. 2015;10(1):e0116812.

(6) Loupy A, et al. The Banff 2019 Kidney Meeting Report (I): Updates on and clarification of criteria for T cell- and antibody-mediated rejection. Am J Transplant. 2020;20(9):2318-2331.

(7) Wang S, et al. Anti-IL-2 receptor antibody decreases cytokine-induced apoptosis of human renal tubular epithelial cells (TEC). Nephrol Dial Transplant. 2011 Jul;26(7):2144-53.

Supplementary Table 1. List of microRNAs expressed in ApoExo, ApoBodies and endothelial cells in normal (HUVEC_N) or serum-starved (HUVEC_SS) conditions.

#miRNA	ApoExo1	ApoExo2	ApoBodies1	ApoBodies2	NHUV1	NHUV2	SSHUV1	SSHUV2	DEG_ApoExo_ApoB1	DEG_ApoExo_ApoB2	DEG_ApoExo_N-H1	DEG_ApoExo_N-H2	DEG_ApoExo_SS-H1	DEG_ApoExo_SS-H2	FPKM min ApoExo	DEG_ApoExo >2	DEG_MEAN
hsa-miR-423-5p	7107	9619	628	1679	566	603	633	1111	11,3	5,7	12,5	16,0	11,2	8,7	VRAI	VRAI	10,9
hsa-let-7b-5p	139427	131682	14159	27627	10065	11346	22081	20583	9,8	4,8	13,9	11,6	6,3	6,4	VRAI	VRAI	8,8
hsa-miR-30a-3p	8198	6298	1424	1461	1328	1133	1592	1512	5,8	4,3	6,2	5,6	5,1	4,2	VRAI	VRAI	5,2
hsa-let-7c-5p	155845	142703	43046	69373	30749	37138	57902	51044	3,6	2,1	5,1	3,8	2,7	2,8	VRAI	VRAI	3,3
hsa-miR-16-5p	13796	16904	5927	3570	4150	3566	4504	4774	2,3	4,7	3,3	4,7	3,1	3,5	VRAI	VRAI	3,6
hsa-miR-941	6965	6602	1112	1032	1190	2469	2361	2295	6,3	6,4	5,9	2,7	2,9	2,9	VRAI	VRAI	4,5
hsa-miR-30e-3p	5139	3751	1070	1091	1029	851	1160	1126	4,8	3,4	5,0	4,4	4,4	3,3	FAUX	VRAI	4,2
hsa-miR-4448	264	446	23	113	56	22	46	93	11,6	4,0	4,7	20,2	5,8	4,8	FAUX	VRAI	8,5
hsa-miR-122-5p	60	160	5	23	3	4	0	1	12,2	6,8	22,7	38,6	5966,0	125,0	FAUX	VRAI	1028,6
hsa-miR-10b-3p	80	78	10	19	21	10	11	13	8,2	4,1	3,8	8,1	7,2	6,1	FAUX	VRAI	6,2
hsa-miR-760	44	53	5	16	1	6	11	8	9,1	3,2	33,7	8,6	4,0	7,0	FAUX	VRAI	10,9
hsa-miR-766-5p	25	26	6	2	3	4	4	5	3,8	10,8	9,3	6,2	6,2	5,0	FAUX	VRAI	6,9
hsa-miR-193b-5p	13	30	2	9	1	5	2	4	7,9	3,2	9,8	6,2	5,4	7,9	FAUX	VRAI	6,7
hsa-miR-4436b-3p	12	11	0	0	0	1	2	3	1171,0	1113,0	1171,0	8,0	7,4	4,4	FAUX	VRAI	579,1
hsa-miR-432-5p	1923	3195	627	1109	802	823	738	659	3,1	2,9	2,4	3,9	2,6	4,8	FAUX	VRAI	3,3
hsa-miR-1180-3p	953	959	101	174	118	341	325	313	9,5	5,5	8,1	2,8	2,9	3,1	FAUX	VRAI	5,3
hsa-miR-320d	597	989	237	253	37	54	55	79	2,5	3,9	16,3	18,4	10,8	12,6	FAUX	VRAI	10,7
hsa-miR-320e	301	492	117	124	22	26	32	41	2,6	4,0	13,5	18,8	9,5	12,1	FAUX	VRAI	10,1
hsa-miR-4429	191	263	65	73	10	17	18	20	2,9	3,6	18,2	15,3	10,5	13,0	FAUX	VRAI	10,6
hsa-miR-877-5p	41	111	10	45	10	6	13	23	4,2	2,5	3,9	17,9	3,2	4,9	FAUX	VRAI	6,1
hsa-miR-3187-3p	53	53	5	9	4	7	13	22	10,8	5,7	13,4	7,7	3,9	2,5	FAUX	VRAI	7,3
hsa-miR-337-5p	22	71	3	7	8	4	2	1	6,8	10,1	2,8	17,1	9,3	55,6	FAUX	VRAI	17,0
hsa-miR-3138	15	16	2	5	3	1	6	0	9,3	3,3	5,8	22,2	2,8	1557,0	FAUX	VRAI	266,7
hsa-miR-4510	8	8	0	2	0	1	2	4	820,0	3,3	820,0	11,1	3,4	2,0	FAUX	VRAI	276,7
hsa-miR-320b	11905	26455	4451	8514	3593	5196	6649	9905	2,7	3,1	3,3	5,1	1,8	2,7	VRAI	FAUX	3,1
hsa-miR-320a	8389	17251	3377	6361	3012	4437	5286	7619	2,5	2,7	2,8	3,9	1,6	2,3	VRAI	FAUX	2,6
hsa-let-7a-5p	254788	203249	192158	275698	133487	150235	212043	183710	1,3	0,7	1,9	1,4	1,2	1,1	VRAI	FAUX	1,3
hsa-let-7f-5p	54207	39979	106462	143650	72821	88785	114299	97499	0,5	0,3	0,7	0,5	0,5	0,4	VRAI	FAUX	0,5
hsa-miR-181b-5p	33763	38931	21599	29121	20333	27131	28802	26648	1,6	1,3	1,7	1,4	1,2	1,5	VRAI	FAUX	1,4
hsa-miR-10b-5p	34836	24935	13441	10435	24421	10739	12438	15306	2,6	2,4	1,4	2,3	2,8	1,6	VRAI	FAUX	2,2
hsa-miR-10a-5p	34427	24547	13195	10325	24081	10616	12219	15039	2,6	2,4	1,4	2,3	2,8	1,6	VRAI	FAUX	2,2
hsa-miR-181a-5p	26974	26909	47767	40843	36010	91520	54983	25308	0,6	0,7	0,7	0,3	0,5	1,1	VRAI	FAUX	0,6
hsa-miR-92a-3p	16835	23160	23814	15158	33225	76041	53316	59458	0,7	1,5	0,5	0,3	0,3	0,4	VRAI	FAUX	0,6
hsa-let-7e-5p	20944	16848	33467	51377	25320	28588	40901	35683	0,6	0,3	0,8	0,6	0,5	0,5	VRAI	FAUX	0,6
hsa-miR-181d-5p	15989	17798	9113	12224	8429	11248	12368	11172	1,8	1,5	1,9	1,6	1,3	1,6	VRAI	FAUX	1,6
hsa-let-7i-5p	9279	17540	55015	32010	25783	34022	37309	29420	0,2	0,5	0,4	0,5	0,2	0,6	VRAI	FAUX	0,4
hsa-miR-21-5p	9093	15077	95271	39872	170079	50654	56945	87682	0,1	0,4	0,1	0,3	0,2	0,2	VRAI	FAUX	0,2
hsa-miR-221-3p	7193	16290	44165	35407	43360	59353	33071	38521	0,2	0,5	0,2	0,3	0,2	0,4	VRAI	FAUX	0,3
hsa-miR-24-3p	7072	14610	9386	7407	12486	9226	8264	10115	0,8	2,0	0,6	1,6	0,9	1,4	VRAI	FAUX	1,2
hsa-miR-26a-5p	8809	10534	16571	15684	19435	13826	15315	15727	0,5	0,7	0,5	0,8	0,6	0,7	VRAI	FAUX	0,6
hsa-miR-100-5p	7313	7694	2245	2552	4971	3301	2419	2601	3,3	3,0	1,5	2,3	3,0	3,0	VRAI	FAUX	2,7
hsa-miR-99a-5p	6983	7323	2172	2416	4746	3161	2330	2492	3,2	3,0	1,5	2,3	3,0	2,9	VRAI	FAUX	2,7
hsa-miR-423-3p	4600	5377	1753	1935	1588	3084	2694	3123	2,6	2,8	2,9	1,7	1,7	1,7	FAUX	FAUX	2,2
hsa-miR-199a-3p	6393	5415	6021	3314	3806	6227	4850	4537	1,1	1,6	1,7	0,9	1,3	1,2	FAUX	FAUX	1,3
hsa-miR-199b-3p	6393	5415	6021	3314	3806	6227	4850	4537	1,1	1,6	1,7	0,9	1,3	1,2	FAUX	FAUX	1,3
hsa-miR-155-5p	4213	6034	8948	6856	3817	7336	7806	6747	0,5	0,9	1,1	0,8	0,5	0,9	FAUX	FAUX	0,8
hsa-miR-222-3p	3862	5758	19375	15248	18464	27620	17694	22419	0,2	0,4	0,2	0,2	0,2	0,3	FAUX	FAUX	0,2
hsa-miR-26b-5p	4486	3703	3190	3516	4981	3565	3677	3934	1,4	1,1	0,9	1,0	1,2	0,9	FAUX	FAUX	1,1
hsa-miR-320c	1367	2151	873	910	290	423	480	623	1,6	2,4	4,7	5,1	2,8	3,5	FAUX	FAUX	3,3
hsa-miR-421	367	542	185	159	139	154	143	172	2,0	3,4	2,6	3,5	2,6	3,1	FAUX	FAUX	2,9
hsa-miR-1290	418	277	196	169	4	3	10	15	2,1	1,6	106,3	99,9	40,7	18,2	FAUX	FAUX	44,8
hsa-miR-502-3p	49	47	16	28	26	28	21	30	3,0	1,7	1,9	1,7	2,3	1,5	FAUX	FAUX	2,0
hsa-miR-1262	37	50	23	33	10	23	21	15	1,6	1,5	3,6	2,1	1,8	3,3	FAUX	FAUX	2,3
hsa-miR-5096	15	24	8	14	3	9	6	4	1,9	1,7	5,8	2,7	2,4	6,4	FAUX	FAUX	3,5
hsa-miR-4804-5p	13	10	3	5	5	3	6	5	4,0	2,1	2,5	3,6	2,3	2,0	FAUX	FAUX	2,7
hsa-miR-744-3p	7	7	3	2	4	3	2	0	2,2	2,8	1,8	2,4	3,0	668,0	FAUX	FAUX	113,4
hsa-miR-22-3p	2802	7324	10355	5167	16616	11796	5634	8663	0,3	1,4	0,2	0,6	0,5	0,8	FAUX	FAUX	0,6
hsa-miR-126-5p	3211	3869	16910	6469	27984	8432	11844	20535	0,2	0,6	0,1	0,5	0,3	0,2	FAUX	FAUX	0,3
hsa-miR-103a-3p	2384	4421	15755	9537	20087	19064	13319	16794	0,2	0,5	0,1	0,2	0,2	0,3	FAUX	FAUX	0,2
hsa-let-7g-5p	2632	3569	10449	9565	5911	8534	9730	7596	0,3	0,4	0,4	0,4	0,3	0,5	FAUX	FAUX	0,4
hsa-let-7d-5p	2567	3306	3932	6267	2553	3550	5345	5359	0,7	0,5	1,0	0,9	0,5	0,6	FAUX	FAUX	0,7
hsa-miR-25-3p	2169	2681	3153	1555	2862	3104	2882	3803	0,7	1,7	0,8	0,9	0,8	0,7	FAUX	FAUX	0,9
hsa-miR-98-5p	2769	2065	4472	3957	2320	2386	3246	2549	0,6	0,5	1,2	0,9	0,9	0,8	FAUX	FAUX	0,8
hsa-miR-34a-5p	1504	3251	3092	2606	2348	2724	1934	1482	0,5	1,2	0,6	1,2	0,8	2,2	FAUX	FAUX	1,1
hsa-miR-99b-5p	1878	2842	1060	1044	1506	1543	1123	1247	1,8	2,7	1,2	1,8	1,7	2,3	FAUX	FAUX	1,9
hsa-miR-151b	1407	2192	1594	1501	1426	1562	1317	1386	0,9	1,5	1,0	1,4	1,1	1,6	FAUX	FAUX	1,2
hsa-miR-191-5p	1123	2381	2700	1956	3253	4344	3132	3288	0,4	1,2	0,3	0,5	0,4	0,7	FAUX	FAUX	0,6
hsa-miR-151a-3p	1400	1994	4261	2542	4536	4021	3537	3936	0,3	0,8	0,3	0,5	0,4	0,5	FAUX	FAUX	0,5
hsa-miR-93-5p	953	2094	4128	2374	3230	2652	4206	4651	0,2	0,9	0,3	0,8	0,2	0,5	FAUX	FAUX	0,5
hsa-miR-107	1046	1999	6682	4196	8339	7933	5706	7361	0,2	0,5	0,1	0,3	0,2	0,3	FAUX	FAUX	0,2
hsa-miR-28-3p	772	1766	1118	880	1622	1936	1440	1447	0,7	2,0	0,5	0,9	0,5	1,2	FAUX	FAUX	1,0
hsa-miR-106b-3p	1435	1064	567	643	492	719	553	708	2,5	1,7	2,9	1,5	2,6	1,5	FAUX	FAUX	2,1
hsa-miR-126-3p	787	1698	8078	3800	7814	3745	3984	5909	0,1	0,4	0,1	0,5	0,2	0,3	FAUX	FAUX	0,3
hsa-miR-378a-3p	1232	1205	1250	683	562	2051	1125	968	1,0	1,8	2,2	0,6	1,1	1,2	FAUX	FAUX	1,3
hsa-miR-101-3p	1074	1171	584	450	1745	418	235	489	1,8	2,6	0,6	2,8	4,6	2,4	FAUX	FAUX	2,5
hsa-miR-196a-5p	846	1387	1990	2474	1249	1387	1687	1646	0,4	0,6	0,7	1,0	0,5	0,8	FAUX	FAUX	0,7
hsa-miR-217	1121	1055	2659	1004	5316	976	1684	2730	0,4	1,1	0,2	1,1	0,7	0,4	FAUX	FAUX	0,6
hsa-miR-130b-3p	697	1419	992	561	820	714	773	785	0,7	2,5	0,8	2,0	0,9	1,8	FAUX	FAUX	1,5
hsa-miR-17-5p	520	1557	1528	842	4090	1001	1673	1889	0,3	1,8	0,1	1,6	0,3	0,8	FAUX	FAUX	0,8
hsa-miR-106a-5p	516	1518	1370	755	3873	911	1539	1760	0,4	2,0	0,1	1,7	0,3	0,9	FAUX	FAUX	0,9
hsa-miR-151a-5p	725	1126	1000	924	935	1005	880	956	0,7	1,2	0,8	1,1	0,8	1,2	FAUX	FAUX	1,0
hsa-miR-30a-5p	779	1015	11553	3804	14447	13030	8824	10337	0,1	0,3	0,1	0,1	0,1	0,1	FAUX	FAUX	0,1
hsa-miR-1246	978	707	618	636	35	17	71	107	1,6	1,1	27,7	41,0	13,8	6,6	FAUX	FAUX	15,3
hsa-miR-30d-5p	723	952	10732	3586	13316	12189	8282	9513	0,1	0,3	0,1	0,1	0,1	0,1	FAUX	FAUX	0,1
hsa-miR-1307-3p	837	757	295	865	528	795	788	1056	2,8	0,9	1,6	1,0	1,1	0,7	FAUX	FAUX	1,3
hsa-miR-30e-5p	655	870	9479	3019	11593	10514	7156	8122	0,1	0,3	0,1	0,1	0,1	0,1	FAUX	FAUX	0,1
hsa-miR-532-5p	684	822	456	382	544	646	316	401	1,5	2,2	1,3	1,3	2,2	2,1	FAUX	FAUX	1,7
hsa-miR-19b-3p	490	982	664	751	1924	417	360	1745	0,7	1,3	0,3	2,4	1,4	0,6	FAUX	FAUX	1,1
hsa-miR-146a-5p	459	835	1557	713	1173	9196	1090	1084	0,3	1,2	0,4	0,1	0,4	0,8	FAUX	FAUX	0,5
hsa-miR-574-5p	421	789	172	678	394	190	193	427	2,4	1,2	1,1	4,1	2,2	1,8	FAUX	FAUX	2,1
hsa-miR-27b-3p	319	868	1617	1159	3156	1097	725	1245	0,2	0,7	0,1	0,8	0,4	0,7	FAUX	FAUX	0,5
hsa-miR-196b-5p	454	711	1234	1391	802	803	990	966	0,4	0,5	0,6	0,9	0,5	0,7	FAUX	FAUX	0,6
hsa-miR-409-3p	280	880	1584	1525	2666	3951	1683	1784	0,2	0,6	0,1	0,2	0,2	0,5	FAUX	FAUX	0,3
hsa-miR-27a-3p	284	772	1339	987	2751	950	600	975	0,2	0,8	0,1	0,8	0,5	0,8	FAUX	FAUX	0,5
hsa-miR-21-3p	587	460	304	249	1254	1027	572	621	1,9	1,9	0,5	0,4	1,0	0,7	FAUX	FAUX	1,1
hsa-miR-148b-3p	352	654	1130	647	1653	1005	597	770	0,3	1,0	0,2	0,7	0,6	0,8	FAUX	FAUX	0,6
hsa-miR-152-3p	344	619	330	324	281	299	317	280	1,0	1,9	1,2	2,1	1,1	2,2	FAUX	FAUX	1,6
hsa-miR-186-5p	222	706	1032	699	1999	1874	702	1323	0,2	1,0	0,1	0,4	0,3	0,5	FAUX	FAUX	0,4
hsa-miR-769-5p	504	418	187	218	366	269	151	232	2,7	1,9	1,4	1,6	3,3	1,8	FAUX	FAUX	2,1
hsa-miR-20a-5p	284	583	849	361	900	579	854	950	0,3	1,6	0,3	1,0	0,3	0,6	FAUX	FAUX	0,7
hsa-miR-660-5p	328	468	153	141	263	79	56	164	2,1	3,3	1,2	6,0	5,9	2,9	FAUX	FAUX	3,6
hsa-miR-29a-3p	262	528	627	612	1160	632	249	576	0,4	0,9	0,2	0,8	1,1	0,9	FAUX	FAUX	0,7
hsa-miR-23a-3p	146	642	308	399	574	285	103	153	0,5	1,6	0,3	2,3	1,4	4,2	FAUX	FAUX	1,7
hsa-miR-19a-3p	246	493	307	347	925	201	171	827	0,8	1,4	0,3	2,5	1,4	0,6	FAUX	FAUX	1,2
hsa-miR-23b-3p	143	595	346	413	583	292	110	179	0,4	1,4	0,2	2,0	1,3	3,3	FAUX	FAUX	1,5
hsa-miR-29c-3p	223	495	597	596	1117	603	232	557	0,4	0,8	0,2	0,8	1,0	0,9	FAUX	FAUX	0,7
hsa-miR-382-5p	151	500	878	851	957	1173	760	818	0,2	0,6	0,2	0,4	0,2	0,6	FAUX	FAUX	0,4
hsa-miR-140-3p	209	428	580	202	848	850	499	716	0,4	2,1	0,2	0,5	0,4	0,6	FAUX	FAUX	0,7
hsa-miR-181c-5p	282	347	300	284	379	68	57	208	0,9	1,2	0,7	5,1	5,0	1,7	FAUX	FAUX	2,4
hsa-miR-31-5p	229	375	9323	3087	13066	19543	8331	11444	0,0	0,1	0,0	0,0	0,0	0,0	FAUX	FAUX	0,0
hsa-miR-7-5p	261	297	4599	1893	2504	2098	4417	3650	0,1	0,2	0,1	0,1	0,1	0,1	FAUX	FAUX	0,1
hsa-miR-28-5p	201	336	279	321	528	182	152	330	0,7	1,0	0,4	1,8	1,3	1,0	FAUX	FAUX	1,1
hsa-miR-493-5p	261	228	297	281	310	1038	545	619	0,9	0,8	0,8	0,2	0,5	0,4	FAUX	FAUX	0,6
hsa-miR-574-3p	147	311	143	417	416	669	349	370	1,0	0,7	0,4	0,5	0,4	0,8	FAUX	FAUX	0,6
hsa-miR-382-3p	235	203	31	84	327	123	58	88	7,6	2,4	0,7	1,6	4,1	2,3	FAUX	FAUX	3,1
hsa-miR-134-5p	104	315	458	288	542	822	470	489	0,2	1,1	0,2	0,4	0,2	0,6	FAUX	FAUX	0,5
hsa-miR-99b-3p	138	263	179	204	212	272	272	289	0,8	1,3	0,7	1,0	0,5	0,9	FAUX	FAUX	0,9
hsa-miR-192-5p	164	197	76	80	141	61	82	120	2,1	2,5	1,2	3,2	2,0	1,6	FAUX	FAUX	2,1
hsa-miR-503-5p	153	191	196	99	196	54	147	199	0,8	1,9	0,8	3,5	1,0	1,0	FAUX	FAUX	1,5
hsa-miR-744-5p	127	216	117	87	90	229	236	318	1,1	2,5	1,4	0,9	0,5	0,7	FAUX	FAUX	1,2
hsa-miR-125a-3p	118	220	73	120	71	57	85	93	1,6	1,8	1,7	3,8	1,4	2,4	FAUX	FAUX	2,1
hsa-miR-130a-3p	85	232	362	138	259	144	182	217	0,2	1,7	0,3	1,6	0,5	1,1	FAUX	FAUX	0,9
hsa-miR-181a-2-3p	173	141	255	174	411	427	370	361	0,7	0,8	0,4	0,3	0,5	0,4	FAUX	FAUX	0,5
hsa-miR-10a-3p	137	177	81	56	112	99	75	82	1,7	3,1	1,2	1,8	1,8	2,1	FAUX	FAUX	2,0
hsa-miR-15a-5p	132	178	97	66	76	63	56	60	1,4	2,7	1,7	2,8	2,4	3,0	FAUX	FAUX	2,3
hsa-miR-128-3p	105	205	854	483	1135	960	1090	1414	0,1	0,4	0,1	0,2	0,1	0,1	FAUX	FAUX	0,2
hsa-miR-125b-2-3p	116	183	239	164	235	212	360	341	0,5	1,1	0,5	0,9	0,3	0,5	FAUX	FAUX	0,6
hsa-miR-369-3p	85	205	196	220	1139	413	120	297	0,4	0,9	0,1	0,5	0,7	0,7	FAUX	FAUX	0,6
hsa-miR-132-3p	124	158	57	80	93	121	53	88	2,2	2,0	1,3	1,3	2,3	1,8	FAUX	FAUX	1,8
hsa-miR-324-5p	78	200	109	138	98	86	59	105	0,7	1,4	0,8	2,3	1,3	1,9	FAUX	FAUX	1,4
hsa-miR-323a-3p	87	182	130	145	402	722	203	309	0,7	1,3	0,2	0,3	0,4	0,6	FAUX	FAUX	0,6
hsa-miR-370-3p	76	186	443	593	370	692	455	576	0,2	0,3	0,2	0,3	0,2	0,3	FAUX	FAUX	0,2
hsa-miR-455-5p	88	157	130	106	191	118	87	153	0,7	1,5	0,5	1,3	1,0	1,0	FAUX	FAUX	1,0
hsa-miR-433-3p	48	192	73	176	144	156	118	142	0,7	1,1	0,3	1,2	0,4	1,4	FAUX	FAUX	0,8
hsa-miR-889-3p	87	150	54	101	272	113	51	109	1,6	1,5	0,3	1,3	1,7	1,4	FAUX	FAUX	1,3
hsa-miR-378i	87	147	144	82	102	213	125	115	0,6	1,8	0,8	0,7	0,7	1,3	FAUX	FAUX	1,0
hsa-miR-378c	82	140	141	80	99	203	124	114	0,6	1,8	0,8	0,7	0,7	1,2	FAUX	FAUX	1,0
hsa-miR-493-3p	70	151	58	59	43	83	55	49	1,2	2,6	1,6	1,8	1,3	3,1	FAUX	FAUX	1,9
hsa-miR-181a-3p	106	113	273	246	761	266	415	487	0,4	0,5	0,1	0,4	0,3	0,2	FAUX	FAUX	0,3
hsa-miR-224-5p	89	127	84	91	81	103	61	85	1,1	1,4	1,1	1,2	1,5	1,5	FAUX	FAUX	1,3
hsa-miR-139-5p	105	109	78	54	58	10	14	18	1,4	2,0	1,8	11,3	7,4	6,1	FAUX	FAUX	5,0
hsa-miR-127-3p	68	142	148	124	238	281	122	84	0,5	1,1	0,3	0,5	0,6	1,7	FAUX	FAUX	0,8
hsa-miR-361-3p	46	143	151	122	179	273	123	178	0,3	1,2	0,3	0,5	0,4	0,8	FAUX	FAUX	0,6
hsa-miR-216a-3p	90	96	284	206	505	182	438	451	0,3	0,5	0,2	0,5	0,2	0,2	FAUX	FAUX	0,3
hsa-miR-216a-5p	94	87	50	26	147	46	36	47	1,9	3,4	0,6	1,9	2,6	1,8	FAUX	FAUX	2,0
hsa-miR-125b-1-3p	78	97	187	115	154	148	163	127	0,4	0,8	0,5	0,7	0,5	0,8	FAUX	FAUX	0,6
hsa-miR-185-5p	28	141	57	47	101	54	43	76	0,5	3,0	0,3	2,6	0,7	1,9	FAUX	FAUX	1,5
hsa-miR-92b-3p	73	97	179	138	386	1532	935	1106	0,4	0,7	0,2	0,1	0,1	0,1	FAUX	FAUX	0,3
hsa-miR-497-5p	53	116	97	66	149	147	60	90	0,5	1,8	0,4	0,8	0,9	1,3	FAUX	FAUX	0,9
hsa-miR-424-3p	55	107	123	56	187	136	392	457	0,4	1,9	0,3	0,8	0,1	0,2	FAUX	FAUX	0,6
hsa-miR-30c-2-3p	78	82	96	87	102	99	124	112	0,8	0,9	0,8	0,8	0,6	0,7	FAUX	FAUX	0,8
hsa-miR-345-5p	73	88	393	331	845	582	375	718	0,2	0,3	0,1	0,2	0,2	0,1	FAUX	FAUX	0,2
hsa-miR-543	34	122	63	103	198	397	178	222	0,5	1,2	0,2	0,3	0,2	0,6	FAUX	FAUX	0,5
hsa-miR-381-3p	30	117	91	61	612	269	55	203	0,3	1,9	0,0	0,4	0,6	0,6	FAUX	FAUX	0,6
hsa-miR-548av-3p	113	28	16	23	42	110	16	22	7,0	1,2	2,7	0,3	7,2	1,3	FAUX	FAUX	3,3
hsa-miR-654-3p	44	94	166	218	908	922	217	510	0,3	0,4	0,0	0,1	0,2	0,2	FAUX	FAUX	0,2
hsa-miR-324-3p	32	101	42	61	38	54	31	81	0,7	1,7	0,8	1,9	1,0	1,2	FAUX	FAUX	1,2
hsa-miR-218-5p	61	71	334	169	755	241	244	369	0,2	0,4	0,1	0,3	0,2	0,2	FAUX	FAUX	0,2
hsa-miR-181c-3p	62	66	68	54	78	31	37	43	0,9	1,2	0,8	2,1	1,7	1,5	FAUX	FAUX	1,4
hsa-miR-629-5p	51	73	213	115	95	194	160	129	0,2	0,6	0,5	0,4	0,3	0,6	FAUX	FAUX	0,4
hsa-miR-486-5p	66	59	70	19	31	102	71	48	0,9	3,1	2,1	0,6	0,9	1,2	FAUX	FAUX	1,5
hsa-miR-548ad-5p	76	48	37	45	33	40	42	46	2,0	1,1	2,3	1,2	1,8	1,0	FAUX	FAUX	1,6
hsa-miR-548ae-5p	76	48	37	45	33	40	42	46	2,0	1,1	2,3	1,2	1,8	1,0	FAUX	FAUX	1,6
hsa-miR-487b-3p	27	96	88	110	275	318	126	143	0,3	0,9	0,1	0,3	0,2	0,7	FAUX	FAUX	0,4
hsa-miR-584-5p	51	68	169	56	78	61	83	86	0,3	1,2	0,7	1,1	0,6	0,8	FAUX	FAUX	0,8
hsa-miR-379-5p	35	83	778	380	933	733	529	558	0,0	0,2	0,0	0,1	0,1	0,1	FAUX	FAUX	0,1
hsa-miR-15b-5p	43	71	42	40	33	31	17	39	1,0	1,8	1,3	2,3	2,5	1,8	FAUX	FAUX	1,8
hsa-miR-361-5p	37	77	101	56	169	165	109	124	0,4	1,4	0,2	0,5	0,3	0,6	FAUX	FAUX	0,6
hsa-miR-301a-3p	36	77	97	61	80	14	48	43	0,4	1,3	0,5	5,6	0,8	1,8	FAUX	FAUX	1,7
hsa-miR-30c-5p	35	78	724	366	1795	1312	946	1793	0,0	0,2	0,0	0,1	0,0	0,0	FAUX	FAUX	0,1
hsa-miR-1307-5p	44	61	39	101	175	166	32	55	1,1	0,6	0,3	0,4	1,4	1,1	FAUX	FAUX	0,8
hsa-miR-500a-3p	50	53	18	38	29	35	27	38	2,8	1,4	1,7	1,5	1,9	1,4	FAUX	FAUX	1,8
hsa-miR-887-3p	36	64	115	171	313	210	184	358	0,3	0,4	0,1	0,3	0,2	0,2	FAUX	FAUX	0,2
hsa-miR-340-5p	29	69	114	66	330	99	85	153	0,3	1,0	0,1	0,7	0,3	0,4	FAUX	FAUX	0,5
hsa-miR-501-3p	49	49	15	33	27	30	22	33	3,4	1,5	1,8	1,6	2,2	1,5	FAUX	FAUX	2,0
hsa-miR-376a-3p	19	78	45	38	238	62	35	48	0,4	2,1	0,1	1,3	0,5	1,6	FAUX	FAUX	1,0
hsa-miR-425-3p	34	60	49	63	76	81	44	60	0,7	0,9	0,4	0,7	0,8	1,0	FAUX	FAUX	0,8
hsa-miR-548ay-5p	54	40	23	35	25	27	30	34	2,4	1,1	2,2	1,5	1,8	1,2	FAUX	FAUX	1,7
hsa-miR-411-5p	35	58	594	274	1209	430	328	402	0,1	0,2	0,0	0,1	0,1	0,1	FAUX	FAUX	0,1
hsa-miR-136-3p	22	69	37	54	156	50	21	44	0,6	1,3	0,1	1,4	1,0	1,6	FAUX	FAUX	1,0
hsa-miR-598-3p	43	48	42	52	90	47	54	57	1,0	0,9	0,5	1,0	0,8	0,8	FAUX	FAUX	0,8
hsa-miR-148a-3p	61	29	52	23	34	24	41	88	1,2	1,2	1,8	1,2	1,5	0,3	FAUX	FAUX	1,2

hsa-miR-654-5p	22	61	73	66	88	109	55	77	0,3	0,9	0,3	0,6	0,4	0,8	FAUX	FAUX	0,5
hsa-miR-425-5p	20	63	133	73	279	119	83	134	0,1	0,9	0,1	0,5	0,2	0,5	FAUX	FAUX	0,4
hsa-miR-548au-5p	27	52	41	19	47	48	34	63	0,7	2,8	0,6	1,1	0,8	0,8	FAUX	FAUX	1,1
hsa-miR-410-3p	16	62	170	99	726	264	99	265	0,1	0,6	0,0	0,2	0,2	0,2	FAUX	FAUX	0,2
hsa-miR-299-3p	22	56	42	47	273	113	21	68	0,5	1,2	0,1	0,5	1,0	0,8	FAUX	FAUX	0,7
hsa-miR-195-5p	32	43	39	26	42	18	11	6	0,8	1,7	0,8	2,4	2,9	6,8	FAUX	FAUX	2,6
hsa-miR-330-3p	21	51	39	28	27	46	49	38	0,5	1,8	0,8	1,1	0,4	1,3	FAUX	FAUX	1,0
hsa-miR-548d-5p	40	32	16	26	18	20	22	25	2,4	1,2	2,2	1,6	1,8	1,3	FAUX	FAUX	1,8
hsa-miR-484	16	54	80	82	119	115	94	110	0,2	0,7	0,1	0,5	0,2	0,5	FAUX	FAUX	0,4
hsa-miR-16-2-3p	34	37	24	16	58	54	37	81	1,4	2,2	0,6	0,7	0,9	0,5	FAUX	FAUX	1,0
hsa-miR-193b-3p	32	39	67	113	69	46	20	19	0,5	0,3	0,5	0,9	1,6	2,0	FAUX	FAUX	1,0
hsa-miR-335-5p	55	13	6	0	17	0	14	14	8,5	1335,0	3,2	1335,0	3,9	1,0	FAUX	FAUX	447,8
hsa-miR-32-3p	32	37	13	9	35	15	29	23	2,4	3,9	0,9	2,4	1,1	1,6	FAUX	FAUX	2,1
hsa-miR-548c-5p	25	43	34	14	39	41	26	53	0,7	3,1	0,6	1,0	0,9	0,8	FAUX	FAUX	1,2
hsa-miR-548o-5p	25	43	34	14	39	41	26	53	0,7	3,1	0,6	1,0	0,9	0,8	FAUX	FAUX	1,2
hsa-miR-548am-5p	25	43	34	14	39	41	26	53	0,7	3,1	0,6	1,0	0,9	0,8	FAUX	FAUX	1,2
hsa-miR-29b-3p	19	49	19	52	26	19	8	20	1,0	0,9	0,7	2,5	2,4	2,4	FAUX	FAUX	1,7
hsa-miR-454-3p	36	31	266	192	204	139	211	214	0,1	0,2	0,2	0,2	0,2	0,1	FAUX	FAUX	0,2
hsa-miR-29b-1-5p	25	41	10	19	21	19	48	36	2,5	2,2	1,2	2,1	0,5	1,2	FAUX	FAUX	1,6
hsa-miR-199a-5p	26	38	16	33	29	40	17	38	1,6	1,2	0,9	0,9	1,5	1,0	FAUX	FAUX	1,2
hsa-miR-342-5p	22	41	28	73	18	17	19	36	0,8	0,6	1,2	2,5	1,2	1,2	FAUX	FAUX	1,2
hsa-miR-455-3p	21	42	149	185	230	188	116	109	0,1	0,2	0,1	0,2	0,2	0,4	FAUX	FAUX	0,2
hsa-miR-935	18	46	26	26	25	17	15	9	0,7	1,8	0,7	2,6	1,2	5,1	FAUX	FAUX	2,0
hsa-miR-339-3p	15	47	16	12	22	8	10	10	0,9	4,0	0,7	5,6	1,5	4,6	FAUX	FAUX	2,9
hsa-miR-154-5p	7	54	39	66	72	65	9	25	0,2	0,8	0,1	0,8	0,7	2,1	FAUX	FAUX	0,8
hsa-miR-146b-5p	29	32	83	40	56	157	53	39	0,4	0,8	0,5	0,2	0,6	0,8	FAUX	FAUX	0,5
hsa-miR-99a-3p	18	42	97	66	76	48	28	63	0,2	0,6	0,2	0,9	0,6	0,7	FAUX	FAUX	0,5
hsa-miR-431-5p	15	44	71	63	272	203	61	185	0,2	0,7	0,1	0,2	0,2	0,2	FAUX	FAUX	0,3
hsa-miR-548ah-3p	47	11	7	9	18	45	6	8	7,2	1,2	2,6	0,2	7,4	1,5	FAUX	FAUX	3,3
hsa-miR-548o-3p	44	11	7	9	16	46	6	10	6,8	1,2	2,8	0,2	7,0	1,1	FAUX	FAUX	3,2
hsa-miR-485-5p	15	40	11	35	18	18	20	18	1,3	1,1	0,8	2,2	0,8	2,3	FAUX	FAUX	1,4
hsa-miR-1304-3p	25	30	32	12	52	194	68	103	0,8	2,6	0,5	0,2	0,4	0,3	FAUX	FAUX	0,8
hsa-miR-625-5p	18	37	36	33	29	21	35	33	0,5	1,1	0,6	1,8	0,5	1,1	FAUX	FAUX	0,9
hsa-miR-323b-3p	9	44	28	19	139	245	58	123	0,3	2,4	0,1	0,2	0,2	0,4	FAUX	FAUX	0,6
hsa-miR-576-3p	22	31	21	9	20	14	23	16	1,1	3,3	1,1	2,3	1,0	1,9	FAUX	FAUX	1,8
hsa-miR-106b-5p	15	37	99	42	154	26	55	86	0,2	0,9	0,1	1,4	0,3	0,4	FAUX	FAUX	0,5
hsa-miR-214-3p	16	34	37	28	24	28	21	27	0,4	1,2	0,7	1,2	0,8	1,3	FAUX	FAUX	1,0
hsa-miR-452-5p	15	36	83	38	99	40	50	67	0,2	0,9	0,2	0,9	0,3	0,5	FAUX	FAUX	0,5
hsa-miR-4792	23	27	177	342	43	1	1	8	0,1	0,1	0,5	38,1	29,3	3,5	FAUX	FAUX	11,9
hsa-miR-505-3p	19	30	31	42	33	42	24	24	0,6	0,7	0,6	0,7	0,8	1,2	FAUX	FAUX	0,8
hsa-miR-450a-2-3p	22	23	11	2	12	10	8	19	2,0	9,9	1,9	2,4	2,8	1,2	FAUX	FAUX	3,4
hsa-miR-365b-5p	29	16	8	14	18	16	17	30	3,6	1,1	1,6	1,0	1,7	0,5	FAUX	FAUX	1,6
hsa-miR-127-5p	11	33	31	21	136	75	14	44	0,3	1,6	0,1	0,4	0,7	0,8	FAUX	FAUX	0,7
hsa-miR-92a-1-5p	16	26	2	14	48	17	13	56	10,1	1,8	0,3	1,5	1,2	0,5	FAUX	FAUX	2,6
hsa-miR-548i	14	27	6	19	10	25	13	30	2,2	1,4	1,3	1,1	1,1	0,9	FAUX	FAUX	1,3
hsa-miR-2682-5p	9	29	19	28	13	19	20	13	0,5	1,0	0,7	1,6	0,5	2,3	FAUX	FAUX	1,1
hsa-miR-542-5p	18	20	28	30	17	10	6	19	0,6	0,7	1,0	2,1	3,2	1,1	FAUX	FAUX	1,4
hsa-miR-23c	11	26	16	14	39	19	10	5	0,6	1,8	0,3	1,3	1,0	5,0	FAUX	FAUX	1,7
hsa-miR-30b-3p	11	26	13	9	10	10	14	20	0,8	2,7	1,0	2,6	0,7	1,3	FAUX	FAUX	1,5
hsa-miR-301b-3p	9	27	26	12	34	8	17	24	0,4	2,3	0,3	3,2	0,6	1,1	FAUX	FAUX	1,3
hsa-miR-664a-5p	8	28	8	9	4	0	2	0	1,0	3,0	2,1	2779,0	5,2	2779,0	FAUX	FAUX	928,2
hsa-miR-193a-5p	16	18	3	26	12	14	13	14	5,0	0,7	1,4	1,3	1,2	1,3	FAUX	FAUX	1,8
hsa-miR-628-5p	16	17	13	5	5	5	1	6	1,3	3,5	3,1	3,4	20,5	2,6	FAUX	FAUX	5,7
hsa-miR-769-3p	15	18	29	52	21	43	37	41	0,5	0,3	0,7	0,4	0,4	0,4	FAUX	FAUX	0,5
hsa-miR-139-3p	14	19	24	30	16	15	12	15	0,6	0,6	0,9	1,2	1,2	1,2	FAUX	FAUX	1,0
hsa-miR-215-5p	12	21	2	0	12	8	2	15	7,2	2113,0	1,0	2,8	4,9	1,4	FAUX	FAUX	355,0
hsa-miR-210-3p	13	19	67	45	72	109	39	49	0,2	0,4	0,2	0,2	0,3	0,4	FAUX	FAUX	0,3
hsa-miR-652-3p	11	20	13	14	24	14	9	10	0,8	1,4	0,4	1,5	1,1	2,0	FAUX	FAUX	1,2
hsa-miR-7706	11	20	8	12	8	9	17	27	1,3	1,7	1,3	2,2	0,6	0,8	FAUX	FAUX	1,3
hsa-miR-93-3p	7	23	5	2	12	6	4	10	1,4	9,9	0,6	4,2	1,8	2,3	FAUX	FAUX	3,4
hsa-miR-30c-1-3p	16	13	3	9	4	9	13	6	5,0	1,4	4,2	1,5	1,2	2,1	FAUX	FAUX	2,6
hsa-miR-30d-3p	13	17	13	12	21	12	6	9	1,0	1,4	0,6	1,4	2,0	1,9	FAUX	FAUX	1,4
hsa-miR-3200-3p	7	22	28	33	16	56	15	14	0,3	0,7	0,4	0,4	0,5	1,6	FAUX	FAUX	0,6
hsa-miR-548av-5p	20	9	3	16	9	17	12	10	6,1	0,5	2,2	0,5	1,7	0,9	FAUX	FAUX	2,0
hsa-miR-548k	20	9	3	16	9	17	12	10	6,1	0,5	2,2	0,5	1,7	0,9	FAUX	FAUX	2,0
hsa-miR-22-5p	11	18	23	12	37	12	7	11	0,5	1,5	0,3	1,5	1,5	1,6	FAUX	FAUX	1,1
hsa-miR-6511b-5p	21	7	29	28	8	4	9	61	0,7	0,2	2,7	1,6	2,2	0,1	FAUX	FAUX	1,3
hsa-miR-671-3p	13	14	11	9	22	66	46	57	1,1	1,5	0,6	0,2	0,3	0,3	FAUX	FAUX	0,7
hsa-miR-2110	13	12	5	14	5	9	10	11	2,6	0,9	2,5	1,4	1,3	1,1	FAUX	FAUX	1,6
hsa-miR-1268a	12	11	7	0	8	6	7	18	1,8	1113,0	1,5	2,0	1,6	0,6	FAUX	FAUX	186,8
hsa-miR-505-5p	9	13	8	7	3	2	1	5	1,2	1,9	3,6	6,4	11,7	2,6	FAUX	FAUX	4,6
hsa-miR-7641	9	13	65	61	170	36	129	129	0,1	0,2	0,1	0,4	0,1	0,1	FAUX	FAUX	0,2
hsa-miR-671-5p	8	14	50	30	58	74	64	91	0,2	0,5	0,1	0,2	0,1	0,2	FAUX	FAUX	0,2
hsa-miR-758-3p	8	14	18	19	54	37	17	20	0,5	0,8	0,2	0,4	0,5	0,7	FAUX	FAUX	0,5
hsa-miR-3679-5p	7	16	11	9	3	3	8	10	0,6	1,7	2,7	4,5	0,9	1,5	FAUX	FAUX	2,0
hsa-miR-424-5p	13	9	23	21	43	4	8	20	0,6	0,4	0,3	2,1	1,6	0,4	FAUX	FAUX	0,9
hsa-miR-374a-3p	11	11	42	14	177	31	35	91	0,2	0,8	0,1	0,4	0,3	0,1	FAUX	FAUX	0,3
hsa-miR-548ap-5p	8	13	11	12	12	14	10	5	0,7	1,1	0,7	0,9	0,8	2,6	FAUX	FAUX	1,2
hsa-miR-548j-5p	8	13	11	12	10	13	9	5	0,7	1,1	0,8	1,0	0,9	2,6	FAUX	FAUX	1,2
hsa-miR-4645-3p	9	11	13	2	10	11	9	6	0,7	4,7	0,9	1,0	1,1	1,8	FAUX	FAUX	1,7
hsa-miR-181b-2-3p	8	12	2	2	20	2	6	10	5,0	5,2	0,4	5,9	1,3	1,2	FAUX	FAUX	3,2
hsa-miR-378d	7	13	7	0	34	24	24	36	1,1	1335,0	0,2	0,6	0,3	0,4	FAUX	FAUX	222,9
hsa-miR-483-5p	12	8	0	9	3	1	0	3	1171,0	0,8	4,5	11,1	1171,0	3,1	FAUX	FAUX	393,6
hsa-miR-1197	9	10	15	9	5	3	6	15	0,6	1,1	1,8	2,9	1,7	0,7	FAUX	FAUX	1,5
hsa-miR-1260b	11	8	294	66	375	192	280	351	0,0	0,1	0,0	0,0	0,0	0,0	FAUX	FAUX	0,0
hsa-miR-548e-5p	11	8	3	5	10	1	3	1	3,2	1,7	1,0	11,1	3,3	6,1	FAUX	FAUX	4,4
hsa-miR-454-5p	9	9	3	7	14	12	9	34	2,9	1,3	0,7	0,8	1,0	0,3	FAUX	FAUX	1,1
hsa-miR-181b-3p	8	10	3	0	26	5	7	22	2,5	1001,0	0,3	2,1	1,2	0,5	FAUX	FAUX	167,9
hsa-miR-1254	7	11	0	23	3	8	13	14	703,0	0,5	2,7	1,3	0,6	0,8	FAUX	FAUX	118,1
hsa-miR-329-3p	7	11	16	23	58	48	8	20	0,4	0,5	0,1	0,2	0,9	0,5	FAUX	FAUX	0,4
hsa-miR-548l	9	8	3	9	9	10	14	6	2,9	0,8	1,0	0,8	0,7	1,2	FAUX	FAUX	1,2
hsa-miR-216b-5p	8	9	3	7	5	3	6	15	2,5	1,3	1,6	3,2	1,3	0,6	FAUX	FAUX	1,7
hsa-miR-1179	7	10	0	5	8	1	5	6	703,0	2,1	0,9	7,2	1,5	1,6	FAUX	FAUX	119,4
hsa-miR-1260a	9	7	291	59	368	187	276	351	0,0	0,1	0,0	0,0	0,0	0,0	FAUX	FAUX	0,0
hsa-miR-548b-5p	9	7	3	0	4	3	8	5	2,9	668,0	2,4	1,9	1,2	1,3	FAUX	FAUX	112,9
hsa-miR-5187-5p	8	7	2	5	0	1	0	3	5,0	1,4	820,0	9,5	820,0	2,6	FAUX	FAUX	276,4

Supplementary Table 2a. Associations between recipient, donor and procedure characteristics on peritubular capillary density on the post-transplant biopsy in univariable analyses.

Recipient/ Donor/ Procedure characteristics	Difference (95 % CI) in %	p-value
miR-423-5p levels (per 1 natural log higher)	0.8 (0.2, 1.4)	0.01
Recipient age	-0.2 (-0.6, 0.3)	0.53
Recipient female sex	0.5 (-0.7, 1.7)	0.41
Recipient African American race	-1.3 (-3.0, 0.4)	0.13
Cause of chronic kidney disease		
Other (reference)		
Diabetes	0.4 (-1.3, 2.1)	0.60
Autoimmune disease	0.2 (-2.3, 2.7)	0.88
Hypertension/vascular	0.2 (-1.8, 2.2)	0.85
Glomerular disease	0.4 (-0.9, 1.6)	0.58
Time on dialysis pre-transplant (per 1-month higher)	0.0 (0.0, 0.0)	0.85
Pre-emptive transplantation	1.3 (-0.7, 3.4)	0.20
Previous transplantations	-0.5 (-2.0, 1.0)	0.49
Recipient diabetes	0.5 (-0.8, 1.7)	0.48
Recipient coronary artery disease	-0.2 (-2.0, 1.5)	0.78
Recipient body mass index (per 1 unit higher)	0.0 (-0.1, 0.1)	0.47
Recipient active smoking at transplantation	0.5 (-1.0, 2.1)	0.48
Recipient statin use at transplantation	1.0 (-0.1, 2.2)	0.06
Recipient statin use 6 months post transplantation	0.3 (-0.8, 1.4)	0.58
Recipient RAS blockers use at transplantation	-0.6 (-1.8, 0.7)	0.35
Recipient RAS blockers use 6 months post transplantation	0.1 (-1.3, 1.6)	0.85
Pre-transplant PRA > 0% (versus 0%)	-0.1 (-1.6, 1.5)	0.92
Peak historical PRA > 0% (versus 0%)	-0.5 (-1.6, 0.7)	0.42
HLA mismatches (reference 0-2)		
3-4	0.6 (-3.6, 4.8)	0.77
5-6	0.9 (-3.4, 5.2)	0.67
Thymoglobulin induction	-0.1 (-1.3, 1.0)	0.81
Total ischemic time (per 1-hour higher)	0.0 (-0.1, 0.1)	0.86
Donor type (reference neurologically deceased)		
Living donor	-0.6 (-3.0, 1.8)	0.61
Donor after cardiac arrest	0.8 (-0.6, 2.2)	0.25
Use of hypothermic machine perfusion	1.1 (0.0, 2.2)	0.06
Donor age (per 10-year higher)	-0.1 (-0.5, 0.3)	0.53
Donor creatinine (per 10 µmol/L higher)	0.1 (-0.1, 0.3)	0.33
Female donor sex (versus male)	-0.5 (-1.6, 0.6)	0.39

Donor positive smoking history (versus negative or unknown)	0.3 (-0.9, 1.4)	0.65
Donor peripheral vascular disease	1.0 (-1.4, 3.4)	0.41
Donor hypertension	-0.7 (-2.1, 0.8)	0.37
Donor diabetes	-1.1 (-3.1, 1.0)	0.30
Time to biopsy in days	0.0 (0.0, 0.0)	0.17
Rejection before the post-transplant biopsy	0.2 (-1.0, 1.4)	0.69
Peritubular capillary density on the pre-transplant biopsy (per 1% higher)	0.1 (0.0, 0.3)	0.12

Supplementary Table 2b. Associations between recipient, donor and procedure characteristics on peritubular capillary density on the post-transplant biopsy in the initial multivariable model

Clinical characteristics	Adjusted difference in %, 95% CI	p-value
miR-423-5p levels 1 month post-transplant (per 1 natural log higher)	1.0 (0.4, 1.5)	0.001
Female recipient sex (reference male)	1.2 (-0.1, 2.4)	0.06
Peritubular capillary density on the pre-implantation biopsy (per 1% higher)	0.1 (-0.0, 0.3)	0.11
Use of hypothermic pump during transportation	1.0 (-0.0, 2.0)	0.06
Use of thymoglobulin as induction	0.5 (-0.7, 1.7)	0.42
African American ethnicity	-0.1 (-1.8, 1.6)	0.89
Use of statins at transplant	1.0 (0.0, 2.0)	0.04

Supplementary Table 3a. Associations between recipient, donor and procedure characteristics on interstitial fibrosis on the post-transplant biopsy in univariable analyses.

Recipient/ Donor/ Procedure characteristics	Difference (95 % CI) in %	p-value
miR-423-5p levels 1 month post transplant (per 1 natural log higher)	-4.8 (-8-8, -0.9)	0.02
Recipient age	-0.1 (-0.3, 0.1)	0.40
Recipient female sex	1.2 (-3.9, 6.3)	0.64
Recipient African American race	6.6 (-0.3, 13.5)	0.06
Cause of chronic kidney disease		
Other (reference)		
Diabetes	-5.0 (-11.6, 1.7)	0.14
Autoimmune disease	-5.4 (-15.5, 4.8)	0.29
Hypertension/vascular	-0.5 (-8.5, 7.5)	0.90
Glomerular disease	0.7 (-4.7, 6.1)	0.79
Time on dialysis pre-transplant (per 1-month higher)	0.1 (0.0, 0.1)	0.04
Pre-emptive transplantation	-2.6 (-11.2, 6.0)	0.55
Previous transplantations	2.6 (-4.1, 9.4)	0.43
Recipient diabetes	-3.8 (-9.2, 1.6)	0.16
Recipient coronary artery disease	0.9 (-6.3, 8.1)	0.80
Recipient body mass index (per 1 unit higher)	-0.15 (-0.6, 0.3)	0.51
Recipient active smoking at transplantation	1.8 (-4.6, 8.2)	0.57
Recipient statin use at transplantation	-1.9 (-6.8, 2.9)	0.42
Recipient statin use 6 months post transplantation	-3.2 (-7.8, 1.5)	0.17
Recipient RAS blockers use at transplantation	1.5 (-3.6, 6.6)	0.56
Recipient RAS blockers use 6 months post transplantation	-0.5 (-6.6, 5.6)	0.88
Pre-transplant PRA > 0% (versus 0%)	-3.7 (-10.0, 2.6)	0.25
Peak historical PRA > 0% (versus 0%)	1.5 (-3.2, 6.3)	0.52
HLA mismatches (reference 0-2)		
3-4	1.6 (-14.6, 17.7)	0.85
5-6	7.1 (-9.5, 23.7)	0.40
Thymoglobulin induction	-0.8 (-5.6, 4.0)	0.74
Total ischemic time (per 1-hour higher)	0.07 (-0.53, 0.40)	0.77
Donor type (reference neurologically deceased)		
Living donor	0.6 (-9.3, 10.5)	0.90
Donor after cardiac arrest	-3.4 (-9.3, 2.4)	0.25
Use of hypothermic machine perfusion	4.4 (-0.3, 9.1)	0.07
Donor age (per 10-year higher)	0.2 (0.1, 0.3)	0.01
Donor creatinine (per 10 µmol/L higher)	0.0 (-0.07, 0.1)	0.93
Female donor sex (versus male)	-1.8 (-6.5, 2.9)	0.45

Donor positive smoking history (versus negative or unknown)	2.5 (-2.2, 7.3)	0.28
Donor peripheral vascular disease	-2.7 (-12.5, 7.1)	0.58
Donor hypertension	4.0 (-2.0, 10.0)	0.18
Donor diabetes	1.2 (-7.4, 9.8)	0.78
Time to biopsy in days	-0.1 (-0.2, 0.1)	0.31
Rejection before the post-transplant biopsy	6.8 (-1.1, 14.7)	0.08
Peritubular capillary density on the pre-transplant biopsy (per 1% higher)	0.2 (-0.4, 0.8)	0.44
Fibrosis on the pre-implantation biopsy (per 1% higher)	0.6 (0.1, 1.1)	0.03

RAS: renin-angiotensin system; PRA: panel reactive antibodies

Supplementary Table 3b. Associations between recipient, donor and procedure characteristics on interstitial fibrosis on the post-transplant biopsy in the initial multivariable model

Clinical characteristics	Adjusted difference in %, 95% CI	p-value
miR-423-5p levels 1 month post-transplant (per 1 natural log higher)	-4.6 (-8.6, -0.9)	0.02
Female recipient sex (reference male)	4.8 (-3.1, 12.7)	0.23
Time on dialysis before transplantation (per 1 month higher)	0.1 (-0.0, 0.2)	0.09
Donor age (per 10 year higher)	3.3 (0.8, 5.8)	0.01
Rejection before/on the post-transplant biopsy	7.0 (-0.2, 14.3)	0.056
African American ethnicity	-4.6 (-16.7, 7.5)	0.45
Thymoglobulin as induction	0.6 (-7.9, 9.1)	0.89
Use of hypothermic pump during organ transportation	1.1 (-6.3, 8.5)	0.76
Fibrosis on the pre-implantation biopsy (per 1% higher)	-0.0 (-0.1, 0.1)	0.98

Figure S1. Apoptotic exosome-like vesicles (ApoExos) from endothelial cells, but not from renal tubular epithelial cells, are enriched in miR-423-5p. (**A**) Representative immunoblots of CD82, SDCBP, LG3 and PSMA3 in large (centrifugation at 50,000 xg) and small (centrifugation at 200,000 xg) EV fractions purified from endothelial cells under normal condition for 4 h. (**B**) Evaluation by Hoescht 33342 and Propidium iodide (HO/PI) staining of apoptosis in renal tubular epithelial cells serum-starved (SS) for 48 h. P value was obtained by one sample t test (*** $P < 0.001$), $n = 9$. Representative immunoblots of SDCBP, PSMA3, LG3, and histone H3C1 in large (centrifugation at 50,000 xg) and small (centrifugation at 200,000 xg) EV fractions purified from apoptotic renal tubular epithelial cells serum starved for 48 h. (**C**) MiR-423-5p, let-7b-5p and let-7c-5p expression in large and small EV fractions derived from apoptotic endothelial cells or apoptotic renal tubular epithelial cells. Expression of miRNAs was measured by quantitative RT-PCR and the result is presented as relative copies expression of miRNA per ng of RNA ± SEM after normalization with cel-miR-39; $n = 3$. P values were obtained by one-way ANOVA and Bonferroni post hoc test (* $P < 0.05$, ** $P < 0.01$).

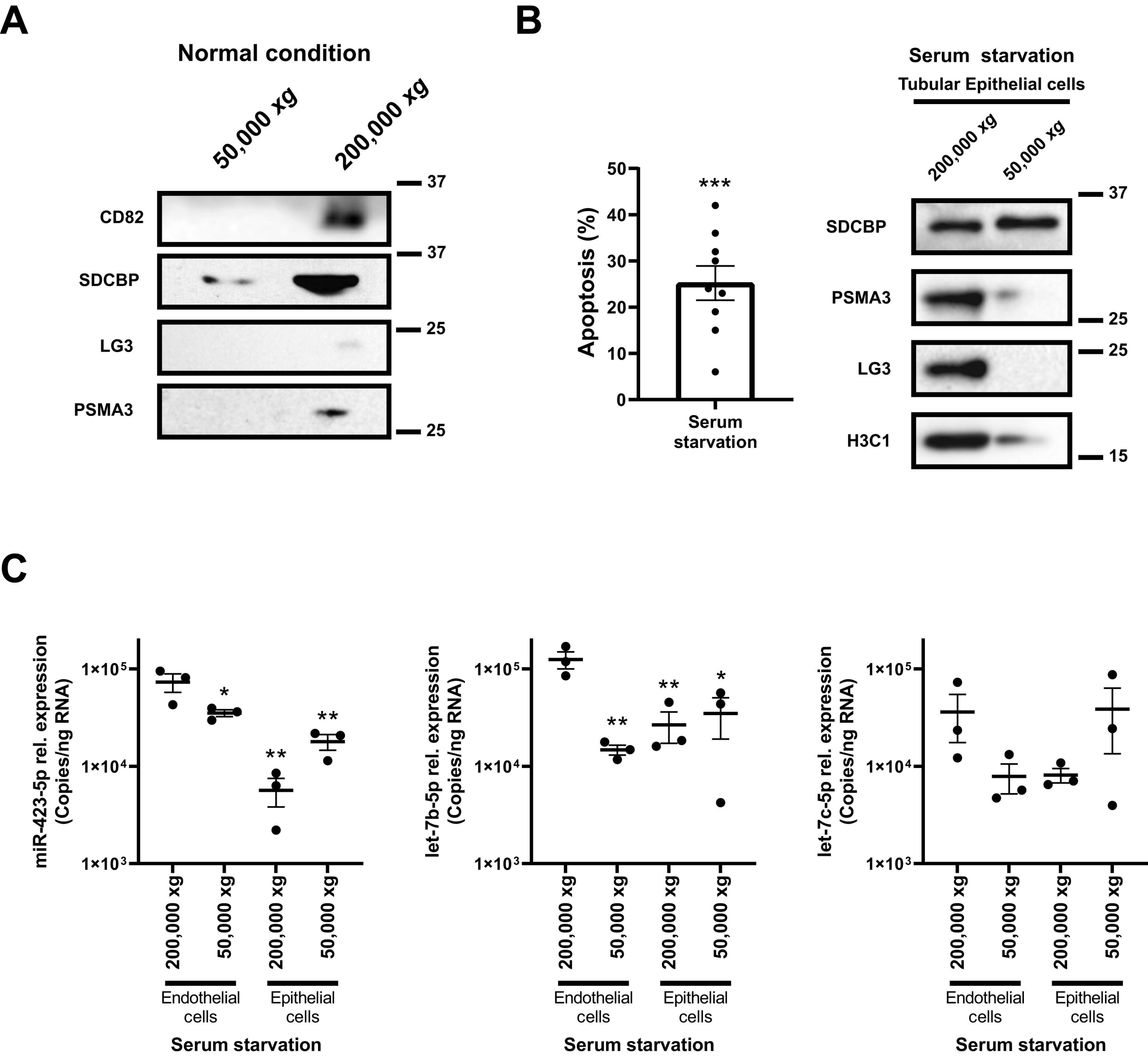

A
Normal condition
50,000 xg
200,000 xg
CD82
SDCBP
LG3
PSMA3
37
37
25
25
B
Apoptosis (%)
***
Serum starvation
Serum starvation
Tubular Epithelial cells
200,000 xg
50,000 xg
SDCBP
PSMA3
LG3
H3C1
37
25
25
15
C
miR-423-5p rel. expression (Copies/ng RNA)
let-7b-5p rel. expression (Copies/ng RNA)
let-7c-5p rel. expression (Copies/ng RNA)
1×10⁵
1×10⁴
1×10³
200,000 xg
50,000 xg
Endothelial cells
Epithelial cells
Serum starvation

Figure S2. Circulating miR-423-5p, let-7b-5p and let-7c-5p in mice after renal IRI. **(A)** Quantification of let-7c-5p serum levels presurgery or 2, 7 and 21 days post IRI. Let-7c-5p was measured by quantitative RT–PCR, and the result is presented as relative copy expression of miRNA per µL of total serum ± SEM after normalization with cel-miR-39; n ≥ 5. Values are the mean ± SEM. P values were obtained by one-way ANOVA and Bonferroni post hoc test. (**B**) Correlation matrix of miRNAs mouse serum levels. P values were obtained by Spearman correlation and *r* is represented by heatmap. (**C**) Quantification of miR-361-5p serum levels presurgery or 2, 7 and 21 days post IRI. MiR-361-5p was measured by quantitative RT–PCR, and the results are presented as relative copy expression of miRNA per µL of total serum ± SEM after normalization with cel-miR-39; n ≥ 4. Values are the mean ± SEM. (**D**) Quantification of let-7c-5p serum levels in wild-type mice at baseline. Serum was treated or not with RNase A (0.025 mg/mL) with or without Triton X-100 (0.1 %) for 20 min at 37 °C. Expression of let-7c-5p was measured by quantitative RT-PCR and the result is presented as relative copies of miRNA per µL of total serum ± SEM. n = 3 for each condition. P values were obtained by one-way ANOVA and Tukey post hoc test (****, P < 0.0001).

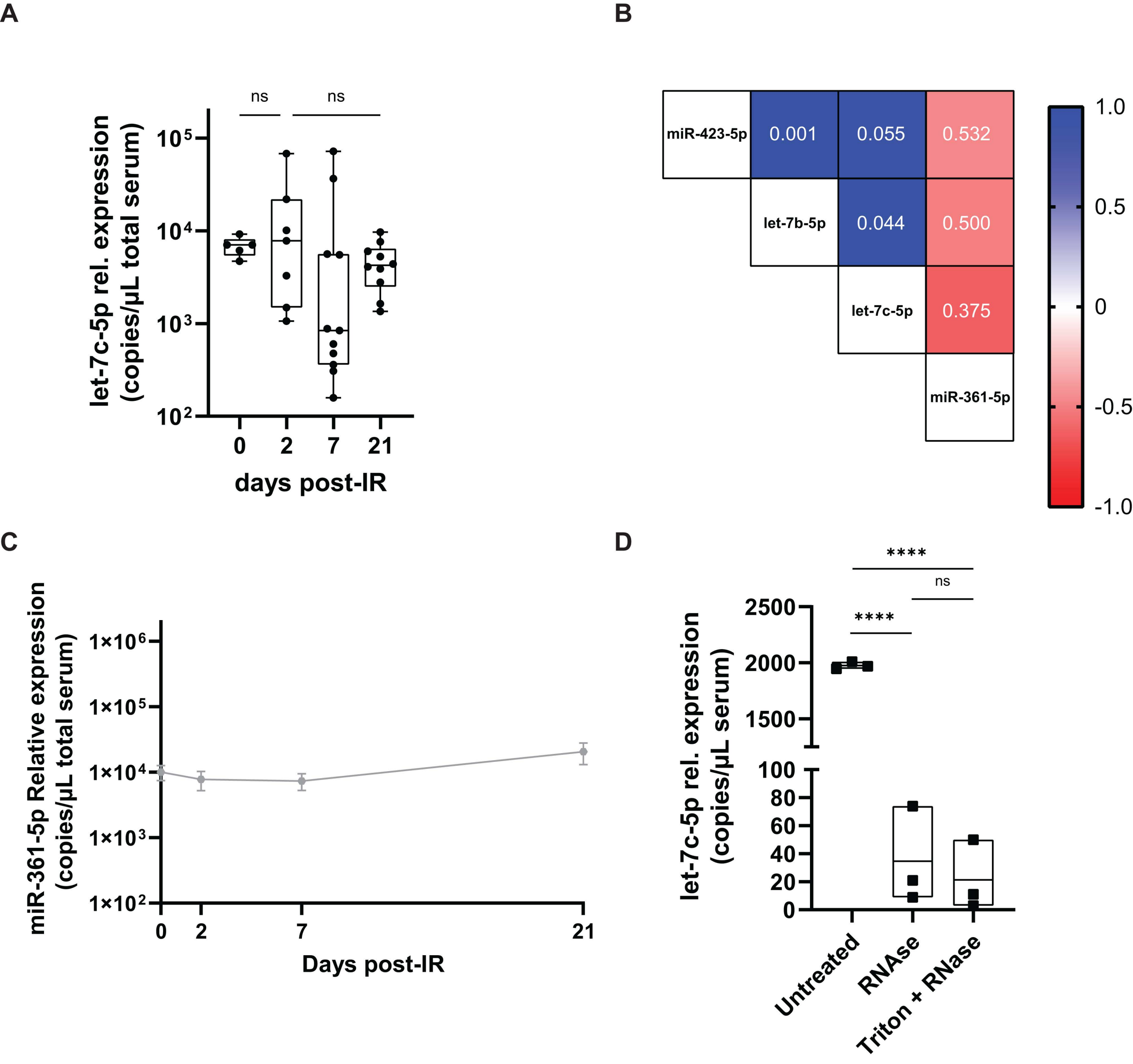

A
ns
ns
let-7c-5p rel. expression
(copies/µL total serum)
10⁵
10⁴
10³
10²
0
2
7
21
days post-IR
B
miR-423-5p
0.001
0.055
0.532
let-7b-5p
0.044
0.500
let-7c-5p
0.375
miR-361-5p
1.0
0.5
0
-0.5
-1.0
C
miR-361-5p Relative expression
(copies/µL total serum)
1×10⁶
1×10⁵
1×10⁴
1×10³
1×10²
0
2
7
21
Days post-IR
D
****
ns
****
let-7c-5p rel. expression
(copies/µL serum)
2500
2000
1500
100
80
60
40
20
0
Untreated
RNAse
Triton + RNase

Figure S3. At a distance from IRI, lower miR-423-5p serum levels correlate with more severe microvascular rarefaction and renal fibrosis. (**A**) Blood urea nitrogen (BUN) concentration in mice at baseline or 1, 2, 7, or 21 days after 30-min (IR30) and 60-min (IR60) renal ischemia–reperfusion injury (IRI); n = 5 for each condition. (**B**) Quantification of miR-423-5p serum levels presurgery or 1, 2, 7 and 21 days post IRI. MiR-423-5p was measured by quantitative RT–PCR, and the result is presented as relative copy expression of miRNA per µL of total serum ± SEM after normalization with cel-miR-39; n ≥ 6. Values are the mean ± SEM. (**C**) Quantification of cleaved caspase-3-positive peritubular capillaries (PTCs) in renal sections at baseline, 1 and 2 days post-surgery in mice that underwent 30 min or 60 min or renal artery clamping; n ≥ 3. Representative images of cleaved caspase-3 immunohistochemistry in renal sections (corticomedullary junction) presurgery and at 1 day post-IRI. Scale bar: 50 µm (**D**) Quantification of phosphorylated Receptor-interacting serine/threonine-protein kinase 3 (RIPK3)-positive peritubular capillaries (PTCs) in renal sections at baseline, 1 and 2 days post-surgery in mice that underwent 30 min or 60 min of renal artery clamping; n ≥ 3. Representative images of pRIPK3 immunohistochemistry in renal sections (corticomedullary junction) at baseline and 1 day post-IRI. P values compared to baseline (time 0) [*] or between IR30 and IR60 [#]. Scale bar: 50 µm (**E**) Mean tubular injury scores of ten randomly chosen high-power fields in renal cortical sections at baseline and 2 days post-IRI in mice that underwent 30 min and 60 min IRI or 2 days post-sham operation with contralateral nephrectomy. Representative haematoxylin and eosin (H&E) stained renal sections showing tubular injury. n = 3-6 for each condition. Scale bar: 50 µm (**F**) Quantification of miR-423-5p serum levels at baseline, 2 days after the sham operation with contralateral nephrectomy, and at 2 and 21 days following the sham operation without contralateral nephrectomy. The expression of miR-423-5p was measured by quantitative RT–PCR, and the result is presented as relative copies of miR-423-5p per µL of total serum ± SEM after normalization with cel-miR-39; n ≥ 3. (**G**) Representative

immunoblots of different protein markers in large (centrifugation at 50,000 xg) and small (centrifugation at 200,000 xg) extracellular vesicles purified from mouse serum at baseline and 2 days post-sham operation with contralateral nephrectomy. PLVAP is a marker of endothelial-derived extracellular vesicles, PSMA3 and LG3 are markers of apoptotic exosome-like vesicles, ACTB is a general marker of extracellular vesicles and CD82 is an exosome marker, n = 3. All P values were obtained by one-way ANOVA and the Bonferroni post hoc test (* $P < 0.05$, ** $P < 0.01$, *** $P < 0.001$, **** $P < 0.0001$).

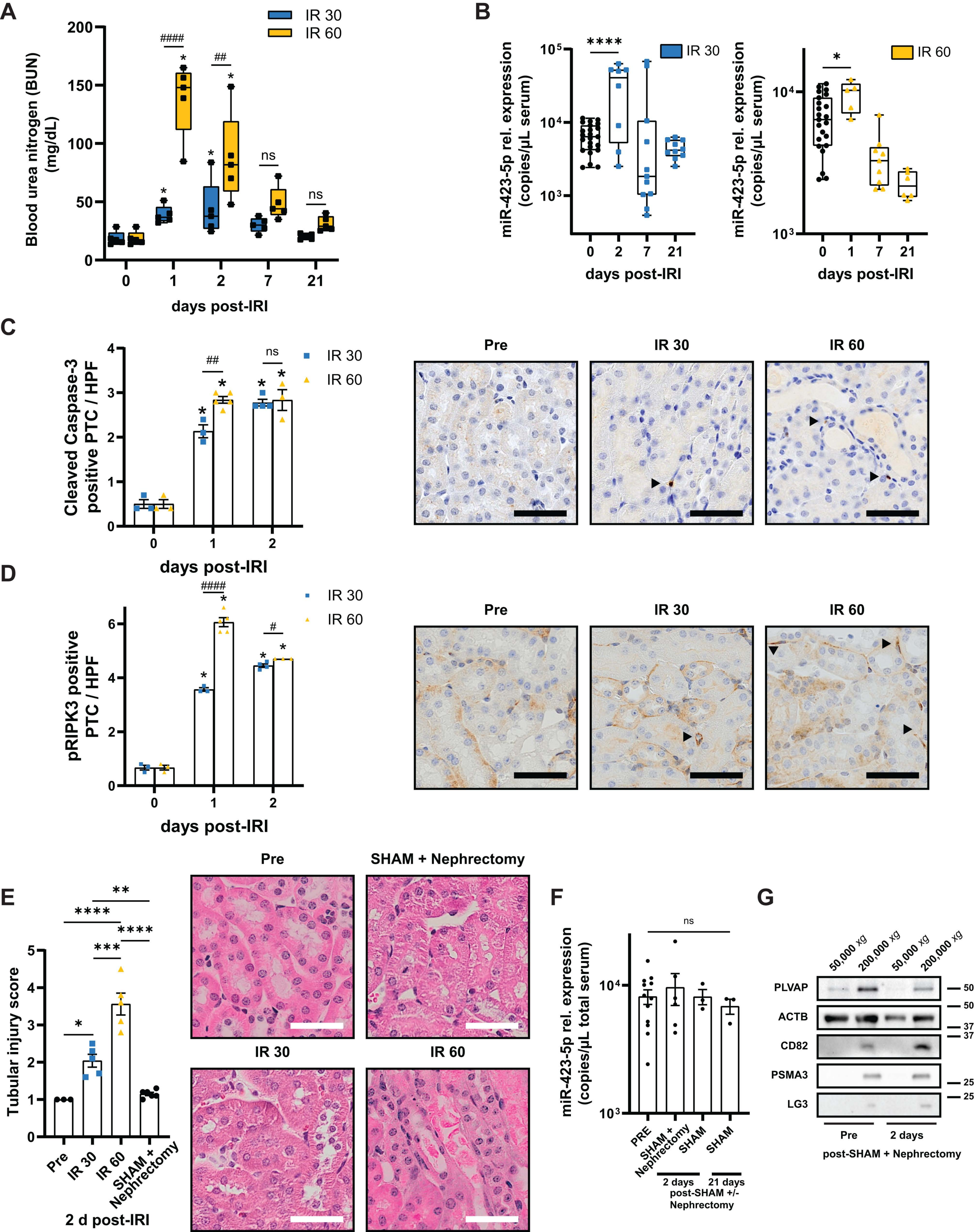
A
IR 30
IR 60
Blood urea nitrogen (BUN) (mg/dL)
days post-IRI
B
miR-423-5p rel. expression (copies/µL serum)
days post-IRI
C
Cleaved Caspase-3 positive PTC / HPF
Pre
IR 30
IR 60
days post-IRI
D
pRIPK3 positive PTC / HPF
E
Tubular injury score
Pre
IR 30
IR 60
SHAM + Nephrectomy
2 d post-IRI
F
miR-423-5p rel. expression (copies/µL total serum)
PRE
SHAM
2 days
21 days
post-SHAM +/- Nephrectomy
G
50,000 xg
200,000 xg
PLVAP
ACTB
CD82
PSMA3
LG3
2 days
post-SHAM + Nephrectomy

Figure S4. Serum levels of miR-423-5p 21 days after renal ischemia-reperfusion injury (IRI) are independent of caspase-3 activation. (**A**) Kaplan-Meier survival curves of wild-type (WT) or caspase-3 knockout (Casp-3 -/-) mice that underwent 30 min or 60 min of renal artery clamping; n ≥ 23. P values were obtained by log-rank test (** P < 0.01). (**B**) Quantification of miR-423-5p serum levels 21 days after 60-min renal artery clamping in WT or Casp-3 -/- mice. Expression of miR-423-5p was measured by quantitative RT-PCR and the result is presented as relative copies of miR-423-5p per µL of total serum ± SEM after normalization with cel-miR-39. (**C**) Quantification of PLVAP-positive peritubular capillaries (PTC) per tubule with representative images of PLVAP immunohistochemistry in renal sections (corticomedullary junction) 21 days after 60 of renal artery clamping in WT or Casp-3 -/- mice. Scale bar: 50 µm (**D**) Quantification of Sirius Red within PTC areas in the corticomedullary junction of renal sections in WT or Casp-3 -/- mice 21 days after renal artery clamping for 60 min IRI with representative images of Sirius Red staining; n = 5. P values were obtained by unpaired t test (* P < 0.05, ** P < 0.01, *** P < 0.001, **** P < 0.0001). Scale bar: 50 µm (**E**) *Left*: Evaluation by Hoescht 33342 and Propidium iodide (HO/PI) staining of apoptosis in HUVECs in normal or serum-starved condition and treated with the pan-caspase inhibitor zVAD-Fmk (ZVAD) or vehicle (Ctrl) for 4 h. P values were obtained by one-way ANOVA and the Bonferroni post hoc test (** P < 0.01, **** P < 0.0001), n = 3. *Right*: MiR-423-5p expression in small extracellular vesicle fractions derived from apoptotic endothelial cells (ApoExo) or in large extracellular vesicle fractions derived from healthy endothelial cells (MVs) treated with ZVAD or its vehicle (Ctrl) for 4 h. The expression of miRNAs was measured by quantitative RT–PCR, and the results are presented as the relative copy expression of miRNA per mL of supernatant ± SEM after normalization to cel-miR-39; n = 3. P values were obtained by unpaired t test (* P < 0.05).

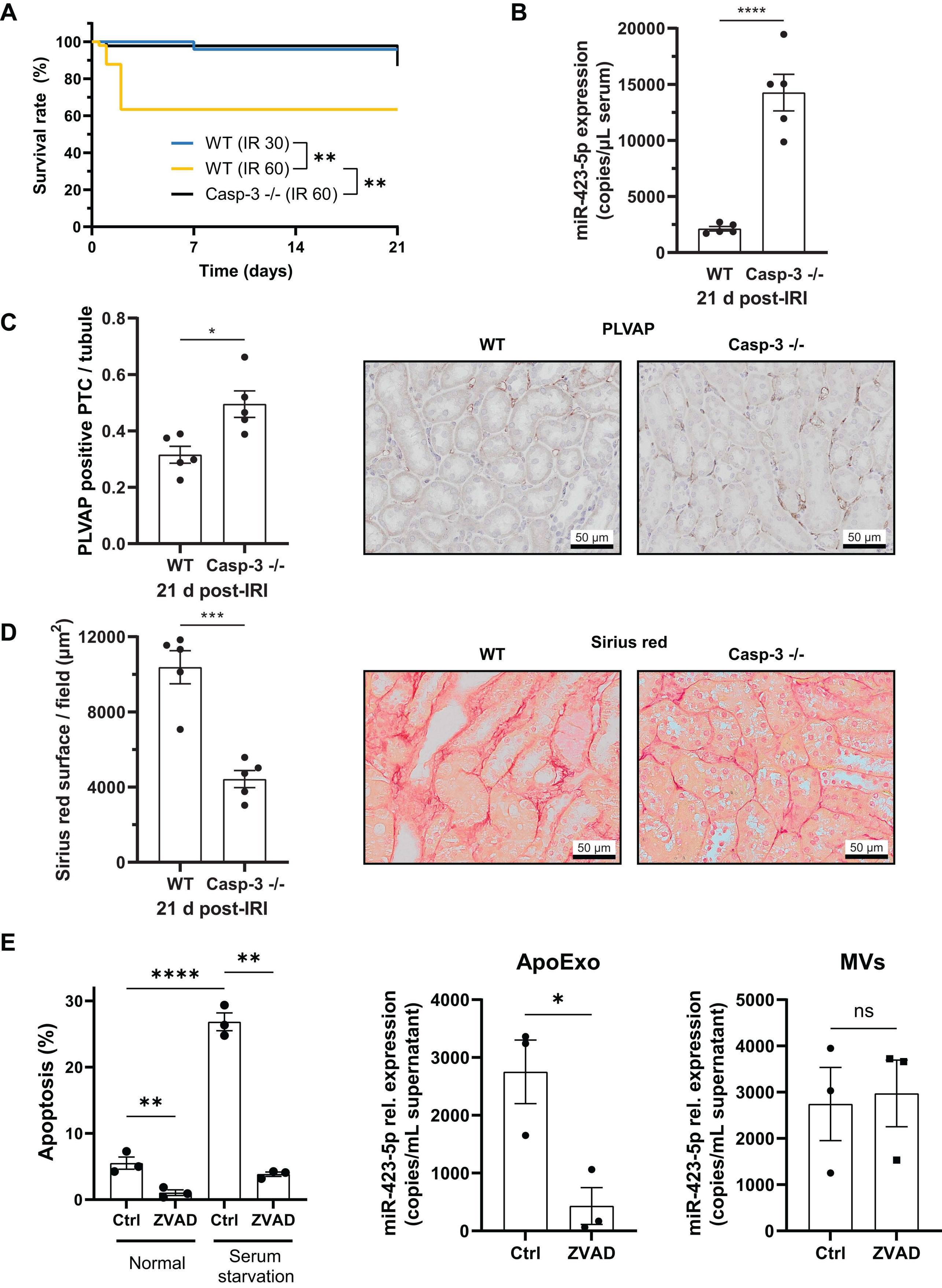

A
Survival rate (%)
100
80
60
40
20
0
0
7
14
21
Time (days)
WT (IR 30)
WT (IR 60)
Casp-3 -/- (IR 60)
**
**
B
****
miR-423-5p expression (copies/µL serum)
20000
15000
10000
5000
0
WT
Casp-3 -/-
21 d post-IRI
C
PLVAP positive PTC / tubule
0.8
0.6
0.4
0.2
0.0
*
WT
Casp-3 -/-
21 d post-IRI
PLVAP
WT
Casp-3 -/-
50 µm
50 µm
D
Sirius red surface / field (µm²)
12000
8000
4000
0
***
WT
Casp-3 -/-
21 d post-IRI
Sirius red
WT
Casp-3 -/-
50 µm
50 µm
E
Apoptosis (%)
30
20
10
0
****
**
**
Ctrl
ZVAD
Ctrl
ZVAD
Normal
Serum starvation
ApoExo
miR-423-5p rel. expression (copies/mL supernatant)
4000
3000
2000
1000
0
*
Ctrl
ZVAD
MVs
miR-423-5p rel. expression (copies/mL supernatant)
5000
4000
3000
2000
1000
0
ns
Ctrl
ZVAD

Figure S5. MiR-423-5p serum levels in human renal transplant patients. (**A**) The correlation matrix of miRNAs serum levels, measured in total serum from transplanted patients, was analyzed. P values were obtained by Spearman correlation, and the correlation coefficient (r) is represented by a heatmap. (**B**) Expression levels of miR-423-5p, let-7b-5p and let-7c-5p in total serum and extracellular vesicles purified from the same amount of patients' serum; n = 5-6. (**C**) Distribution of miR-423-5p in natural logarithm among the delayed graft function (DGF) patients; n = 51. (**D**) Relative distribution of miR-423-5p expression in large EV fraction (centrifugation at 50,000 xg) and small EV fraction (centrifugation at 200,000 xg) from the serum of six randomly chosen DGF patients at 8-10 days and one-month post-transplantation; n = 6. MiR-423-5p serum level was measured by quantitative RT-PCR and normalized with cel-miR-39. P values were obtained by paired t test (* P < 0.05).

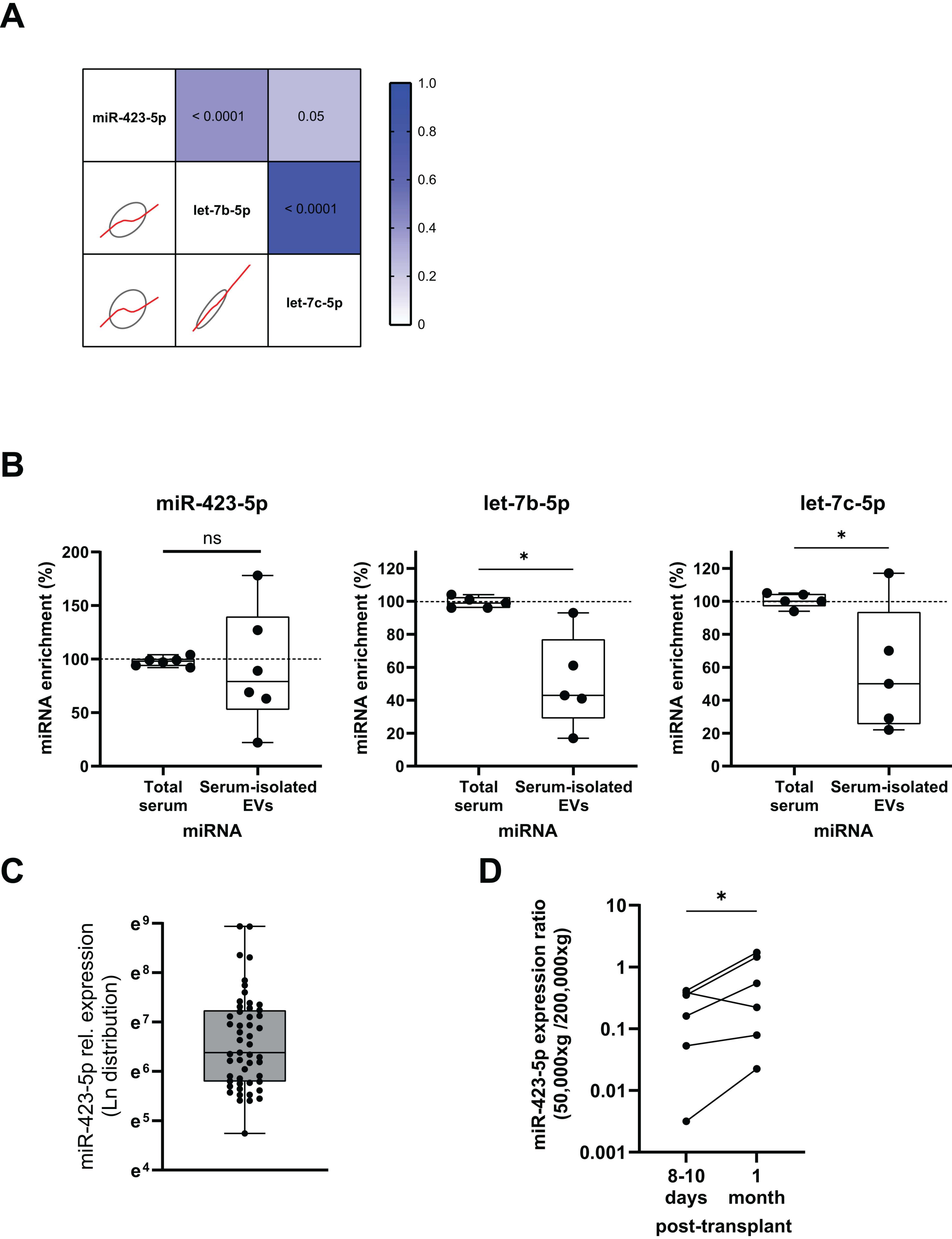
A
miR-423-5p
< 0.0001
0.05
let-7b-5p
< 0.0001
let-7c-5p
1.0
0.8
0.6
0.4
0.2
0
B
miR-423-5p
ns
miRNA enrichment (%)
200
150
100
50
0
Total serum
Serum-isolated EVs
miRNA
let-7b-5p
*
miRNA enrichment (%)
120
100
80
60
40
20
0
Total serum
Serum-isolated EVs
miRNA
let-7c-5p
*
miRNA enrichment (%)
120
100
80
60
40
20
0
Total serum
Serum-isolated EVs
miRNA
C
miR-423-5p rel. expression (Ln distribution)
e9
e8
e7
e6
e5
e4
D
*
miR-423-5p expression ratio (50,000xg /200,000xg)
10
1
0.1
0.01
0.001
8-10 days
1 month
post-transplant

Figure S6. Subcapsular injection of miR-423-5p increases renal expression levels. (**A**) Total (*Left*) or sectioned (*Right*) renal expression of miR-423-5p following subcapsular injection of Ctrl or miR-423-5p mimics 2 days post-surgery. Expression of miR-423-5p in the whole kidney (Left) or in the four kidney sections (Q1-4) (*Right*), was measured by quantitative RT-PCR and the result is presented as expression fold change ± SEM. Each quadrant corresponds to a portion of the kidney obtained through cross-sectional cuts. P value was obtained by unpaired t test (*Left*) and by one-way ANOVA and the Bonferroni post hoc test (* $P < 0.05$, ** $P < 0.01$, *** $P < 0.001$), $n \geq 4$. (**B**) Blood urea nitrogen (BUN) concentration at baseline or 2, 7, 14 and 21 days after 30-min renal ischemia–reperfusion injury (IRI) in mice with subcapsular injection of Ctrl or miR-423-5p mimics. P value was obtained by one-way ANOVA and the Bonferroni post hoc test (* $P < 0.05$); $n \geq 4$ for each condition. (**C**) Mean tubular injury scores of ten randomly chosen high-power fields in mice renal cortical sections at 2 days post-IRI from mice that underwent 30 min IRI with subcapsular injection of Ctrl or miR-423-5p mimics. P value was obtained by unpaired t test; n = 4-6 for each condition.

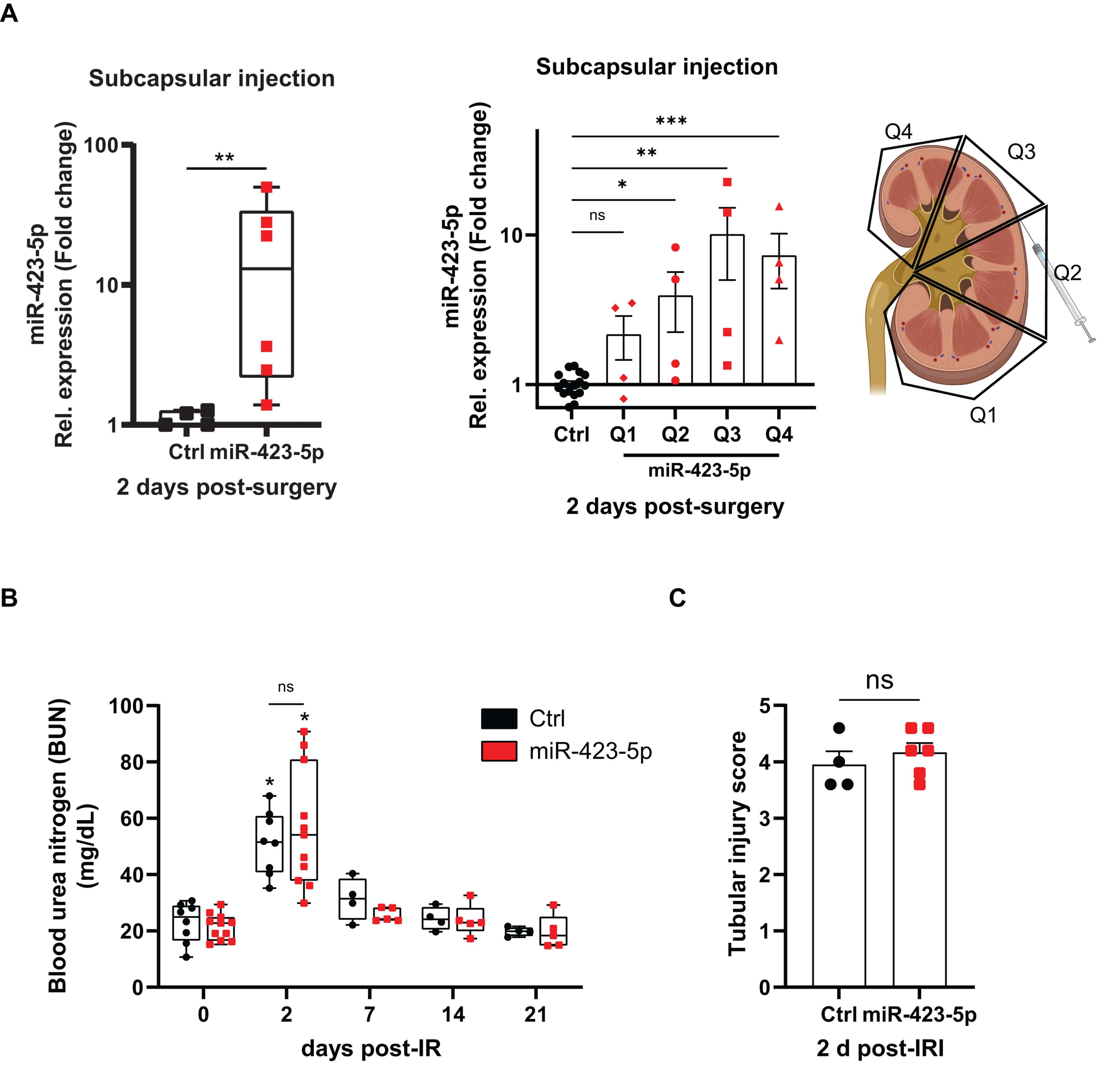

A
Subcapsular injection
miR-423-5p
Rel. expression (Fold change)
100
10
1
**
Ctrl
miR-423-5p
2 days post-surgery
Subcapsular injection
miR-423-5p
Rel. expression (Fold change)
***
**
*
ns
10
1
Ctrl
Q1
Q2
Q3
Q4
miR-423-5p
2 days post-surgery
Q4
Q3
Q2
Q1
B
Blood urea nitrogen (BUN)
(mg/dL)
100
80
60
40
20
0
ns
*
*
Ctrl
miR-423-5p
0
2
7
14
21
days post-IR
C
ns
Tubular injury score
5
4
3
2
1
0
Ctrl
miR-423-5p
2 d post-IRI

Figure S7. MiR-423-5p promotes endothelial migration and angiogenesis. (**A**) Endothelial cells were transfected with Ctrl or miR-423-5p mimics (10 nM). Forty-eight hours post-transfection, miR-423-5p expression was measured by quantitative RT-PCR and presented as relative copies expression of miR-423-5p ± SEM after normalization with cel-miR-39; n = 3. (**B**) Endothelial cells were transfected with Ctrl or miR-423-5p mimics (10 nM). The expression of *HIF1A* and *VEGFA* mRNAs was measured by quantitative RT–PCR 48 h post-transfection. The results are presented as the relative expression of mRNA compared to cells transfected with mimic Ctrl (Ctrl) ± SEM after normalization with *HPRT1*; n = 6 for each condition. P values were obtained by unpaired t test (* P < 0.05, ** P < 0.01). (**C**) Endothelial cells transfected with Ctrl or miR-423-5p mimics and: (top) mechanically injured and percentage of open wound areas at 0 h was measured with TScratch software. Data are shown as % of the open wound area. (bottom) Capillary-like structures were quantified after 6 h on Matrigel. Angiogenic activity is assessed by quantifying the number of nodes, junctions and segment length per field ± SEM. n = 4 for each condition. P values obtained by unpaired t-test. Scale bar: 200 µm

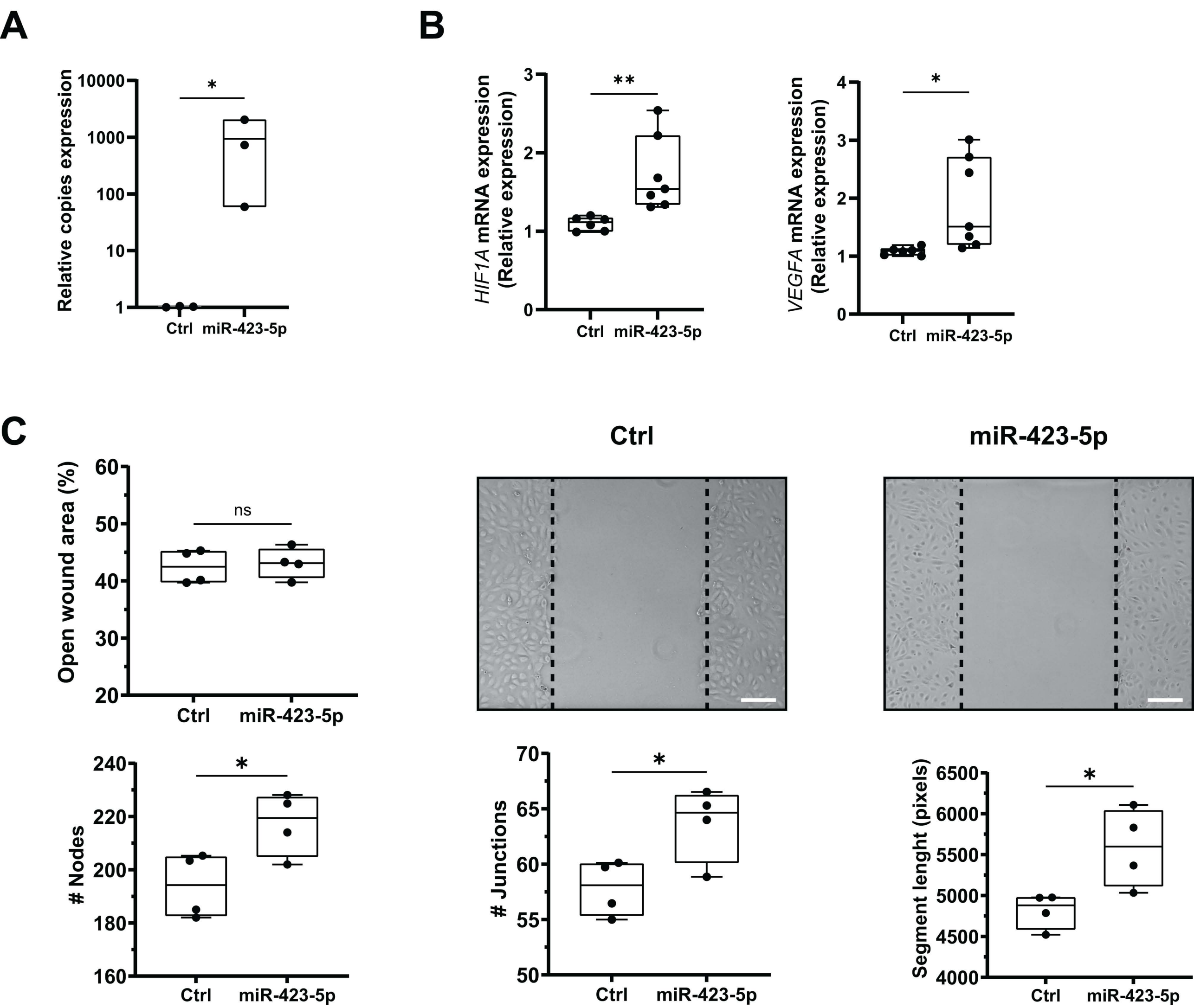
A
Relative copies expression
10000
1000
100
10
1
*
Ctrl
miR-423-5p
B
HIF1A mRNA expression (Relative expression)
3
2
1
0
**
Ctrl
miR-423-5p
VEGFA mRNA expression (Relative expression)
4
3
2
1
0
*
Ctrl
miR-423-5p
C
Open wound area (%)
60
50
40
30
20
ns
Ctrl
miR-423-5p
Ctrl
miR-423-5p
Nodes
240
220
200
180
160
*
Ctrl
miR-423-5p
Junctions
70
65
60
55
50
*
Ctrl
miR-423-5p
Segment lenght (pixels)
6500
6000
5500
5000
4500
4000
*
Ctrl
miR-423-5p

Figure S8. MiR-423-5p expression levels are increased following intramuscular injection. Expression of miR-423-5p in ischemic muscle following intramuscular injection of Ctrl or miR-423-5p mimics at 3-days post-surgery. Expression of miR-423-5p was measured by quantitative RT-PCR and the result is presented as expression fold change ± SEM. P value was obtained by unpaired t test (** $P < 0.01$); $n = 3$.

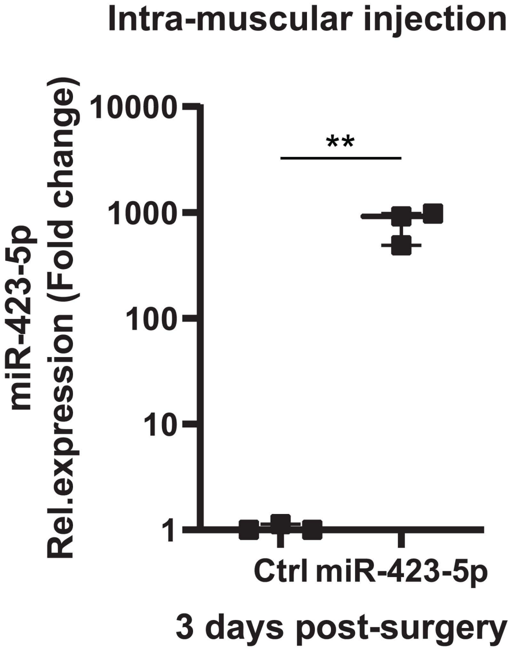
Intra-muscular injection
miR-423-5p
Rel.expression (Fold change)
10000
1000
100
10
1
**
Ctrl
miR-423-5p
3 days post-surgery
