# Supplementary material for: Endothelial extracellular vesicle miR-423-5p regulates microvascular homeostasis and renal function after ischemia-reperfusion injury
Source: JCI Insight. 2025 May 22;10(10):e181937. doi: 10.1172/jci.insight.181937 (PMC12128966; doi:10.1172/jci.insight.181937)

Original Immunoblots

Figure 1A

SDCBP

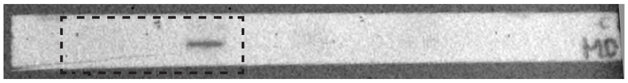

50,000 xg 200,000 xg

PSMA3

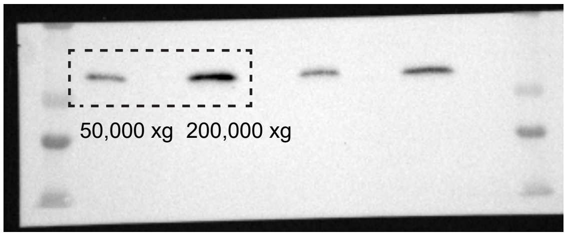

LG3

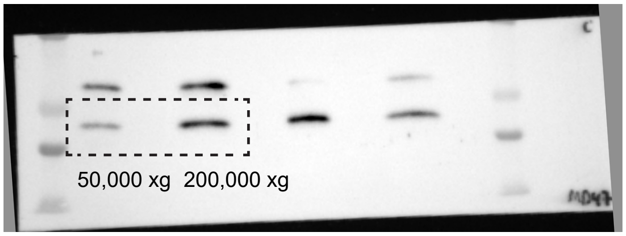

H3C1

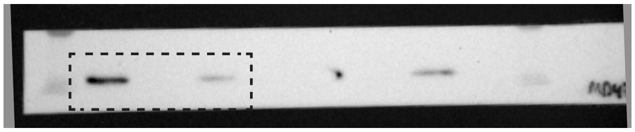

50,000 xg 200,000 xg

Original Immunoblots

Figure 3

MECA-32

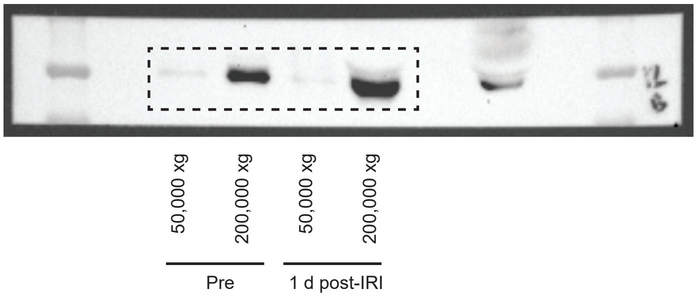

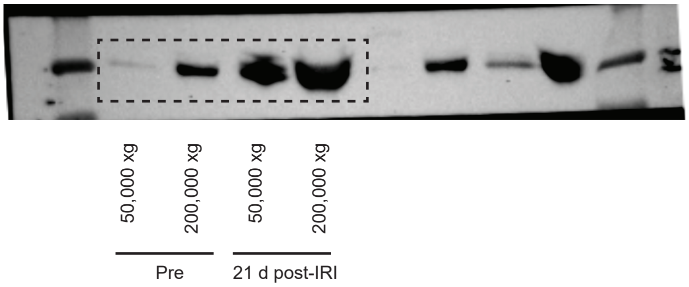

B-actin

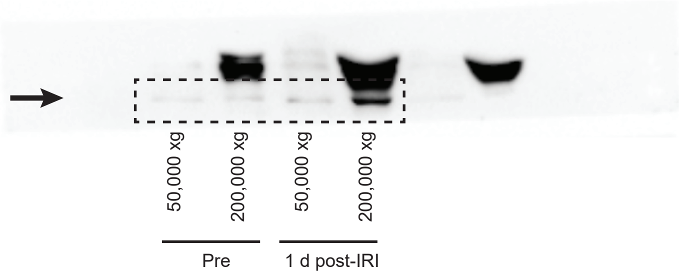

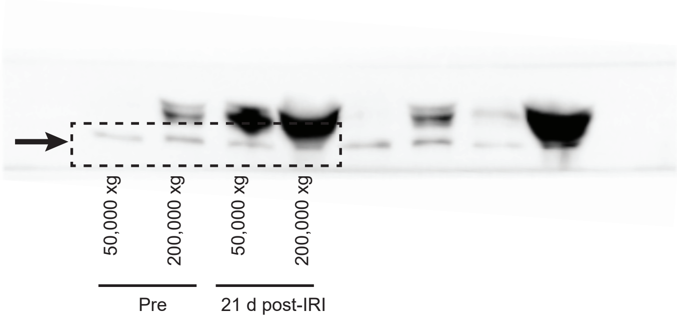

CD82

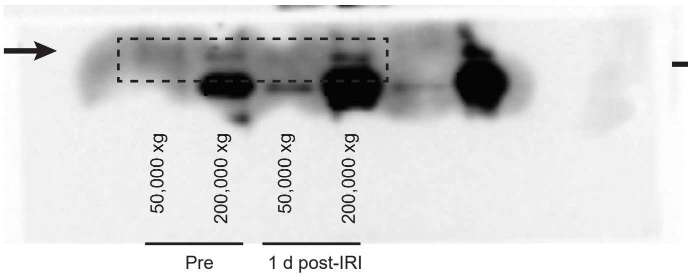

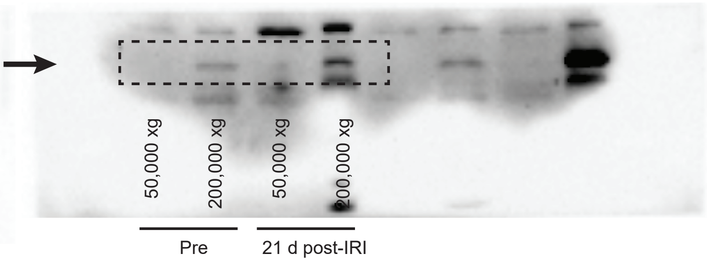

20S Proteasome

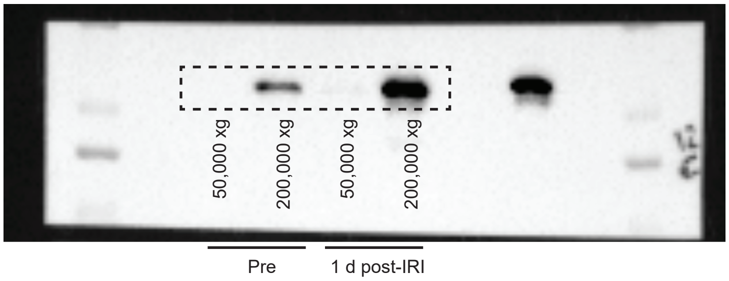

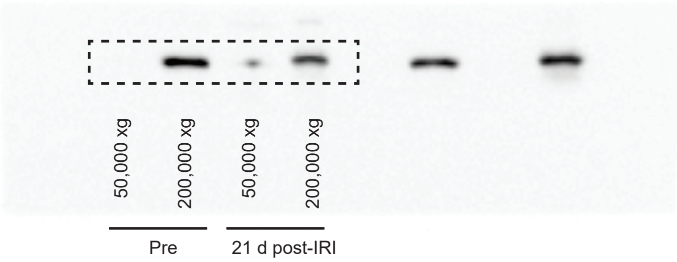

LG3

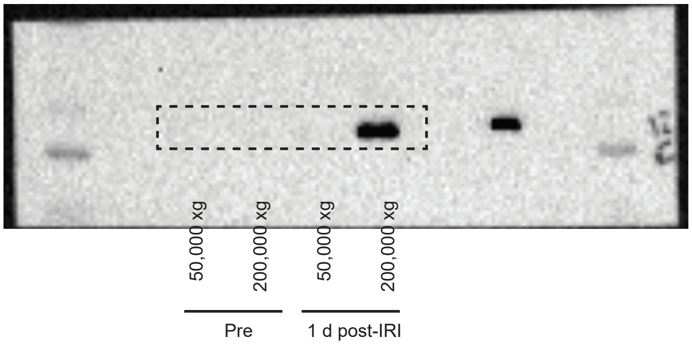

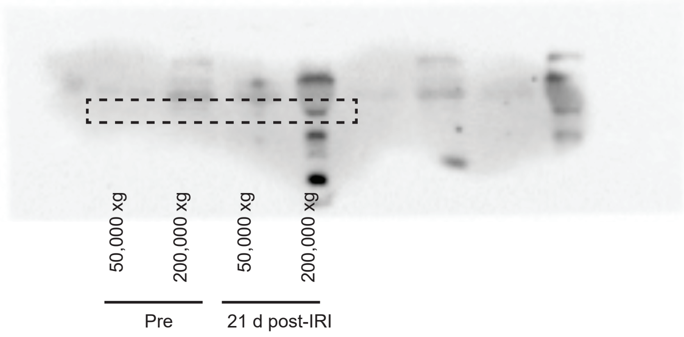

Original Immunoblots

Figure 5

CD31

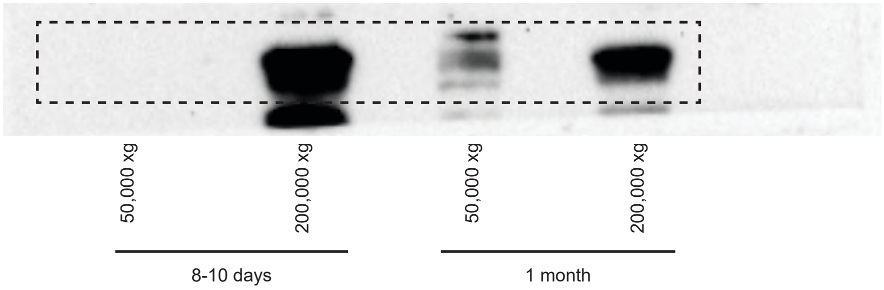

B-actin

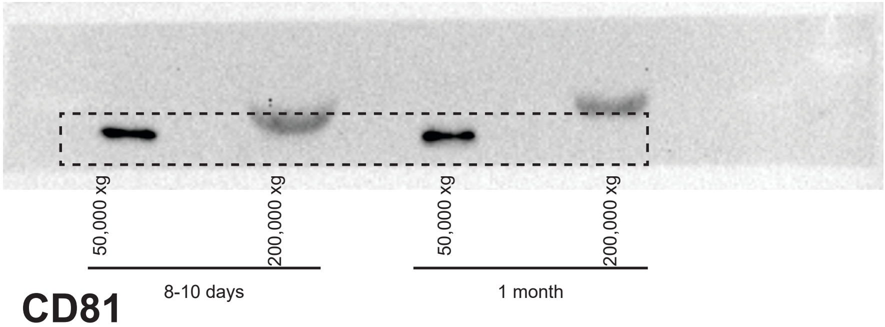

CD81

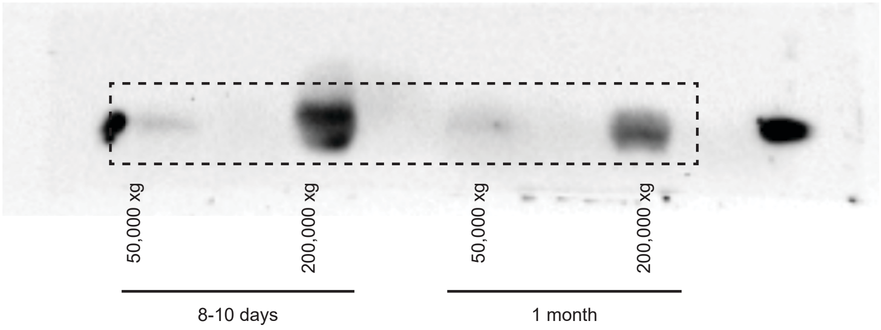

20S Proteasome

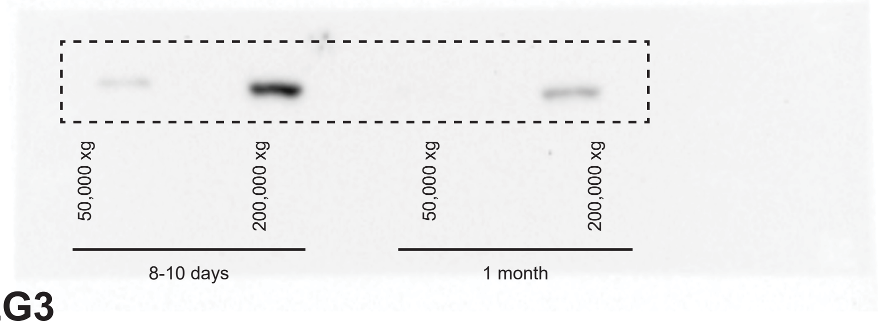

LG3

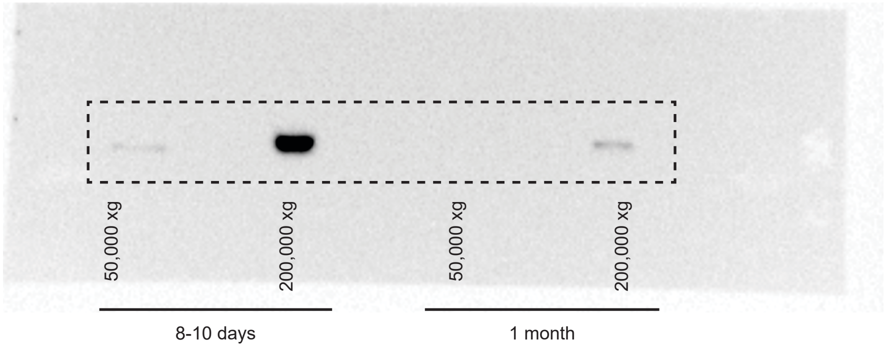

Original Immunoblots

Figure 8

Cleaved PARP

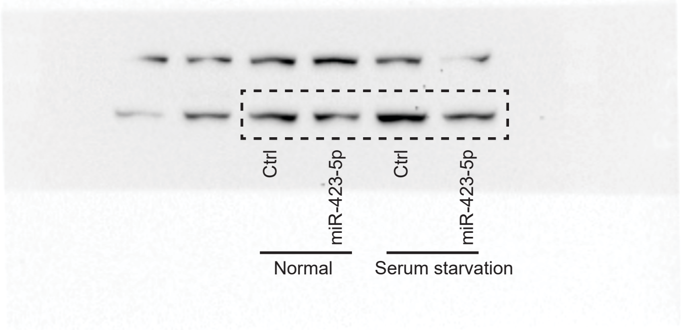

a-tubulin

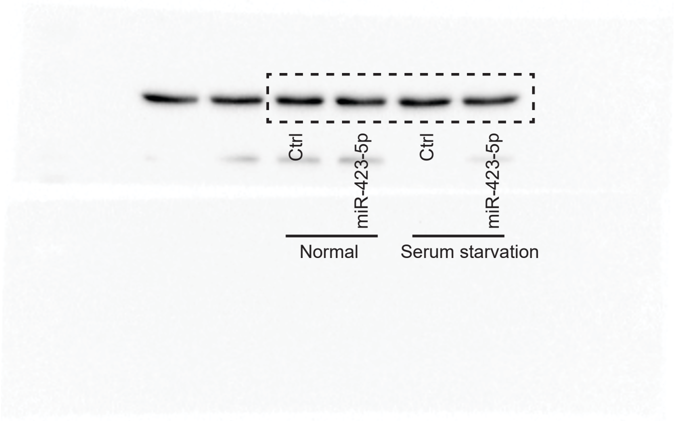

Original Immunoblots

Figure S1A

LG3

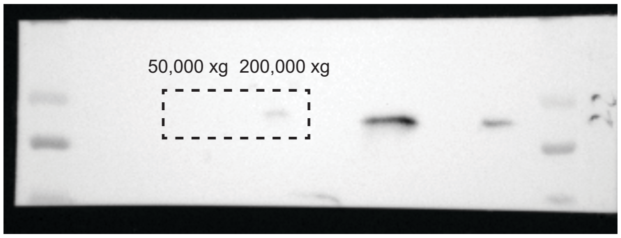

20S Proteasome

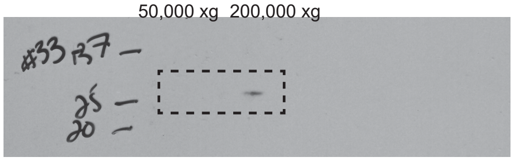

Syntenin-1

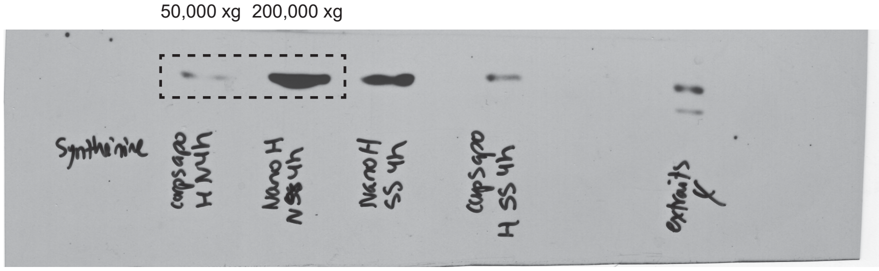

CD82

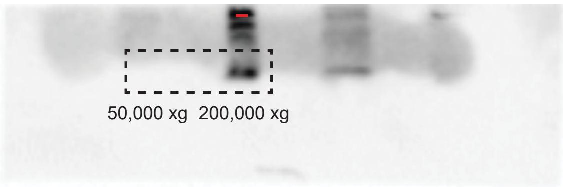

Figure S1B

20S Proteasome

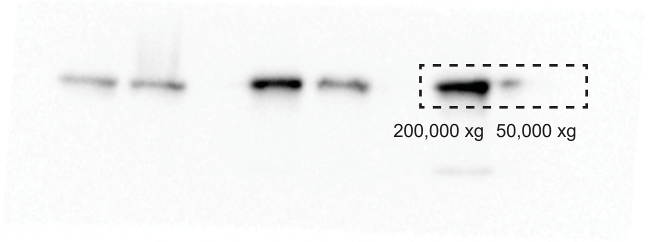

LG3

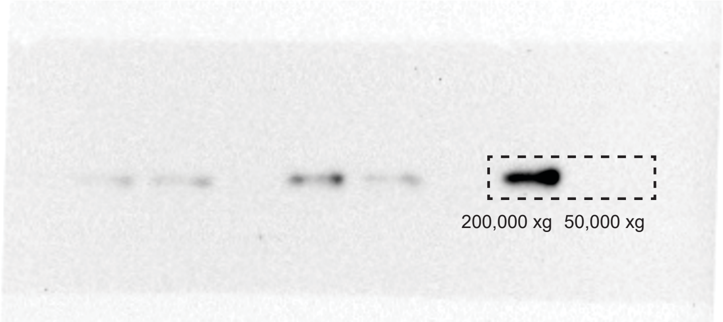

Syntenin-1

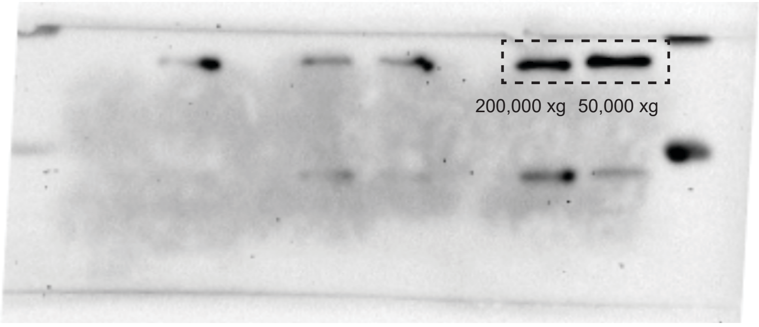

H3

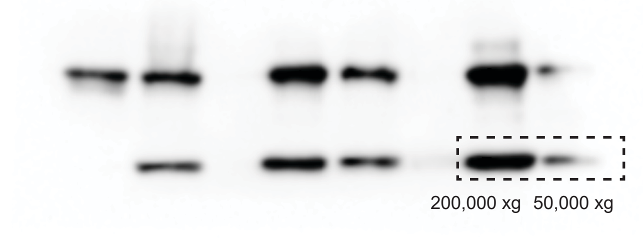

Original Immunoblots

Figure S3G

MECA-32

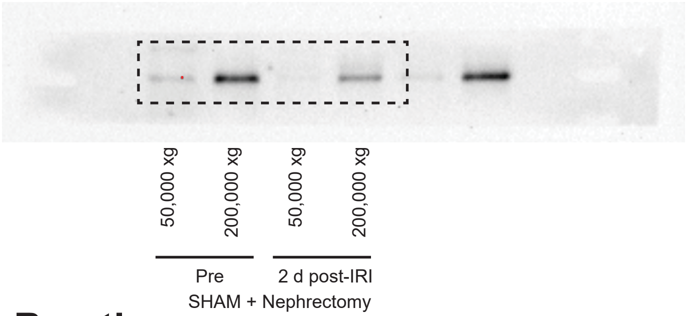

B-actin

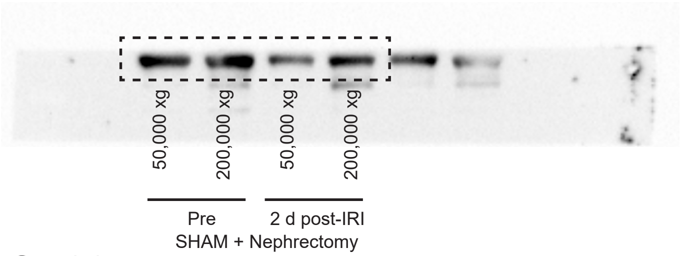

CD82

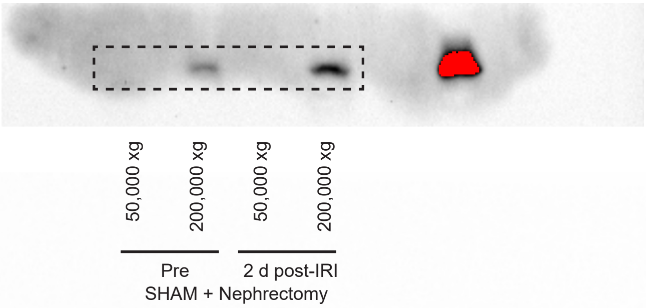

20S Proteasome

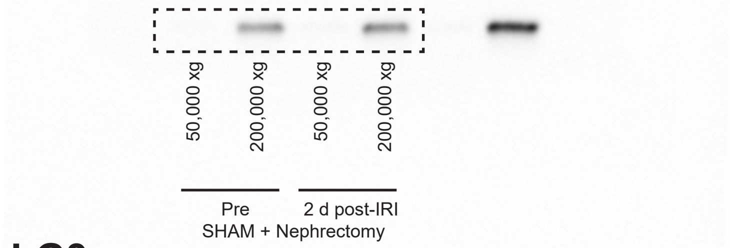

LG3

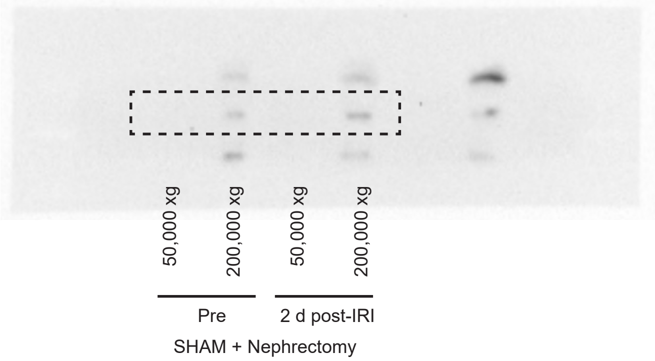

Supplement: Unedited blot and gel images [file jciinsight-10-181937-s223.pdf]
